# Conversion of Hydroxyproline “Doubly Customizable Units” to Hexahydropyrimidines: Access to Conformationally Constrained Peptides

**DOI:** 10.1021/acs.joc.3c00673

**Published:** 2023-07-10

**Authors:** Dácil Hernández, Marina Porras, Alicia Boto

**Affiliations:** Instituto de Productos Naturales y Agrobiología del CSIC, Avda. Astrofísico Fco. Sánchez, 3, La Laguna, Tenerife 38206, Spain

## Abstract

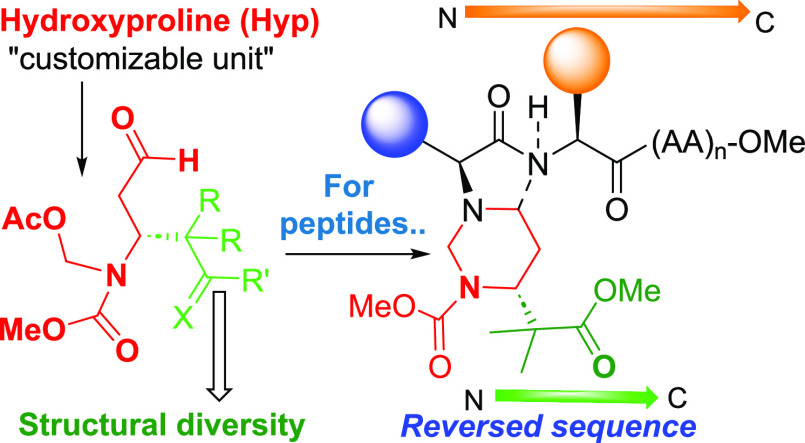

The efficient transformation of hydroxyproline “doubly customizable units” into rigid hexahydropyrimidine units takes place in good global yields and generates compounds of pharmaceutical interest. In particular, the process can readily provide access to peptidomimetics and peptides with reversed sequences or with valuable turns.

## Introduction

**Scheme 1:**
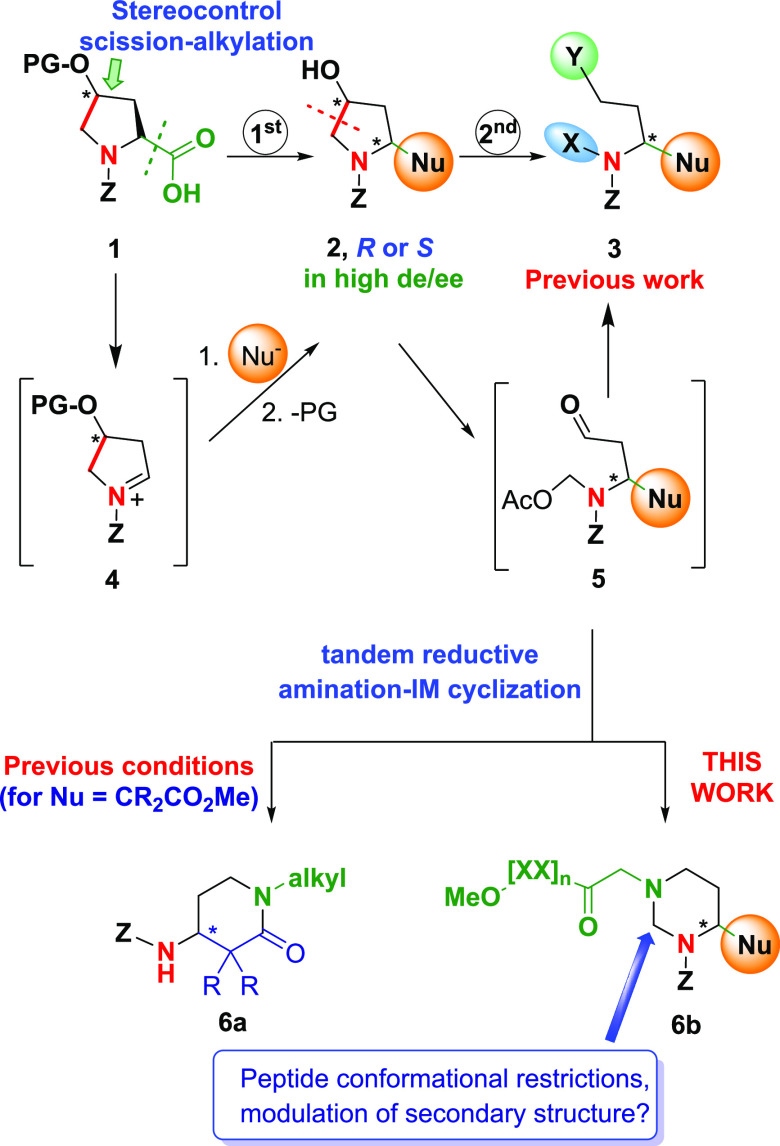
Previous Results and Current Work on Doubly Customizable Units

The recent introduction of hydroxyproline “doubly customizable units” allows the generation of a variety of modified amines and peptides in good yields and very few steps (Scheme 1, conversion **1 → 3**).^1,2^ A first modification (conversion **1 → [4] → 2**) is carried out using an oxidative radical decarboxylation, which generates acyliminium intermediate **4**. This ion reacts with carbon nucleophiles to afford 2-alkyl pyrrolidines in good yield and excellent 2*R* or 2*S* purity due to the control provided by the stereogenic center at C-4.^1a^ After deprotecting the 4-hydroxy group, compound **2** can undergo a second oxidative radical scission. The resulting products **5** have an *N*,*O*-acetal and an α-lateral chain with a terminal carbonyl group. Both chains can be functionalized independently,^1,3^ and thus, we have reported the formation of acyclic products **3** with three different, tailor-made substituents.^1a^

However, the possibility of using the newly formed chains to create other rigid, cyclic rings with new functionalities and conformational properties was very attractive. When the Nu group in compound **5** was an α- or β-ester (e.g., Nu = CO_2_Me or CH_2_CO_2_Me), treatment with a primary amine under reductive amination conditions provided an α- or β-amino lactam such as **6a**.^1b,3d^ However, the formation of a different heterocycle, using both the aldehyde and *N*,*O*-group, while Nu remained unaffected, had not been achieved. This transformation is not trivial, since the aldehyde group was the first to react, and the resulting linear chain extended away from the relatively bulky *N*-acetoxymethyl group. Therefore, non-cyclic products **3** were obtained.^1,3^ In this paper, we report a change in the reductive amination–cyclization conditions that allows the transformation of aldehydes **5** into hexahydropyrimidines **6b**, which in addition to their pharmacological utility, can be valuable units to modulate a peptide secondary structure.

**Scheme 2:**
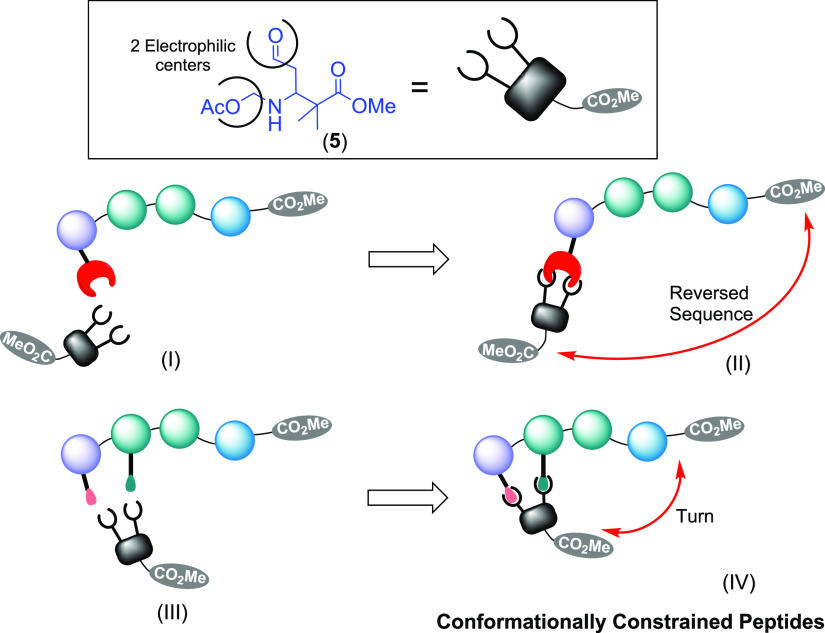
Use of In Situ-Formed Hexahydropyrimidines for Modulation of the Peptide Structure

Hexahydropyrimidines are valuable components of different drugs^4,5^ and have been usually prepared from a diamine derivative and an aldehyde,^4b,4c,5a,6^ although cycloadditions from imines have also been reported.^7^ This methodology works well for the preparation of simple hexahydropyrimidines but offers little selectivity when several reactive amine or amide groups are present in the molecule, particularly in complex ones such as peptides. Unlike the classical approach, the present methodology uses a dicarbonyl compound (substrate **5**) that reacts with an amine (as commented in Schemes 1 and 2). Since the carbonyl groups are selectively generated from the “customizable” residue before the reductive amination, the process allows a mild, selective introduction of the heterocycle in complex molecules.

In addition to the synthesis of hexahydropyrimidines, we were interested in their potential to modulate a peptide secondary structure. As shown in Scheme 2, the precursor dicarbonyl compound **5** can react with one or two amino groups (placed on the same or different residues) to create peptides with reversed sequences (conversion **I → II**) or to generate turns (conversion **III → IV**).

In fact, linear α,α-diamines have been introduced by Chorev and Goodman^8^ and others^9,10^ to obtain partial retro- and retroinverso peptides with promising bioactivities and superior resistance to proteases.^9,10^ However, using cyclic α,α-diamines (**II**) such as hexahydropyrimidines introduces extra rigidity in the system, which is valuable for a better control of biological interactions.

With respect to the formation of peptide turns (**IV**), our in situ-formed nitrogen heterocycles would serve as “turn templates”. The literature reports other natural and synthetic turn templates (such as morpholino-, piperazine-, indolizidine-, bicyclic lactam-, and dibenzofuran-containing amino acids, among others),^11−13^ but unlike them, the hexahydropyrimidines would be formed during peptide ligation, allowing in situ turn generation at specific positions. Since the starting aldehydes **5** can have *S* or *R* chains, the conformation of the molecule can be modulated. In the present paper, we will discuss the preliminary results and feasibility of this versatile strategy.

## Results and Discussion

**Scheme 3:**
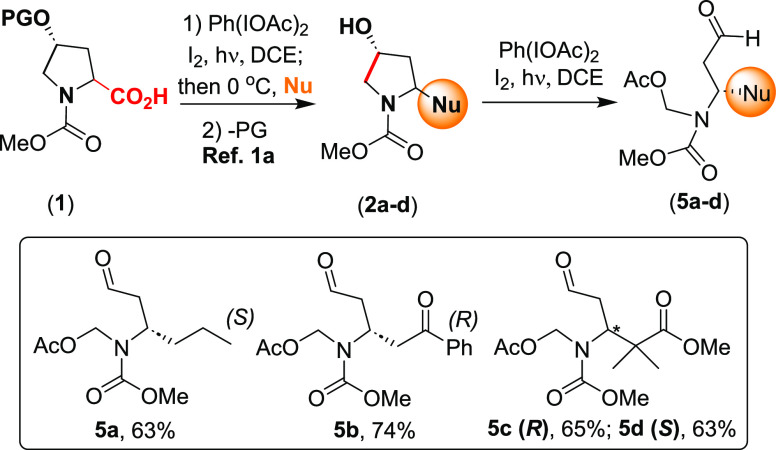
Oxidative Radical Scission of Substrates **2a**–**2d** to Give Pure Enantiomers **5a**–**5d**

The formation of the dicarbonyl compounds **5** from commercial or readily available l-hydroxyproline (Hyp) derivatives **1** is summarized in Scheme 3. The first transformation of the “doubly customizable” Hyp unit afforded 2-alkyl-4-hydroxypyrrolidines **2a**–**2d** in good yields (Scheme 3) via a sequential oxidative radical decarboxylation–alkylation, according to our reported methodology.^1a^ Similarly, the oxidative radical scission of compounds **2a**–**2d** to give aldehydes **5a**–**5d** as pure enantiomers also proceeded efficiently.

**Scheme 4:**
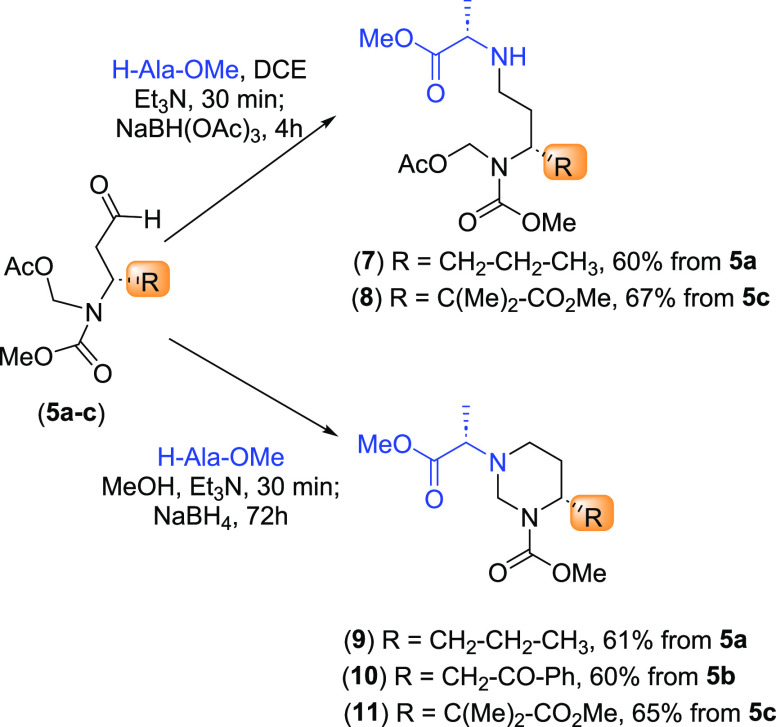
Reductive Amination of Scission Products **5a**–**5c**

Once the dicarbonyl substrates **5a**–**5c** were available, their reductive amination was studied using alanine methyl ester as a model amine (Scheme 4). When the reaction was carried out with NaBH(OAc)_3_ in dichloroethane, the expected amination took place to give products **7**–**8**. However, when the conditions were modified, and NaBH_4_ in methanol was introduced, the reductive amination was followed by an intramolecular cyclization. Under these conditions, the *N*-acetoxymethyl group was likely converted into an imino moiety, which underwent addition of the nearby amino group to give the hexahydropyrimidines **9**–**11**.

Particularly interesting is the α,β- peptide **11**, which presents not only the conformational constraints due to the dihydropyrimidine ring but also those induced by the α,α-dimethylated β-amino acid. In previous works, we observed that this moiety favors the formation of unusually expanded β-turns and γ- and δ-turns depending on its stereochemistry.^14^ Therefore, the following studies were devoted to the formation of hybrid peptides^15^ by reaction of substrate **5c** and its isomer **5d** with different amino acids and small peptides.

**Scheme 5:**
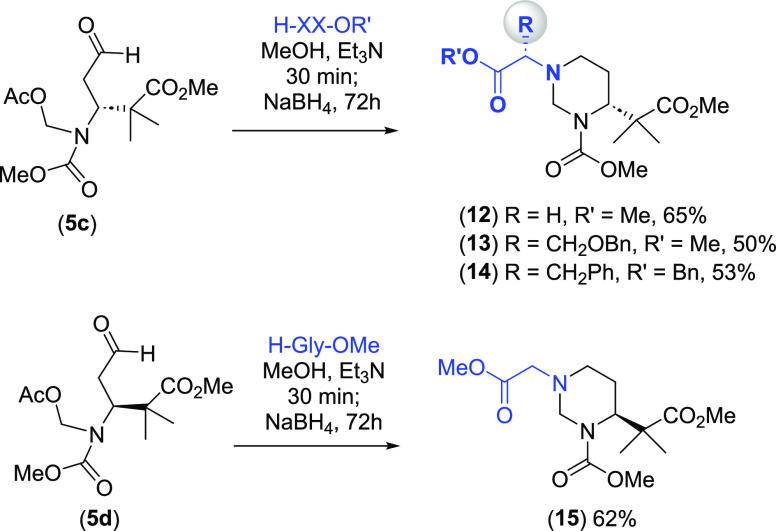
Synthesis of Hexahydropyrimidines with Different Reversed Sequences

Thus, aldehyde **5c** was reacted with derivatives of glycine, serine, and phenylalanine to give the hexahydropyrimidines **12**–**14** (Scheme 5), while the aldehyde isomer **5d** was treated with glycine methyl ester to afford product **15**. The formation of **12** and its epimer **15** took place in similar yield (65 and 62%, respectively), and all products **12**–**15** were easily purified. In these hexahydropyrimidine derivatives, two important structural changes are achieved: an increased system rigidity and also a reversal of the N → C direction, as will be commented later.

**Scheme 6:**
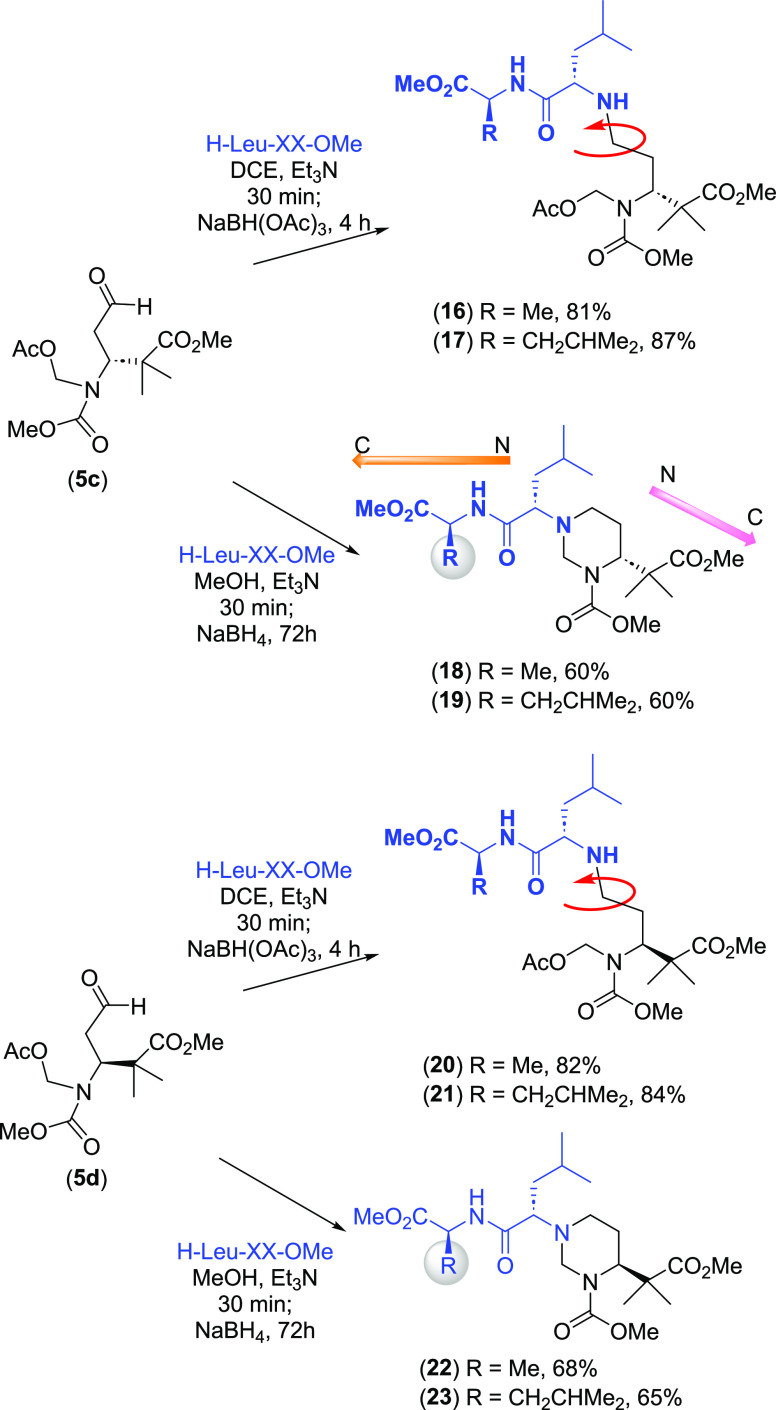
Synthesis of Hexahydropyrimidines in Peptides

The treatment of aldehydes **5c** and **5d** with peptides gave products with different flexibility (Scheme 6, compounds **16**–**23**) depending on the reductive amination conditions. Thus, peptides where all the side chains were linear (products **16**, **17**, **20**, and **21**) were obtained with the less polar system (triacetoxyborohydride in DCE and Et_3_N). However, peptides with a rigid hexahydropyrimidine core (products **18**, **19**, **22**, and **23**) were obtained with sodium borohydride in methanol. These heterocyclic compounds were obtained in 50–68% yield and were easily purified; again, the stereochemistry of the starting aldehyde **5c** or **5d** did not influence the reaction yields.

The different results obtained under both reaction conditions deserve some discussion. As commented in Introduction, we had only achieved cyclic products when compounds related to **5c** and **5d** were treated with a primary amine (such as benzylamine) under reductive amination conditions, giving amino lactams.^1b^ However, when an α-substituted primary amine was used (such as α-methylbenzylamine), the cyclization did not take place due to steric hindrance.^1b^ The amines derived from amino acids and peptides are also α-substituted primary amines and do not cyclize. Moreover, due to their superior bulkiness, they are likely away from the α,α-dimethyl ester chain and closer to the *N*-acetoxymethyl group. Under the more polar conditions, the cleavage of the acetoxymethyl group is favored with formation of an intermediate imine, which is intramolecularly trapped by the adjacent amine groups from the new amino acid or peptide chains, resulting in the hexahydropyrimidines. On the contrary, under the previous, less polar conditions, the acetoxy group remained intact and an acyclic compound was formed.

**Figure 1:**
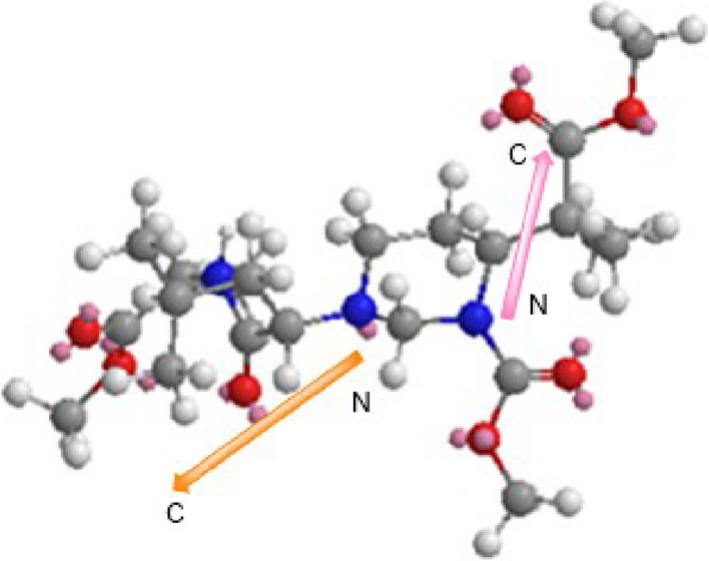
3D representation of the minimized conformation for compound **18**, showing reversed sequences.

Interestingly, as happened with the α,α-diamines of partially modified retro- and retroinverso peptides,^8−10^ a reversal of the N → C direction was achieved (shown for compound **18**, Figure 1). The 3D representation of an energy-minimized conformation for compound **18** (Figure 1) presents the reversed sequences, with the chains in different spatial orientations.^16^

**Scheme 7:**
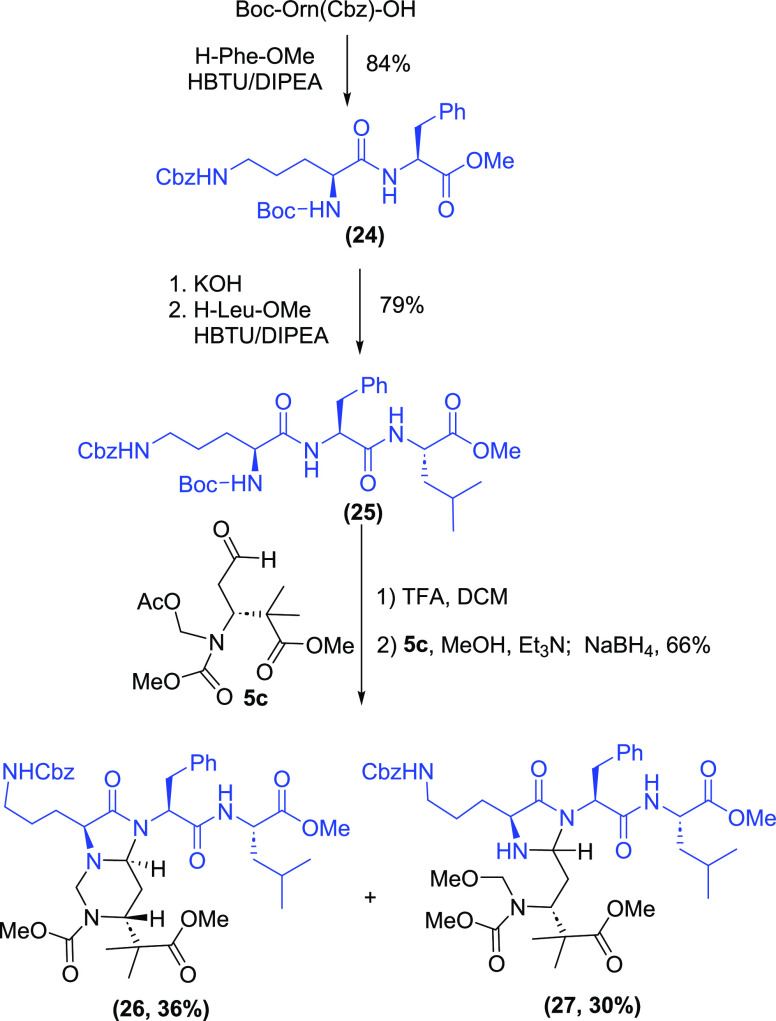
Turn-Inducing Hexahydropyrimidine **26**

Finally, the reaction of **5c** with tripeptide **25** under the reductive amination conditions was studied (Scheme 7). In this case, two products were obtained in 66% global yield, the valuable bicyclic hexahydropyrimidine **26** (36%), together with imidazolidine **27** (30%). The formation of compound **27** suggests that the formation of the imidazolidine ring takes place first, instead of the expected reductive amination. Then, the *N*,*O*-acetal is cleaved to an imine, followed by either addition of the ornithine α-amino group (to give **26**)^17^ or addition of the solvent (or sodium methoxide formed in situ) to give the *N*-methoxymethyl derivative **27**.^18^

**Figure 2:**
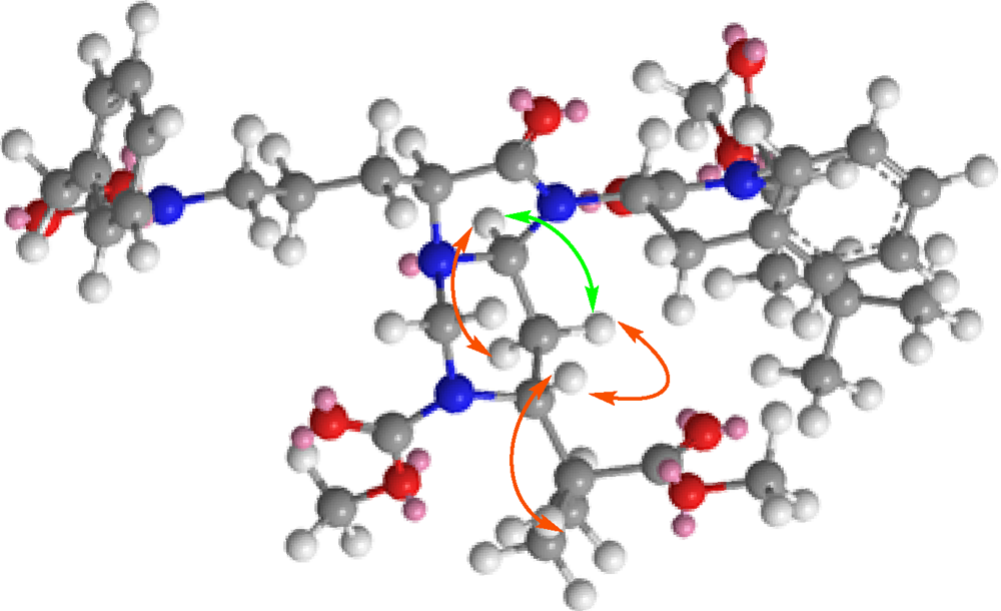
3D representation of the minimized conformation for compound **26** with NOESY correlations shown by arrows (strong interactions in orange and weak interactions in green). The rigid bicyclic unit is formed during peptide ligation and forces the peptide backbone to form a turn as shown.

The stereochemistry of compound **26** was determined with NOESY experiments (Figure 2) and by comparison of experimental *J*_H,H_ with theoretical ones for both isomers.^19^ The 3D representation of the energy-minimized structure whose *J*_H,H_ matched the experimental ones (Figure 2) shows both the β-amino acid and the α-peptide backbones in close proximity due to the system rigidity. Therefore, the bicyclic unit formed during peptide ligation could be effective for creating turns.

The stereochemistry of the monocyclic compound **27** could not be determined with NOESY experiments. No correlations were observed between the aminal proton and CHN_Orn_, but this result is not conclusive for opposite spatial orientation. Future work with other models is planned to solve this point. In any case, the possibility of obtaining systems with different rigidity levels is quite interesting for structural and biomedical studies. We are currently optimizing the cyclization conditions, using less nucleophilic solvents, and studying the reaction in larger peptide units, where new interactions and hydrogen bonds are expected. However, the current protocol already represents a promising tool to create conformationally constrained peptides and generate turn templates in situ, thus modulating the bioactivity of the peptides.

## Conclusions

The efficient conversion of hydroxyproline “doubly customizable units” into functionalized hexahydropyrimidines, heterocycles found in compounds of pharmaceutical interest, is presented herein. This work also introduces their use to provide access to peptidomimetics and peptides with reversed sequences or with valuable turns.

The hydroxyproline unit can be cleaved at two points, and a one-pot decarboxylation–alkylation process provided 2-substituted 4-hydroxypyrrolidines, which were used as substrates for the scission of the pyrrolidine ring at C_4_-C_5_. This strategy was developed in our preliminary paper to give compounds with three new linear chains or amino lactams, but in this work, the conditions were modified to attach the C-α and N^1^-substituents so that a hexahydropyrimidine ring was formed. This transformation was not trivial, since under most conditions, the newly formed chains extended away from each other, hindering the formation of cyclic compounds. Under the current new conditions, we achieved the generation of the rigid hexahydropyrimidine core, which proved valuable to create peptides with reversed sequence directions.

In addition, a further extension of this methodology created a hexahydropyrimidine-containing bicycle, which could be used as a “turn template” in peptides. Since the template was formed in situ during peptide ligation, this methodology would allow an easy access to peptides containing turns at selected positions while clipping two peptide chains.

## Experimental Section

### General Methods

Commercially available reagents and solvents were of analytical grade or were purified by standard procedures prior to use. All reactions involving air- or moisture-sensitive materials were carried out under a nitrogen atmosphere. Melting points were determined with a hot-stage apparatus and were uncorrected. Optical rotations were measured at the sodium line at ambient temperature (26 °C) in CHCl_3_ solutions. The NMR spectra were determined at 500 or 400 MHz for ^1^H and 125.7 or 100.6 MHz for ^13^C, at 26 °C or 70 °C, as stated for each case. Sometimes, due to slower rotamer interconversion at 26 °C, two (or more) sets of signals are visible at room temperature, while only one set of signals (rotamer average) is seen at 70 °C due to faster rotamer interconversion. For some compounds, the ^1^H NMR spectra show some signals as broad bands (br b) due to equilibria between rotamers.

Structural assignments were made with additional information from 2D experiments, such as COSY, HSQC, NOESY, and/or HMBC experiments.

The oxidative radical scissions were carried out at 26 °C under irradiation with a commercial (DIY shops) cool white LED light (14 W, 1100 lumens, 400–750 nm, with peaks at 432 nm/blue and 556 nm/green), placed 20 cm away from the standard (borosilicate) reaction flask. The LED light can be replaced by a commercial 80 W tungsten filament lamp, which provides light in a continuous spectrum of 340–1400 nm, which is perceived as white light, and which offers similar results.^1a^ In both cases, the reaction should be carried out until the disappearance of the starting material.

^1^H NMR spectra are reported as follows (s = singlet, d = doublet, t = triplet, dd = doublet of doublets, ddd = doublet of doublet of doublets, q = quartet, m = multiplet, br = broad, br b = broad band, and br s = broad singlet; coupling constant(s) in Hz). The mass spectra were carried out using electrospray ionization techniques (ESI) or electronic impact (EI); the latter was determined at 70 eV using an ion trap mass analyzer. Merck silica gel 60 PF_254_ and 60 (0.063–0.2 mm) were used for preparative thin layer chromatography and column chromatography, respectively. The reagent for TLC analysis was KMnO_4_ in NaOH/K_2_CO_3_ aqueous solution, and TLC was heated until the development of color.

### General Procedure for the Scission of the Pyrrolidine C_4_–C_5_ Bond

To a solution of the 2-alkyl-2-hydroxypyrrolidine (0.2 mmol) in dry dichloromethane (4 mL) were added iodine (25 mg, 0.1 mmol) and DIB (129 mg, 0.4 mmol). The resulting mixture was stirred for 3 h at 26 °C under irradiation with visible light (cool white LED lamp). Then, the reaction mixture was poured into 10% aqueous Na_2_S_2_O_3_ (10 mL) and extracted with CH_2_Cl_2_. The organic layer was dried over sodium sulfate, filtered, and concentrated under vacuum. The residue was purified by chromatography on silica gel (hexanes/ethyl acetate) to give the scission products **5a**–**5d**.

#### *N*-(Acetoxymethyl)-*N*-(methoxycarbonyl)-(3*S*)-aminohexanal (**5a**)

Obtained from 2-propyl-4-hydroxypyrrolidine **2a** (37 mg, 0.2 mmol) according to the general pyrrolidine scission procedure. After work-up and solvent evaporation, the residue was purified by radial chromatography (hexanes/EtOAc, 80:20), yielding aldehyde **5a** (30.5 mg, 63%) as a colorless viscous oil, whose characterization data were already reported.^1a^

#### (3*R*)-[*N*-(Acetoxymethyl)-*N*-(methoxycarbonyl)amino]-5-(oxo)-5-(phenyl)pentanal (**5b**)

Obtained from compound **2b** (53 mg, 0.2 mmol) according to the general pyrrolidine scission procedure. After work-up and solvent evaporation, the residue was purified by radial chromatography (hexanes/EtOAc, 80:20), yielding aldehyde **5b** (48 mg, 74%) as a colorless viscous oil, whose characterization data were already reported.^1a^

#### Methyl (3*R*)-[*N*-(Acetoxymethyl)-*N*-(methoxycarbonyl)amino]-2,2-dimethyl-5-oxopentanoate (**5c**)

Obtained from compound **2c** (49 mg, 0.2 mmol) according to the general pyrrolidine scission procedure. After work-up and solvent evaporation, the residue was purified by radial chromatography (hexanes/EtOAc, 80:20), yielding aldehyde **5c** (39 mg, 65%) as a colorless viscous oil, whose characterization data were already reported.^1a^

#### Methyl (3*S*)-[*N*-(Acetoxymethyl)-*N*-(methoxycarbonyl)amino]-2,2-dimethyl-5-oxopentanoate (**5d**)

Obtained from compound **2d** (49 mg, 0.2 mmol) according to the general pyrrolidine scission procedure. After work-up and solvent evaporation, the residue was purified by radial chromatography (hexanes/EtOAc, 70:30), yielding the aldehyde **5d** (38 mg, 63%) as a colorless viscous oil, whose characterization data were already reported.^1a^

### General Reductive Amination Procedure to Prepare Acyclic Compounds Such as Products **7** and **8**

A solution of aldehyde **5** (0.15 mmol) in dry dichloroethane (3 mL) was treated with the corresponding amine (0.21 mmol) and Et_3_N (28 μL, 20 mg, 0.2 mmol). The resulting mixture was stirred for 30 min, and then NaBH(OAc)_3_ (51 mg, 0.24 mmol) was added. The stirring continued for 4 h, and then the mixture was poured into saturated aqueous NaHCO_3_ and extracted with CH_2_Cl_2_. The organic layer was dried over sodium sulfate, filtered, and concentrated under vacuum. The residue was purified by chromatography on silica gel (hexanes/ethyl acetate) to give the reductive amination products, such as compounds **7** or **8**.

#### *N*-[(3*S*)-[*N*-(Acetoxymethyl)-*N*-(methoxycarbonyl)amino]hexyl]-L-alanine Methyl Ester (**7**)

Obtained from aldehyde **5a** (37 mg, 0.15 mmol) according to the general procedures for the reductive amination using **H-Ala-OMe** hydrochloride as a reagent (29.3 mg, 0.21 mmol). After work-up and solvent evaporation, the residue was purified by rotatory chromatography (hexanes/EtOAc, 40:60), yielding diamine **7** (30.1 mg, 60%) as a syrup. [α]_D_: +11 (c 0.07, CHCl_3_). IR (CHCl_3_) ν_max_ 3478, 1697, 1685, 1521, 1215, 1045, 928 cm^–1^. ^1^H NMR (400 MHz, 26 °C, CDCl_3_) rotamer mixture δ_H_ 5.40–5.20 (2H, m), 4.13/4.02 (1H/1H, br s/br s), 3.73 (3H, s), 3.71 (3H, s), 3.35–3.25 (2H, m), 2.60–2.55 (1H, m), 2.50–2.40 (1H, m), 2.05 (3H, s), 1.70–1.62 (3H, m), 1.50–1.37 (1H, m), 1.33–1.20 (2H, m), 1.27 (3H, d, *J* = 6.8 Hz), 0.88 (3H, t, *J* = 7.2 Hz). ^13^C{^1^H} NMR (125.7 MHz, 26 °C, CDCl_3_) rotamer mixture δc 176.1 (C), 170.6 (C, CO), 156.6 (C), 69.2 (CH_2_), 56.7 (CH), 54.8/54.6 (CH), 53.1/52.7 (CH_3_), 51.8/51.6 (CH_3_), 44.9/44.7 (CH_2_), 36.0/35.6 (CH_2_), 34.5/34.2 (CH_2_), 21.0 (CH_3_), 19.6/19.4 (CH_2_), 19.2/19.0 (CH_3_), 13.89/13.84 (CH_3_). HRMS (ESI-TOF) calcd for C_15_H_28_N_2_O_6_Na [M + Na]^+^, 355.1845; found, 355.1858. Anal. calcd for C_15_H_28_N_2_O_6_: C, 54.20; H, 8.49; N, 8.43. Found: C, 54.37; H, 8.31; N, 8.62.

#### Methyl *N*-[*N*-(Acetoxymethyl)-*N*-(methoxycarbonyl)-2,2-dimethyl-1-methyl-D-β-norvalin-5-yl]-L-alanine (**8**)

Obtained from aldehyde **5c** (45.5 mg, 0.15 mmol) according to the general procedures for the reductive amination using **H-Ala-OMe** hydrochloride as a reagent (29.3 mg, 0.21 mmol). After work-up and solvent evaporation, the residue was purified by rotatory chromatography (hexanes/EtOAc, 40:60), yielding diamine **8** (39.2 mg, 67%) as a syrup. [α]_D_: +8 (c 0.39, CHCl_3_). IR (CHCl_3_) ν_max_ 3438, 1727, 1699, 1213, 1047 cm^–1^. ^1^H NMR (500 MHz, 26 °C, CDCl_3_) rotamer mixture δ_H_ 5.43–5.23 (2H, m), 4.60–4.40 (1H, m), 3.71 (3H, s), 3.68 (3H, s), 3.64 (3H, s), 3.32–3.23 (1H, m), 2.67–2.58 (1H, m), 2.43–2.30 (1H, m), 2.04 (3H, s), 1.90–1.80 (1H, m), 1.76–1.62 (1H, br b), 1.25 (3H, d, *J* = 6.5 Hz), 1.20 (3H, s), 1.16 (3H, s). ^13^C{^1^H} NMR (125.7 MHz, 26 °C, CDCl_3_) rotamer mixture δc 176.5 (C), 175.9 (C), 170.4 (C), 157.5/157.3 (C), 69.5/69.1 (CH_2_), 59.9/59.6 (CH), 56.6 (CH), 53.4 (CH_3_), 52.0 (CH_3_), 51.8 (CH_3_), 47.1 (C), 45.2/44.9 (CH_2_), 29.3/29.1 (CH_2_), 24.2 (CH_3_), 22.1/21.7 (CH_3_), 21.0 (CH_3_), 19.0 (CH_3_). HRMS (ESI-TOF) calcd for C_17_H_30_N_2_O_8_Na [M + Na]^+^, 413.1900; found, 413.1904. Anal. calcd for C_17_H_30_N_2_O_8_: C, 52.30; H, 7.75; N, 7.18. Found: C, 52.38; H, 7.86; N, 7.13.

### General Procedure for Reductive Amination–Cyclization of Aldehydes **5a**–**5d** to Prepare Hexahydropyrimidines

A solution of the aldehyde (0.15 mmol) in dry methanol (3 mL) was treated with the corresponding amine (1.4 equiv, 0.21 mmol) and Et_3_N (28 μL, 20 mg, 0.20 mmol). The resulting mixture was stirred for 1 h, and then NaBH_4_ (7.4 mg, 0.195 mmol) was added. The stirring continued for 72 h, and then the reaction mixture was poured into saturated aqueous NaHCO_3_ and extracted with CH_2_Cl_2_. The organic layer was dried over sodium sulfate, filtered, and evaporated under vacuum. The residue was purified by chromatography on silica gel (hexanes/ethyl acetate mixtures) to give the hexahydropyrimidines.

#### Methyl 2*S*-(1-Methoxycarbonyl-6*S*-(propyl)hexahydropyrimidin-3-yl)propanoate (**9**)

Obtained from aldehyde **5a** (37 mg, 0.15 mmol) according to the general procedures for the reductive amination–cyclization using **H-Ala-OMe** hydrochloride as a reagent (29.3 mg, 0.21 mmol). After work-up and solvent evaporation, the residue was purified by rotatory chromatography (hexanes/EtOAc, 50:50), yielding compound **9** (24.8 mg, 61%) as a syrup. [α]_D_: +7 (c 0.29, CHCl_3_). IR (CHCl_3_) ν_max_ 1715, 1683, 1450, 1296, 1029 cm^–1^. ^1^H NMR (400 MHz, CDCl_3_, 26 °C) rotamer mixture δ_H_ 4.75–4.67 (1H, m), 4.30–4.20 (1H, m), 3.78–3.67 (1H, m), 3.72 (3H, s), 3.68 (3H, s), 3.47 (1H, dt, *J* = 6.8, 7.2 Hz), 2.89–2.82 (1H, m), 2.76 (1H, dd, *J* = 12.4, 11.2 Hz), 1.97–1.86 (1H, m), 1.75–1.65 (1H, m), 1.46–1.36 (2H, m), 1.32 (3H, d, *J* = 7.2 Hz), 1.31–1.22 (2H, m), 0.92 (3H, t, *J* = 7.4 Hz). ^13^C{^1^H} NMR (100.6 MHz, CDCl_3_, 26 °C) rotamer mixture δc 173.5 (C), 155.9 (C), 59.0 (CH_2_ + CH), 52.5 (CH_3_), 51.6 (CH_3_), 49.6 (CH), 42.0 (CH_2_), 31.9 (CH_2_), 27.0 (CH_2_), 19.2 (CH_2_), 15.6 (CH_3_), 13.9 (CH_3_). HRMS (ESI-TOF) calcd for C_13_H_24_N_2_O_4_Na [M + Na]^+^, 295.1634; found, 295.1622. Anal. calcd for C_13_H_24_N_2_O_4_: C, 57.33; H, 8.88; N, 10.29. Found: C, 57.68; H, 8.49; N, 10.08.

#### Methyl 2*S*-(1-Methoxycarbonyl-6*R*-(2-oxo-2-phenylethyl)hexahydropyrimidin-3-yl)propanoate (**10**)

Obtained from aldehyde **5b** (48.2 mg, 0.15 mmol) according to the general procedures for the reductive amination–cyclization using **H-Ala-OMe** hydrochloride as a reagent (29.3 mg, 0.21 mmol). After work-up and solvent evaporation, the residue was purified by rotatory chromatography (hexanes/EtOAc, 50:50), yielding compound **10** (31.2 mg, 60%) as a syrup. [α]_D_: −9 (c 0.15, CHCl_3_). IR (CHCl_3_) ν_max_ 1733, 1697, 1449, 1230, 1045 cm^–1^. ^1^H NMR (500 MHz, CD_3_CN, 70 °C) δ_H_ 7.97 (2H, d, *J* = 7.1 Hz), 7.62 (1H, dd, *J* = 7.5, 7.3 Hz), 7.51 (2H, dd, *J* = 7.9, 7.5 Hz), 4.79–4.74 (1H, m), 4.67 (1H, d, *J* = 11.7 Hz), 3.93 (1H, d, *J* = 11.9 Hz), 3.69 (3H, s), 3.56 (3H, s), 3.47 (1H, q, *J* = 7.1 Hz), 3.38 (1H, dd, *J* = 15.8, 8.0 Hz), 3.24 (1H, dd, *J* = 15.7, 6.3 Hz), 2.89–2.84 (1H, m), 2.80 (1H, ddd, *J* = 12.5, 12.5, 3.0 Hz), 1.98–1.90 (1H, m), 1.55 (1H, dq, *J* = 13.7, 2.8 Hz), 1.27 (3H, d, *J* = 7.0 Hz). ^13^C{^1^H} NMR (125.7 MHz, CD_3_CN, 70 °C) δc 199.6 (C), 174.5 (C), 156.7 (C), 138.6 (C), 134.3 (CH), 129.9 (2 × CH), 129.3 (2 × CH), 60.7 (CH), 60.5 (CH_2_), 53.2 (CH_3_), 52.2 (CH_3_), 48.8 (CH), 43.5 (CH_2_), 40.6 (CH_2_), 28.4 (CH_2_), 16.0 (CH_3_). HRMS (ESI-TOF) calcd for C_18_H_24_N_2_O_5_Na [M + Na]^+^, 371.1583; found, 371.1585. Anal. calcd for C_18_H_24_N_2_O_5_: C, 62.05; H, 6.94; N, 8.04. Found: C, 62.17; H, 6.65; N, 7.85.

#### Methyl 2*S*-(1-Methoxycarbonyl-6*R*-(1-methoxy-2-methyl-1-oxopropan-2-yl)hexahydropyrimidin-3-yl)propanoate (**11**)

Obtained from aldehyde **5c** (49.5 mg, 0.15 mmol) according to the general procedures for the reductive amination–cyclization using **H-Ala-OMe** hydrochloride as a reagent (29.3 mg, 0.21 mmol). After work-up and solvent evaporation, the residue was purified by rotatory chromatography (hexanes/EtOAc, 50:50), yielding compound **11** (32.1 mg, 65%) as a syrup. [α]_D_: +16 (c 0.37, CHCl_3_). IR (CHCl_3_) ν_max_ 3438, 1723, 1698, 1449, 1218, 1047 cm^–1^. ^1^H NMR (500 MHz, CD_3_CN, 70 °C) δ_H_ 4.68 (1H, d, *J* = 12.6 Hz), 4.26 (1H, t, *J* = 6.6 Hz), 3.93 (1H, d, *J* = 12.6 Hz), 3.67 (3H, s), 3.65 (3H, s), 3.64 (3H, s), 3.40 (1H, q, *J* = 7.1 Hz), 2.79 (1H, ddd, *J* = 12.0, 8.0, 4.4 Hz), 2.70–2.64 (1H, m), 1.88–1.83 (2H, m), 1.23 (1H, d, *J* = 7.0 Hz), 1.20 (3H, s), 1.19 (3H, s). ^13^C{^1^H} NMR (125.7 MHz, CD_3_CN, 25 °C) δc 178.0 (C), 174.8 (C), 158.5 (C), 61.1 (CH), 60.9 (CH_2_), 58.1 (CH), 53.4 (CH_3_), 52.7 (CH_3_), 52.2 (CH_3_), 48.3 (C), 44.7 (CH_2_), 24.6 (CH_3_), 24.3 (CH_2_), 23.2 (CH_3_), 16.3 (CH_3_). HRMS (ESI-TOF) calcd for C_15_H_26_N_2_O_6_Na [M + Na]^+^, 353.1689; found, 353.1689. Anal. calcd for C_15_H_26_N_2_O_6_: C, 54.53; H, 7.93; N, 8.48. Found: C, 54.72; H, 7.76; N, 8.57.

#### Methyl 2-(1-Methoxycarbonyl-6*R*-(1-methoxy-2-methyl-1-oxopropan-2-yl)hexahydropyrimidin-3-yl)acetate (**12**)

Obtained from aldehyde **5c** (49.5 mg, 0.15 mmol) according to the general procedures for the reductive amination–cyclization using **H-Gly-OMe** hydrochloride as a reagent (26.4 mg, 0.21 mmol). After work-up and solvent evaporation, the residue was purified by rotatory chromatography (hexanes/EtOAc, 50:50), yielding compound **12** (30.8 mg, 65%) as a syrup. [α]_D_: +39 (c 0.17, CHCl_3_). IR (CHCl_3_) ν_max_ 1733, 1697, 1449, 1230, 1045 cm^–1^. ^1^H NMR (500 MHz, CD_3_CN, 70 °C) δ_H_ 4.71 (1H, d, *J* = 13.0 Hz), 4.29 (1H, t, *J* = 6.5 Hz), 3.96 (1H, d, *J* = 13.0 Hz), 3.66 (3H, s), 3.65 (3H, s), 3.64 (3H, s), 3.36 (1H, d, *J* = 16.7 Hz), 3.25 (1H, d, *J* = 16.7 Hz), 2.92 (1H, ddd, *J* = 12.0, 9.5, 4.0 Hz), 2.57 (1H, ddd, *J* = 11.9, 7.0, 4.5 Hz), 1.97–1.89 (1H, m), 1.80–1.72 (1H, m), 1.21 (3H, s), 1.20 (3H, s). ^13^C{^1^H}NMR (125.7 MHz, CD_3_CN, 25 °C) δc 178.2 (C), 172.3 (C), 158.8 (C), 62.2 (CH_2_), 58.0 (CH), 56.3 (CH_2_), 53.8 (CH_3_), 53.0 (CH_3_), 52.6 (CH_3_), 49.1 (CH_2_), 48.4 (C), 24.6 (CH_3_), 23.2 (CH_2_), 23.1 (CH_3_). HRMS (ESI-TOF) calcd for C_14_H_24_N_2_O_6_Na [M + Na]^+^, 339.1532; found, 339.1529. Anal. calcd for C_14_H_24_N_2_O_6_: C, 53.15; H, 7.65; N, 8.86. Found: C, 52.89; H, 7.80; N, 8.56.

#### Methyl 3-Benzyloxy-2*S*-(1-methoxycarbonyl-6*R*-(1-methoxy-2-methyl-1-oxopropan-2-yl)hexahydropyrimidin-3-yl)propanoate (**13**)

Obtained from aldehyde **5c** (49.5 mg, 0.15 mmol) according to the general procedures for the reductive amination–cyclization using **H-Ser(OBn)-OMe** hydrochloride as a reagent (49.0 mg, 0.21 mmol). After work-up and solvent evaporation, the residue was purified by rotatory chromatography (hexanes/EtOAc, 50:50), yielding compound **13** (32.5 mg, 50%) as a syrup. [α]_D_: +8 (c 0.64, CHCl_3_). IR (CHCl_3_) ν_max_ 1723, 1517, 1421, 1218, 1054 cm^–1^. ^1^H NMR (500 MHz, CD_3_CN, 25 °C) rotamer mixture δ_H_ 7.40–7.33 (5H, m), 5.14 (2H, s), 4.66 (1H, d, *J* = 11.2 Hz), 4.23 (1H, t, *J* = 5.9 Hz), 4.00 (1H, br dd, *J* = 10.3, 2.0 Hz), 3.73 (1H, dd, *J* = 11.3, 5.8 Hz), 3.68–3.64 (1H, m), 3.60 (9H, s), 3.43 (1H, t, *J* = 6.3 Hz), 2.80–2.75 (1H, m), 2.73–2.67 (1H, m), 1.87–1.76 (2H, m), 1.19/1.14 (3H/3H, s/s), 1.16/1.12 (3H/3H, s/s). ^13^C{^1^H} NMR (125.7 MHz, CD_3_CN, 25 °C) rotamer mixture δc 178.0 (C), 172.2 (C), 158.6 (C), 137.7 (C), 129.9 (2 × CH), 129.5/129.4 (2 × CH), 129.3 (CH), 68.2/67.8 (CH), 67.6/67.4 (CH_2_), 61.6 (CH_2_), 61.3 (CH_2_), 58.1 (CH), 53.6/53.5 (CH_3_), 52.8/52.7 (CH_3_), 52.7/52.6 (CH_3_), 48.4 (C), 45.0 (CH_2_), 24.65/24.57 (CH_2_), 23.5/23.3 (CH_3_), 22.4 (CH_3_). HRMS (ESI-TOF) calcd for C_21_H_29_N_2_O_7_ [M + 2H – Me]^+^, 423.2158; found, 423.2123. Anal. calcd for C_22_H_32_N_2_O_7_: C, 60.54; H, 7.39; N, 6.42. Found: C, 60.77; H, 7.16; N, 6.30.

#### Methyl 2*S*-(1-Methoxycarbonyl-6*R*-(1-methoxy-2-methyl-1-oxopropan-2-yl)hexahydropyrimidin-3-yl)-3-phenylpropanoate (**14**)

Obtained from aldehyde **5c** (49.5 mg, 0.15 mmol) according to the general procedures for the reductive amination–cyclization using **H-Phe-OBn** hydrochloride as a reagent (61.3 mg, 0.21 mmol). After work-up and solvent evaporation, the residue was purified by rotatory chromatography (hexanes/EtOAc, 50:50), yielding compound **14** (38.3 mg, 53%) as a syrup. [α]_D_: +5 (c 0.72, CHCl_3_). IR (CHCl_3_) ν_max_ 1723, 1702, 1449, 1221, 1047 cm^–1^. ^1^H NMR (500 MHz, CD_3_CN, 70 °C) δ_H_ 7.37–7.18 (10H, m), 5.06 (2H, s), 4.70 (1H, d, *J* = 12.1 Hz), 4.26 (1H, t, *J* = 6.6 Hz), 3.95 (1H, d, *J* = 12.2 Hz), 3.68–3.62 (1H, m), 3.62 (3H, s), 3.59 (3H, s), 3.03 (1H, dd, *J* = 13.8, 8.2 Hz), 2.94 (1H, dd, *J* = 13.9, 6.9 Hz), 2.86–2.82 (1H, m), 1.83 (2H, q, *J* = 6.2 Hz), 1.17 (3H, s), 1.15 (3H, s). ^13^C{^1^H} NMR (125.7 MHz, CD_3_CN, 70 °C) δc 178.0 (C), 172.8 (C), 158.5 (C), 139.6 (C), 137.6 (C), 130.5 (2 × CH), 129.8 (2 × CH), 129.6 (2 × CH), 129.5 (2 × CH), 129.4 (CH), 127.6 (CH), 68.0 (CH), 67.3 (CH_2_), 61.7 (CH_2_), 57.9 (CH), 53.5 (CH_3_), 52.7 (CH_3_), 48.4 (C), 44.8 (CH_2_), 36.9 (CH_2_), 24.8 (CH_2_), 24.6 (CH_3_), 23.3 (CH_3_). HRMS (ESI-TOF) calcd for C_27_H_34_N_2_O_6_Na [M + Na]^+^, 505.2315; found, 505.2315. Anal. calcd for C_27_H_34_N_2_O_6_: C, 67.20; H, 7.10; N, 5.81. Found: C, 67.04; H, 7.47; N, 5.83.

#### Methyl 2-(1-Methoxycarbonyl-6*S*-(1-methoxy-2-methyl-1-oxopropan-2-yl)hexahydropyrimidin-3-yl)acetate (**15**)

Obtained from aldehyde **5d** (49.5 mg, 0.15 mmol) according to the general procedures for the reductive amination–cyclization using **H-Gly-OMe** hydrochloride as a reagent (26.4 mg, 0.21 mmol). After work-up and solvent evaporation, the residue was purified by rotatory chromatography (hexanes/EtOAc, 50:50), yielding tetrahydropyrimidine **15** (29.0 mg, 62%) as a syrup. [α]_D_: −41 (c 0.15, CHCl_3_). IR (CHCl_3_) ν_max_ 1733, 1697, 1449, 1230, 1045 cm^–1^. ^1^H NMR (500 MHz, CD_3_CN, 70 °C) δ_H_ 4.71 (1H, d, *J* = 13.0 Hz), 4.29 (1H, dd, *J* = 7.0, 6.0 Hz), 3.96 (1H, d, *J* = 13.0 Hz), 3.66 (3H, s), 3.65 (3H, s), 3.64 (3H, s), 3.36 (1H, d, *J* = 16.6 Hz), 3.25 (1H, d, *J* = 16.6 Hz), 2.92 (1H, ddd, *J* = 12.5, 9.4, 3.6 Hz), 2.57 (1H, ddd, *J* = 11.5, 6.8, 4.5 Hz), 1.97–1.89 (1H, m), 1.80–1.74 (1H, m), 1.21 (3H, s), 1.20 (3H, s). ^13^C{^1^H} NMR (125.7 MHz, CD_3_CN, 25 °C) δc 178.2 (C), 172.3 (C), 158.8 (C), 62.2 (CH_2_), 58.0 (CH), 56.3 (CH_2_), 53.8 (CH_3_), 53.0 (CH_3_), 52.6 (CH_3_), 49.1 (CH_2_), 48.4 (C), 24.6 (CH_3_), 23.2 (CH_2_), 23.1 (CH_3_). HRMS (ESI-TOF) calcd for C_14_H_24_N_2_O_6_Na [M + Na]^+^, 339.1532; found, 339.1530. Anal. calcd for C_14_H_24_N_2_O_6_: C, 53.15; H, 7.65; N, 8.86. Found: C, 53.40; H, 7.37; N, 8.74.

#### Methyl *N*-[*N*-(Acetoxymethyl)-*N*-(methoxycarbonyl)-2,2-dimethyl-1-methyl-D-β-norvalin-5-yl]-L-leucinyl-L-alaninate (**16**)

Obtained from aldehyde **5c** (45.5 mg, 0.15 mmol) according to the general procedure for the reductive amination using **H-Leu-Ala-OMe** hydrochloride (53 mg, 0.21 mmol) as a reagent. After work-up and solvent evaporation, the residue was purified by rotatory chromatography (hexanes/EtOAc, 40:60), yielding diamine **16** (60.9 mg, 81%) as a syrup. [α]_D_: +14 (c 0.22, CHCl_3_). IR (CHCl_3_) ν_max_ 3433, 1723, 1523, 1226, 1046 cm^–1^. ^1^H NMR (500 MHz, 70 °C, CD_3_CN) δ_H_ 7.55–7.40 (1H, br b), 5.38 (1H, d, *J* = 11.0 Hz), 5.23 (1H, d, *J* = 11.5 Hz), 4.50–4.37 (2H, m), 3.70 (3H, s), 3.69 (3H, s), 3.65 (3H, s), 2.99 (1H, dd, *J* = 8.5, 5.5 Hz), 2.60–2.47 (2H, m), 1.99 (3H, s), 1.90–1.79 (1H, m), 1.75–1.68 (2H, m), 1.47–1.41 (1H, m), 1.39–1.34 (1H, m), 1.37 (3H, d, *J* = 6.9 Hz), 1.22 (3H, s), 1.19 (3H, s), 0.94 (3H, d, *J* = 6.5 Hz), 0.91 (3H, d, *J* = 6.5 Hz). ^13^C{^1^H} NMR (100.6 MHz, 26 °C, CDCl_3_) rotamer mixture δc 177.6 (C), 174.8/174.5 (C/C), 173.5 (C), 170.2 (C), 157.6/157.3 (C/C), 69.4/69.2 (CH_2_), 61.7 (CH), 59.4/59.2 (CH), 53.5 (CH_3_), 52.3 (CH_3_), 52.0 (CH_3_), 47.2 (CH), 46.9 (C), 45.9/45.6 (CH_2_), 42.9 (CH_2_), 29.3/28.7 (CH), 24.9 (CH_2_), 24.1 (CH_3_), 23.3 (CH_3_), 22.1 (CH_3_), 21.7 (CH_3_), 20.9 (CH_3_), 18.3 (CH_3_). HRMS (ESI-TOF) calcd for C_23_H_41_N_3_O_9_Na [M + Na]^+^, 526.2740; found, 526.2743. Anal. calcd for C_23_H_41_N_3_O_9_: C, 54.86; H, 8.21; N, 8.34. Found: C, 55.17; H, 8.22; N, 8.46.

#### Methyl *N*-[*N*-(Acetoxymethyl)-*N*-(methoxycarbonyl)-2,2-dimethyl-1-methyl-D-β-norvalin-5-yl]-L-leucinyl-L-leucinate (**17**)

Obtained from aldehyde **5c** (45.5 mg, 0.15 mmol) according to the general procedure for the reductive amination using **H-Leu-Leu-OMe** hydrochloride (61.5 mg, 0.21 mmol) as a reagent. After work-up and solvent evaporation, the residue was purified by rotatory chromatography (hexanes/EtOAc, 40:60), yielding diamine **17** (70.8 mg, 87%) as a syrup. [α]_D_: +17 (c 0.17, CHCl_3_). IR (CHCl_3_) ν_max_ 3488, 1733, 1719, 1702, 1217, 1050 cm^–1^. ^1^H NMR (400 MHz, 70 °C, CD_3_CN) δ_H_ 7.50–7.32 (1H, br b), 5.41 (1H, d, *J* = 11.2 Hz), 5.28 (1H, d, *J* = 11.2 Hz), 4.52–4.45 (2H, m), 3.73 (3H, s), 3.71 (3H, s), 3.68 (3H, s), 3.03 (1H, dd, *J* = 8.2, 5.8 Hz), 2.63–2.50 (2H, m), 2.01 (3H, s), 1.90–1.80 (1H, m), 1.79–1.68 (3H, m), 1.69–1.64 (2H, m), 1.51–1.35 (2H, m), 1.25 (3H, s), 1.22 (3H, s), 0.98 (3H, d, *J* = 6.0 Hz), 0.97 (3H, d, *J* = 6.8 Hz), 0.95 (3H, d, *J* = 6.4 Hz), 0.94 (3H, d, *J* = 6.0 Hz). ^13^C{^1^H} NMR (100.6 MHz, 70 °C, CD_3_CN) δc 177.8 (C), 176.1 (C), 174.6 (C), 171.2 (C), 158.7 (C), 71.2 (CH_2_), 63.1 (CH), 61.8 (CH), 54.0 (CH_3_), 52.8 (CH_3_), 52.7 (CH_3_), 51.6 (CH), 48.4 (C), 47.1 (CH_2_), 44.5 (CH_2_), 42.1 (CH_2_), 30.2 (CH_2_), 26.12 (CH), 26.06 (CH), 24.7 (CH_3_), 23.7 (CH_3_), 23.5 (CH_3_), 23.0 (CH_3_), 22.7 (CH_3_), 22.3 (CH_3_), 21.4 (CH_3_). HRMS (ESI-TOF) calcd for C_26_H_47_N_3_O_9_Na [M + Na]^+^, 568.3210; found, 568.3209. Anal. calcd for C_26_H_47_N_3_O_9_: C, 57.23; H, 8.68; N, 7.70. Found: C, 57.29; H, 8.59; N, 7.80.

#### Methyl [2*S*-(1-Methoxycarbonyl-6*R*-(1-methoxy-2-methyl-1-oxopropan-2-yl)hexahydropyrimidin-3-yl)-4-methylpentanoyl]-L-alanine (**18**)

Obtained from aldehyde **5c** (49.5 mg, 0.15 mmol) according to the general procedures for the reductive amination–cyclization using **H-Leu-Ala-OMe** as a reagent (45.0 mg, 0.21 mmol). After work-up and solvent evaporation, the residue was purified by rotatory chromatography (hexanes/EtOAc, 40:60), yielding hexahydropyrimidine **18** (40.3 mg, 60%) as a syrup. [α]_D_: +21 (c 0.48, CHCl_3_). IR (CHCl_3_) ν_max_ 3438, 1720, 1705, 1513, 1218, 1049 cm^–1^. ^1^H NMR (500 MHz, CD_3_CN, 70 °C) δ_H_ 7.07 (1H, br s), 4.72 (1H, d, *J* = 12.4 Hz), 4.41 (1H, quin, *J* = 7.3 Hz), 4.28 (1H, dd, *J* = 5.4, 7.1 Hz), 3.82 (1H, d, *J* = 12.4 Hz), 3.69 (3H, s), 3.66 (3H, s), 3.64 (3H, s), 3.10 (1H, dd, *J* = 5.8, 8.5 Hz), 2.83–2.77 (1H, m), 2.71–2.65 (1H, m), 1.89–1.82 (2H, m), 1.68–1.60 (1H, m), 1.54–1.42 (2H, m), 1.36 (3H, d, *J* = 7.2 Hz), 1.21 (3H, s), 1.20 (3H, s), 0.92 (3H, d, *J* = 6.2 Hz), 0.91 (3H, d, *J* = 6.2 Hz). ^13^C{^1^H} NMR (125.7 MHz, CD_3_CN, 25 °C) δc 178.1 (C), 174.5 (C), 173.4 (C), 158.5 (C), 65.7 (CH), 61.1 (CH_2_), 57.9 (CH), 53.5 (CH_3_), 52.9 (CH_3_), 52.7 (CH_3_), 49.0 (CH), 48.3 (C), 46.1 (CH_2_), 39.6 (CH_2_), 26.4 (CH), 24.8 (CH_3_), 24.6 (CH_2_), 23.8 (CH_3_), 23.4 (CH_3_), 22.8 (CH_3_), 18.3 (CH_3_). HRMS (ESI-TOF) calcd for C_21_H_37_N_3_O_7_Na [M + Na]^+^, 466.2529; found, 466.2529. Anal. calcd for C_21_H_37_N_3_O_7_: C, 56.87; H, 8.41; N, 9.47. Found: C, 57.08; H, 8.51; N, 9.58.

#### Methyl [2*S*-(1-Methoxycarbonyl-6*R*-(1-methoxy-2-methyl-1-oxopropan-2-yl)hexahydropyrimidin-3-yl)-4-methylpentanoyl]-L-leucine (**19**)

Obtained from aldehyde **5c** (49.5 mg, 0.15 mmol) according to the general procedures for the reductive amination–cyclization using **H-Leu-Leu-OMe** as a reagent (54.2 mg, 0.21 mmol). After work-up and solvent evaporation, the residue was purified by rotatory chromatography (hexanes/EtOAc, 40:60), yielding compound **19** (43.6 mg, 60%) as a syrup. [α]_D_: +22 (c 0.45, CHCl_3_). IR (CHCl_3_) ν_max_ 3434, 1723, 1520, 1449, 1211, 1054 cm^–1^. ^1^H NMR (500 MHz, CD_3_CN, 70 °C) δ_H_ 6.94 (1H, d, *J* = 7.4 Hz), 4.73 (1H, d, *J* = 12.4 Hz), 4.43 (1H, td, *J* = 8.1, 6.8 Hz), 4.28 (1H, dd, *J* = 7.2, 5.4 Hz), 3.83 (1H, d, *J* = 12.4 Hz), 3.67 (3H, s), 3.66 (3H, s), 3.64 (3H, s), 3.10 (1H, dd, *J* = 8.0, 6.6 Hz), 2.79 (1H, ddd, *J* = 12.4, 9.3, 3.9 Hz), 2.70–2.65 (1H, m), 1.89–1.81 (2H, m), 1.73–1.64 (1H, m), 1.65–1.59 (3H, m), 1.48–1.45 (2H, m), 1.21 (3H, s), 1.20 (3H, s), 0.95 (3H, d, *J* = 6.5 Hz), 0.93 (6H, d, *J* = 6.5 Hz), 0.90 (3H, d, *J* = 6.5 Hz). ^13^C{^1^H} NMR (125.7 MHz, CD_3_CN, 70 °C) δc 178.0 (C), 174.4 (C), 173.8 (C), 158.6 (C), 65.9 (CH), 61.3 (CH_2_), 57.9 (CH), 53.5 (CH_3_), 52.8 (CH_3_), 52.7 (CH_3_), 51.7 (CH), 48.3 (C), 46.0 (CH_2_), 41.8 (CH_2_), 40.1 (CH_2_), 26.4 (CH), 26.0 (CH), 24.7 (CH_3_), 24.6 (CH_2_), 23.8 (CH_3_), 23.42 (CH_3_), 23.36 (CH_3_), 22.8 (CH_3_), 22.2 (CH_3_). HRMS (ESI-TOF) calcd for C_24_H_43_N_3_O_7_Na [M + Na]^+^, 508.2999; found, 508.2999. Anal. calcd for C_24_H_43_N_3_O_7_: C, 59.36; H, 8.93; N, 8.65. Found: C, 59.46; H, 8.73; N, 9.63.

#### *N*-[*N*-(Acetoxymethyl)-*N*-(methoxycarbonyl)-2,2-dimethyl-1-methyl-L-β-norvalin-5-yl]-L-leucinyl-L-alanine Methyl Ester (**20**)

Obtained from aldehyde **5d** (45.5 mg, 0.15 mmol) according to the general procedure for the reductive amination using **H-Leu-Ala-OMe** hydrochloride (53 mg, 0.21 mmol) as a reagent. After work-up and solvent evaporation, the residue was purified by rotatory chromatography (hexanes/EtOAc, 40:60), yielding diamine **20** (61.7 mg, 82%) as a syrup. [α]_D_: −5 (c 0.42, CHCl_3_). IR (CHCl_3_) ν_max_ 3434, 1704, 1570, 1450, 1295, 1088 cm^–1^. ^1^H NMR (400 MHz, 70 °C, CD_3_CN) δ_H_ 7.42–7.32 (1H, br b), 5.38 (1H, d, *J* = 10.8 Hz), 5.27 (1H, d, *J* = 11.2 Hz), 4.50–4.37 (2H, m), 3.69 (6H, s), 3.65 (3H, s), 3.02 (1H, dd, *J* = 7.9, 5.7 Hz), 2.55 (2H, dd, *J* = 7.6, 6.0 Hz), 1.99 (3H, s), 1.92–1.83 (1H, m), 1.77–1.66 (2H, m), 1.49–1.44 (1H, m), 1.40–1.33 (1H, m), 1.37 (3H, d, *J* = 7.2 Hz), 1.22 (3H, s), 1.19 (3H, s), 0.95 (3H, d, *J* = 6.4 Hz), 0.92 (3H, d, *J* = 6.8 Hz). ^13^C{^1^H} NMR (100.6 MHz, 70 °C, CD_3_CN) δc 177.8 (C), 175.9 (C), 174.6 (C), 171.3 (C), 164.3 (C), 71.3 (CH_2_), 62.6 (CH), 61.6 (CH), 54.0 (CH_3_), 52.9 (CH_3_), 52.7 (CH_3_), 49.0 (CH), 48.5 (C), 46.8 (CH_2_), 44.5 (CH_2_), 30.1 (CH_2_), 26.1 (CH), 24.7 (CH_3_), 23.7 (CH_3_), 23.0 (CH_3_), 22.7 (CH_3_), 21.4 (CH_3_), 18.3 (CH_3_). HRMS (ESI-TOF) calcd for C_23_H_41_N_3_O_9_Na [M + Na]^+^, 526.2741; found, 526.2737. Anal. calcd for C_23_H_41_N_3_O_9_: C, 54.86; H, 8.21; N, 8.34. Found: C, 54.83; H, 8.31; N, 8.20.

#### *N*-[*N*-(Acetoxymethyl)-*N*-(methoxycarbonyl)-2,2-dimethyl-1-methyl-L-β-norvalin-5-yl]-L-leucinyl-L-leucine Methyl Ester (**21**)

Obtained from aldehyde **5d** (45.5 mg, 0.15 mmol) according to the general procedure for the reductive amination using **H-Leu-Leu-OMe** hydrochloride as a reagent (61.5 mg, 0.21 mmol). After work-up and solvent evaporation, the residue was purified by rotatory chromatography (hexanes/EtOAc, 40:60), yielding diamine **21** (69.0 mg, 84%) as a syrup. [α]_D_: −8 (c 0.48, CHCl_3_). IR (CHCl_3_) ν_max_ 3436, 1704, 1570, 1450, 1295, 1088 cm^–1^. ^1^H NMR (400 MHz, 70 °C, CD_3_CN) δ_H_ 7.30–7.24 (1H, m), 5.38 (1H, d, *J* = 11.2 Hz), 5.27 (1H, d, *J* = 11.2 Hz), 4.50–4.40 (2H, m), 3.69 (3H, s), 3.68 (3H, s), 3.65 (3H, s), 3.03 (1H, dd, *J* = 7.8, 5.9 Hz), 2.60–2.52 (2H, m), 1.99 (3H, s), 1.90–1.80 (1H, br b), 1.78–1.62 (6H, m), 1.51–1.44 (1H, m), 1.41–1.31 (1H, m), 1.22 (3H, s), 1.19 (3H, s), 0.95 (3H, d, *J* = 6.4 Hz), 0.94 (3H, d, *J* = 6.8 Hz), 0.92 (6H, d, *J* = 6.4 Hz). ^13^C{^1^H} NMR (100.6 MHz, 70 °C, CD_3_CN) δc 177.8 (C), 176.1 (C), 174.5 (C), 171.3 (C), 158.7 (C), 71.2 (CH_2_), 62.7 (CH), 61.7 (CH), 54.0 (CH_3_), 52.8 (CH_3_), 52.7 (CH_3_), 51.7 (CH), 48.5 (C), 46.8 (CH_2_), 44.6 (CH_2_), 42.0 (CH_2_), 30.1 (CH_2_), 26.2 (2 × CH), 24.7 (CH_3_), 23.7 (CH_3_), 23.5 (CH_3_), 23.1 (CH_3_), 22.7 (CH_3_), 22.3 (CH_3_), 21.4 (CH_3_). HRMS (ESI-TOF) calcd for C_26_H_47_N_3_O_9_Na [M + Na]^+^, 568.3210; found, 568.3215. Anal. calcd for C_26_H_47_N_3_O_9_: C, 57.23; H, 8.68; N, 7.70. Found: C, 57.30; H, 8.63; N, 7.72.

#### Methyl [2*S*-(1-Methoxycarbonyl-6*R*-(1-methoxy-2-methyl-1-oxopropan-2-yl)hexahydropyrimidin-3-yl)-4-methylpentanoyl]-L-alanine (**22**)

Obtained from aldehyde **5d** (49.5 mg, 0.15 mmol) according to the general procedures for the reductive amination–cyclization using **H**-**Leu-Ala-OMe** as a reagent (45.0 mg, 0.21 mmol). After work-up and solvent evaporation, the residue was purified by rotatory chromatography (hexanes/EtOAc, 40:60), yielding compound **22** (45.0 mg, 68%) as a syrup. [α]_D_: −21 (c 0.52, CHCl_3_). IR (CHCl_3_) ν_max_ 3438, 1718, 1700, 1447, 1214, 1047 cm^–1^. ^1^H NMR (500 MHz, CD_3_CN, 70 °C) δ_H_ 6.94 (1H, d, *J* = 5.8 Hz), 4.82 (1H, d, *J* = 12.8 Hz), 4.38 (1H, quint, *J* = 7.2 Hz), 4.28 (1H, dd, *J* = 7.6, 4.7 Hz), 3.89 (1H, d, *J* = 12.8 Hz), 3.68 (3H, s), 3.66 (3H, s), 3.64 (3H, s), 3.09 (1H, dd, *J* = 8.4, 6.1 Hz), 2.80 (1H, ddd, *J* = 12.0, 9.8, 3.6 Hz), 2.62 (1H, dt, *J* = 12.0, 6.0 Hz), 1.95–1.89 (1H, m), 1.83–1.78 (1H, m), 1.68–1.62 (1H, m), 1.58–1.46 (2H, m), 1.35 (3H, d, *J* = 7.3 Hz), 1.21 (3H, s), 1.20 (3H, s), 0.93 (3H, d, *J* = 6.5 Hz), 0.91 (3H, d, *J* = 6.6 Hz). ^13^C{^1^H} NMR (125.7 MHz, CD_3_CN, 70 °C) δc 178.1 (C), 174.4 (C), 173.4 (C), 158.3 (C), 65.4 (CH), 59.3 (CH_2_), 57.8 (CH), 53.4 (CH_3_), 52.8 (CH_3_), 52.7 (CH_3_), 49.1 (CH), 48.3 (C), 47.3 (CH_2_), 39.7 (CH_2_), 26.1 (CH), 24.8 (CH_3_), 24.3 (CH_2_), 23.7 (CH_3_), 23.4 (CH_3_), 22.9 (CH_3_), 18.3 (CH_3_). HRMS (ESI-TOF) calcd for C_21_H_37_N_3_O_7_Na [M + Na]^+^, 466.2529; found, 466.2526. Anal. calcd for C_21_H_37_N_3_O_7_: C, 56.87; H, 8.41; N, 9.47. Found: C, 56.76; H, 8.62; N, 9.24.

#### Methyl [2*S*-(1-Methoxycarbonyl-6*R*-(1-methoxy-2-methyl-1-oxopropan-2-yl)hexahydropyrimidin-3-yl)-4-methylpentanoyl]-L-leucine (**23**)

Obtained from aldehyde **5d** (49.5 mg, 0.15 mmol) according to the general procedures for the reductive amination–cyclization using **H-Leu-Leu-OMe** as a reagent (54.0 mg, 0.21 mmol). After work-up and solvent evaporation, the residue was purified by rotatory chromatography (hexanes/EtOAc, 40:60), yielding compound **23** (47.8 mg, 65%) as a syrup. [α]_D_: −37 (c 0.37, CHCl_3_). IR (CHCl_3_) ν_max_ 3435, 1721, 1702, 1518, 1424, 1213, 1043 cm^–1^. ^1^H NMR (500 MHz, CD_3_CN, 70 °C) δ_H_ 6.87 (1H, d, *J* = 7.3 Hz), 4.84 (1H, d, *J* = 12.8 Hz), 4.42 (1H, q, *J* = 7.8 Hz), 4.29 (1H, dd, *J* = 7.6, 4.7 Hz), 3.91 (1H, d, *J* = 12.8 Hz), 3.67 (3H, s), 3.66 (3H, s), 3.64 (3H, s), 3.11 (1H, dd, *J* = 8.7, 5.8 Hz), 2.83 (1H, ddd, *J* = 12.1, 9.7, 3.5 Hz), 2.58 (1H, dt, *J* = 11.9, 5.9 Hz), 1.99–1.89 (1H, m), 1.83–1.78 (1H, m), 1.71–1.48 (6H, m), 1.21 (3H, s), 1.20 (3H, s), 0.94 (3H, d, *J* = 6.5 Hz), 0.93 (3H, d, *J* = 6.4 Hz), 0.92 (3H, d, *J* = 6.3 Hz), 0.90 (3H, d, *J* = 6.4 Hz). ^13^C{^1^H} NMR (125.7 MHz, CD_3_CN, 70 °C) δc 178.0 (C), 174.3 (C), 173.7 (C), 158.3 (C), 65.5 (CH), 59.1 (CH_2_), 57.8 (CH), 53.4 (CH_3_), 52.8 (CH_3_), 52.7 (CH_3_), 51.8 (CH), 48.4 (C), 47.6 (CH_2_), 41.8 (CH_2_), 39.9 (CH_2_), 26.1 (CH), 26.0 (CH), 24.8 (CH_2_), 24.2 (CH_3_), 23.8 (CH_3_), 23.43 (CH_3_), 23.42 (CH_3_), 22.8 (CH_3_), 22.1 (CH_3_). HRMS (ESI-TOF) calcd for C_24_H_43_N_3_O_7_Na [M + Na]^+^, 508.2999; found, 508.3001. Anal. calcd for C_24_H_43_N_3_O_7_: C, 59.36; H, 8.93; N, 8.65. Found: C, 59.50; H, 9.09; N, 8.36.

#### *N*^2^-(*tert*-Butoxycarbonyl)-*N*^5^-(benzyloxycarbonyl)-L-ornithyl-L-phenylalanine Methyl Ester (**24**)

HBTU (1.71 g, 4.50 mmol) and DIPEA (2.15 mL, 1.60 g, 12.27 mmol) were added to a solution of commercial Boc-Orn(Cbz)-OH (1.50 g, 4.09 mmol) and l-phenylalanine methyl ester hydrochloride (883 mg, 4.09 mmol) in dry CH_2_Cl_2_ (15 mL) at 0 °C. The reaction mixture was stirred at room temperature for 2 h, then poured into water, and washed with saturated aqueous NaHCO_3_ solution (3 × 20 mL) and 5% HCl (3 × 20 mL). The organic layer was dried and evaporated as usual, and the residue was purified by column chromatography (hexanes/EtOAc, 6:4), giving dipeptide **24** (1.82 mg, 84%) as a crystalline solid: mp 124–126 °C (from EtOAc/pentane); [α]_D_: −37 (c 0.37, CHCl_3_). IR (CHCl_3_) ν_max_ 3442, 1716, 1685, 1517, 1218, 1051 cm^–1^. ^1^H NMR (500 MHz, CD_3_CN, 70 °C) δ_H_ 7.38–7.20 (10H, m), 6.85 (1H, d, *J* = 6.7 Hz), 5.55–5.45 (1H, br b), 5.30–5.40 (1H, br b), 5.08 (1H, d, *J* = 12.9 Hz), 5.06 (1H, d, *J* = 13.0 Hz), 4.68 (1H, ddd, *J* = 7.8, 7.7, 5.8 Hz), 4.04–3.98 (1H, m), 3.66 (3H, s), 3.18–3.06 (3H, m), 3.01 (1H, dd, *J* = 14.0, 7.7 Hz), 1.74–1.66 (1H, m), 1.52–1.45 (3H, m), 1.42 (9H, s). ^13^C{^1^H} NMR (125.7 MHz, CD_3_CN, 70 °C) δc 173.4 (C), 173.1 (C), 158.0 (C), 156.9 (C), 138.9 (C), 138.2 (C), 130.6 (2 × CH), 129.8 (2 × CH), 129.7 (2 × CH), 129.1 (CH), 129.0 (2 × CH), 128.1 (CH), 80.5 (C), 67.3 (CH_2_), 55.7 (CH), 54.9 (CH), 53.0 (CH_3_), 41.5 (CH_2_), 38.8 (CH_2_), 30.8 (CH_2_), 29.0 (3 × CH_3_), 27.3 (CH_2_). HRMS (ESI-TOF/QTOF) calcd for C_28_H_37_N_3_O_7_Na [M + Na]^+^, 550.2529; found, 550.2549. Anal. calcd for C_28_H_37_N_3_O_7_: C, 63.74; H, 7.07; N, 7.96. Found: C, 63.52; H, 7.06; N, 7.58.

#### *N*^2^-(*tert*-Butoxycarbonyl)-*N*^5^-(benzyloxycarbonyl)-L-ornithyl-L-phenylanyl-L-leucine Methyl Ester (**25**)

To a solution of dipeptide **24** (1.82 g, 3.45 mmol) in MeOH (15 mL) at 0 °C was added 2 N KOH (7 mL). The reaction mixture was allowed to reach room temperature and stirred for 2 h. Then, it was acidified to pH 2–3 with 5% HCl, poured into water, and extracted with EtOAc. The organic layer was dried and evaporated as usual, affording the corresponding acid that was dissolved in CH_2_Cl_2_ and treated with **H-Leu-OMe** hydrochloride (627 mg, 3.45 mmol), HBTU (1.44 g, 3.80 mmol), and DIPEA (1.82 mL, 10.4 mmol). After stirring for 2 h, the reaction mixture was poured into water and washed with saturated aqueous NaHCO_3_ solution (3 × 20 mL) and 5% HCl (3 × 20 mL). The organic layer was dried and evaporated as usual, and the residue was purified by column chromatography (hexanes/EtOAc, 1:1), giving tripeptide **25** (1.74 mg, 79%) as a crystalline solid: mp 137–139 °C (from EtOAc/pentane); [α]_D_: −21 (c 0.47, CHCl_3_). IR (CHCl_3_) ν_max_ 3441, 3348, 1708, 1521, 1421, 1213, 1046 cm^–1^. ^1^H NMR (500 MHz, CD_3_CN, 70 °C) δ_H_ 7.39–7.21 (10H, m), 6.84 (1H, d, *J* = 7.4 Hz), 6.79 (1H, d, *J* = 7.3 Hz), 5.55–5.45 (1H, br b), 5.43–5.30 (1H, br b), 5.08 (2H, s), 4.60 (1H, dt, *J* = 8.1, 5.5 Hz), 4.43 (1H, dt, *J* = 8.4, 5.5 Hz), 3.97 (1H, dt, *J* = 7.7, 5.5 Hz), 3.66 (3H, s), 3.16–3.05 (3H, m), 2.96 (1H, dd, *J* = 14.1, 7.9 Hz), 1.71–1.44 (7H, m), 1.42 (9H, s), 0.92 (3H, d, *J* = 6.4 Hz), 0.90 (3H, d, *J* = 6.2 Hz). ^13^C{^1^H} NMR (125.7 MHz, CD_3_CN, 70 °C) δc 174.0 (C), 173.4 (C), 172.1 (C), 157.9 (C), 157.0 (C), 139.0 (C), 138.7 (C), 130.7 (2 × CH), 129.8 (2 × CH), 129.7 (2 × CH), 129.1 (CH), 129.0 (2 × CH), 127.9 (CH), 80.6 (C), 67.3 (CH_2_), 56.0 (CH), 55.4 (CH), 52.9 (CH_3_), 52.4 (CH), 42.0 (CH_2_), 41.5 (CH_2_), 38.9 (CH_2_), 30.6 (CH_2_), 29.0 (3 × CH_3_), 27.3 (CH_2_), 25.9 (CH), 23.3 (CH_3_), 22.4 (CH_3_). HRMS (ESI-TOF) calcd for C_34_H_48_N_4_O_8_Na [M + Na]^+^, 663.3370; found, 663.3381. Anal. calcd for C_34_H_48_N_4_O_8_: C, 63.73; H, 7.55; N, 8.74. Found: C, 63.34; H, 7.53; N, 8.49.

#### Methyl (2*S*-((3*S*,7*R*,8a*R*)-3-(3-[(Benzyloxycarbonyl)amino]propyl)-7-(1-methoxy-2-methyl-1-oxopropan-2-yl)-2-oxohexahydroimidazo[1,2-*c*]pyrimidin-1(5*H*)-yl)-3-phenylpropanoyl)-L-leucine (**26**) and Methyl [(2*S*)-[(4*S*)-4-(3-(Benzyloxycarbonylamino)prop-1-yl)-2-[4-methoxy-2*R*-(*N*-methoxycarbonyl-*N*-methoxymethylamino)-3,3-dimethyl-4-oxobutyl]-5-oxo-imidazolidin-1-yl]-3-phenylpropanoyl]-L-leucine (**27**)

A solution of peptide **25** (134.6 mg, 0.21 mmol) in a 1:1 mixture of TFA:DCM (1 mL) was stirred at 0° for 1 h. Then, the solvent was evaporated under vacuum and the residue was dissolved in methanol and added to a solution of aldehyde **5c** (49.5 mg, 0.15 mmol) and Et_3_N, which was later treated with NaBH_4_ according to the general reductive amination protocol. After work-up and solvent evaporation, the residue was purified by rotatory chromatography (hexanes/EtOAc, 40:60), yielding compounds **26** (41.1 mg, 36%) and **27** (35.3 mg, 30%) as colorless oils.

##### Compound **26**

[α]_D_: −26 (c 0.59, CHCl_3_). IR (CHCl_3_) ν_max_ 3445, 1712, 1696, 1518, 1444, 1213, 1045 cm^–1^. ^1^H NMR (400 MHz, CD_3_CN, 70 °C) δ_H_ 7.40–7.23 (10H, m), 6.97 (1H, d, *J* = 7.6 Hz), 5.58–5.44 (1H, br b), 5.10 (2H, s), 4.81 (1H, d, *J* = 11.0 Hz), 4.51–4.42 (2H, m), 4.38 (1H, td, *J* = 11.8, 2.5 Hz), 4.25 (1H, dd, *J* = 12.2, 6.6 Hz), 3.70 (3H, s), 3.67 (6H, s), 3.59 (1H, d, *J* = 11.0 Hz), 3.39–3.25 (2H, m), 3.22–3.18 (1H, m), 3.13–3.07 (2H, m), 1.83 (1H, ddd, *J* = 13.5, 6.7, 2.7 Hz), 1.74–1.34 (6H, m), 1.26–1.15 (2H, m), 1.12 (3H, s), 1.08 (3H, s), 0.98 (3H, d, *J* = 6.3 Hz), 0.96 (3H, d, *J* = 6.2 Hz). ^13^C{^1^H} NMR (100.6 MHz, CD_3_CN, 70 °C) δc 177.1 (C), 174.5 (C), 174.0 (C), 170.9 (C), 158.7 (C), 157.7 (C), 139.1 (C), 138.9 (C), 130.4 (2 × CH), 129.9 (2 × CH), 129.8 (2 × CH), 129.1 (CH), 129.0 (2 × CH), 128.1 (CH), 73.0 (CH), 67.2 (CH_2_), 62.5 (CH), 61.5 (CH_2_), 58.9 (CH), 58.0 (CH), 53.8 (CH_3_), 53.0 (CH_3_), 52.7 (CH_3_), 52.6 (CH), 48.2 (C), 42.13 (CH_2_), 42.06 (CH_2_), 34.5 (CH_2_), 29.3 (CH_2_), 27.8 (CH_2_), 26.6 (CH_2_), 26.1 (CH), 23.3 (CH_3_), 22.7 (CH_3_), 22.6 (CH_3_), 22.0 (CH_3_). HRMS (ESI-TOF) calcd for C_40_H_55_N_5_O_10_Na [M + Na]^+^, 788.3847; found, 788.3843; calcd for C_40_H_56_N_5_O_10_ [M + H]^+^, 766.4027; found, 766.4026.

##### Compound **27**

[α]_D_: +6 (c 0.34, CHCl_3_). IR (CHCl_3_) ν_max_ 3446, 1710, 1700, 1680, 1520, 1419, 1219, 1048 cm^–1^. ^1^H NMR (500 MHz, CD_3_CN, 70 °C) δ_H_ 7.65 (1H, d, *J* = 6.5 Hz), 7.39–7.20 (10H, m), 5.60–5.45 (1H, br b), 5.07 (2H, s), 4.60–4.40 (4H, m), 4.48–4.41 (1H, td, *J* = 8.4, 5.7 Hz), 3.98 (1H, br dd, *J* = 10.2, 5.7 Hz), 3.83–3.76 (1H, m), 3.67 (3H, s), 3.66 (3H, s), 3.60 (3H, s), 3.53 (1H, dd, *J* = 13.7, 10.4 Hz), 3.45 (1H, dd, *J* = 7.6, 4.6 Hz), 3.28 (1H, dd, *J* = 13.7, 5.7 Hz), 3.22 (3H, br s), 3.16 (2H, q, *J* = 6.7 Hz), 1.77–1.55 (7H, m), 1.53–1.45 (1H, m), 1.34 (2H, br t, *J* = 13.1, 12.9 Hz), 1.15 (3H, s), 1.13 (3H, s), 0.95 (3H, d, *J* = 6.6 Hz), 0.94 (3H, d, *J* = 6.5 Hz). ^13^C{^1^H} NMR (125.7 MHz, CD_3_CN, 70 °C) δc 177.7 (C), 177.4 (C), 174.2 (C), 171.7 (C), 158.7 (C), 157.7 (C), 139.3 (C), 139.0 (C), 130.5 (2 × CH), 129.8 (2 × CH), 129.7 (2 × CH), 129.1 (CH), 129.0 (2 × CH), 128.0 (CH), 74.6 (CH), 67.2 (CH_2_), 62.9 (CH), 60.1 (CH), 56.8 (CH_3_), 55.2 (CH), 53.7 (CH_3_), 53.0 (CH_3_), 52.7 (CH_3_), 52.4 (CH), 48.3 (C), 42.1 (CH_2_), 42.0 (CH_2_), 36.9 (CH_2_), 35.7 (CH_2_), 32.2 (CH_2_), 27.3 (CH_2_), 26.1 (CH), 24.6 (CH_3_), 23.5 (CH_3_), 23.4 (CH_3_), 22.5 (CH_3_). HRMS (ESI-TOF) calcd for C_41_H_59_N_5_O_11_Na [M + Na]^+^, 820.4109; found, 820.4106; calcd for C_40_H_56_N_5_O_10_ [M + H]^+^, 766.4027; found, 766.4023.

## Data Availability

The data underlying this study are available in the published article and its Supporting Information.

## Supporting Information Available

^1^H and ^13^C NMR of compounds **7–23**, COSY of **21**, and HSQC of compounds **17**, **18**, **21**, and **23**; ^1^H/^13^C NMR, COSY, HSQC, HMBC, and NOESY of “turn-inducing” compounds **26** and **27** and ^1^H/^13^C NMR of their precursors **24** and **25**; MM2 calculations of minimum-energy conformations of compounds **26** and **epi-26** and key angles for coupling constant calculations (PDF)
