# Supplementary material for: Conversion of Hydroxyproline “Doubly Customizable Units” to Hexahydropyrimidines: Access to Conformationally Constrained Peptides
Source: J Org Chem. 2023 Jul 10;88(14):9910–9. doi: 10.1021/acs.joc.3c00673 (PMC10367070; doi:10.1021/acs.joc.3c00673)
Supplement: Supplementary file 1 — jo3c00673_si_001.pdf [file jo3c00673_si_001.pdf]

Supporting Information

Conversion of Hydroxyproline "Doubly Customizable Units" to Hexahydropyrimidines: Access to Conformationally-Constrained Peptides.

Dacil Hernández,[a,]* *Marina Porras*[a] *and Alicia Boto*[a,]*

[a] Instituto de Productos Naturales y Agrobiología del CSIC, Avda. Astrofísico Fco. Sánchez, 3; 38206-La Laguna, Tenerife, SPAIN

alicia@ipna.csic.es; dacil@ipna.csic.es

Table of Contents

**Content**	**Page number**
^{1}H and ^{13}C NMR of compounds **7-23**, COSY of 21 and HSQC of compounds **17**, **18**, **21** and **23**	S02
^{1}H/^{13}C NMR, COSY, HSQC, HMBC and NOESY of "turn-inducing" compounds **26** and **27**, and ^{1}H/^{13}C NMR of their precursors **24** and **25**	S25
Theoretical dihedral angles of compounds **26** and **epi-26** for coupling constant calculations.	S39

Reproductions of NMR Spectra

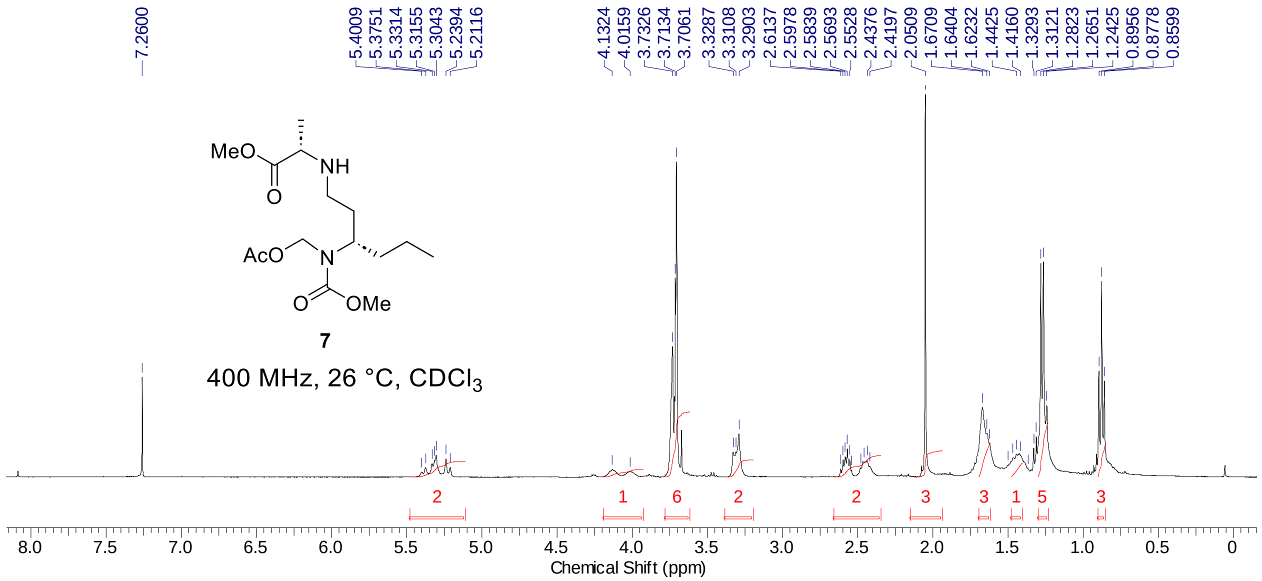

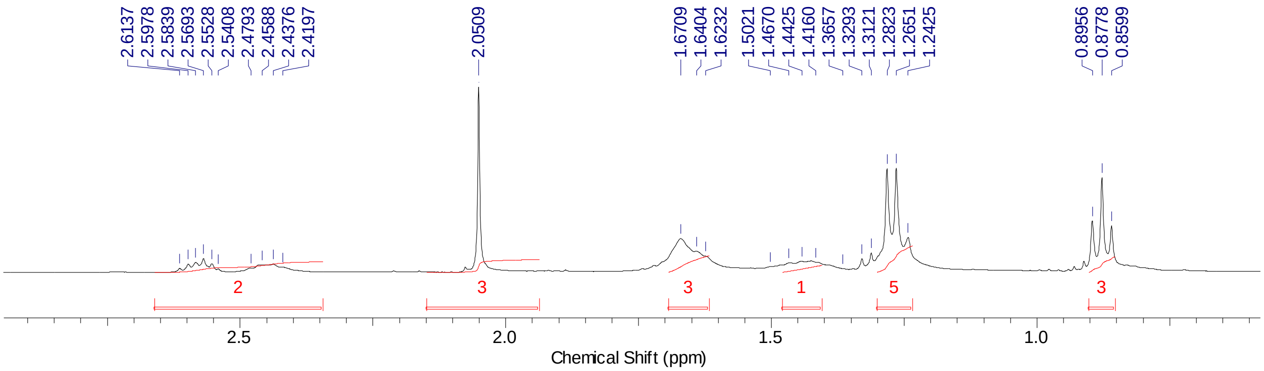

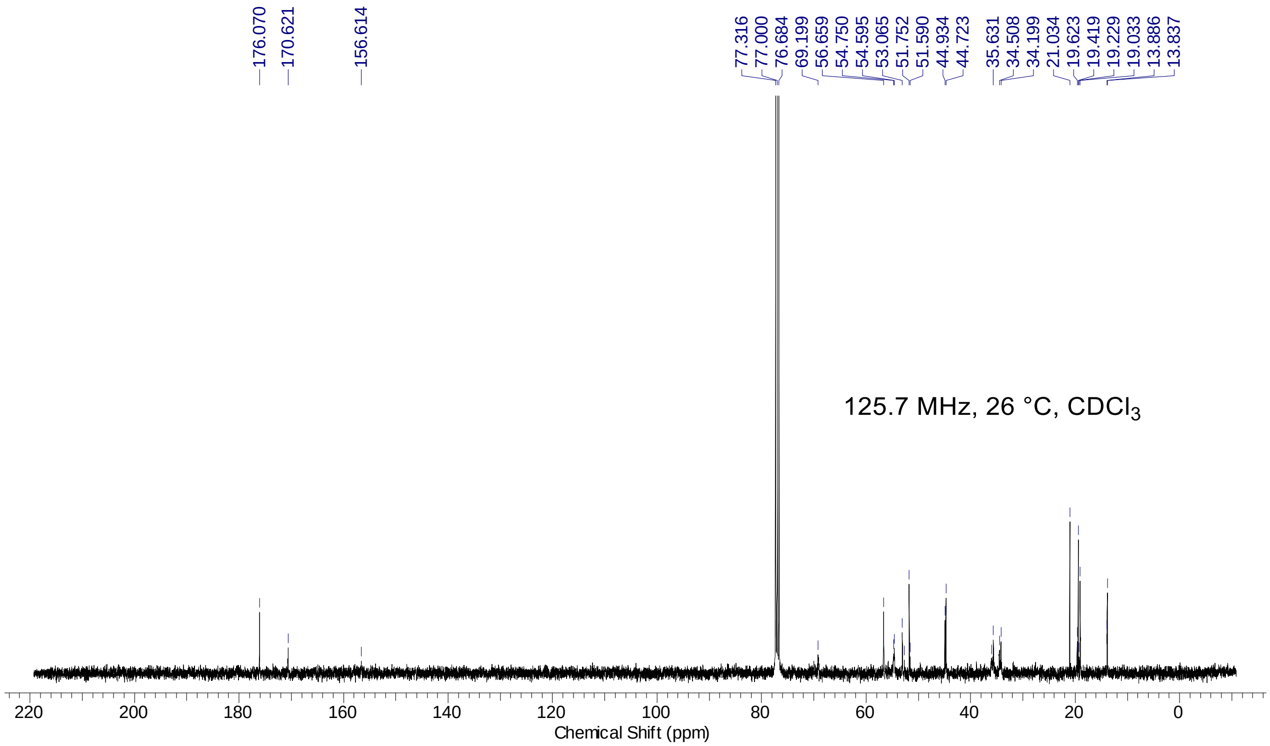

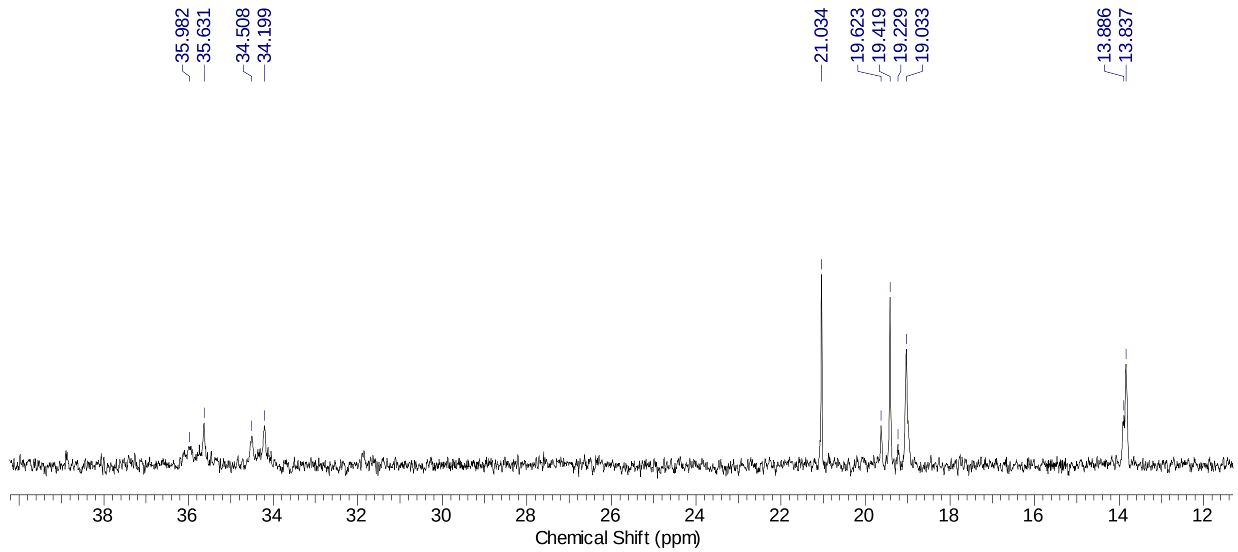

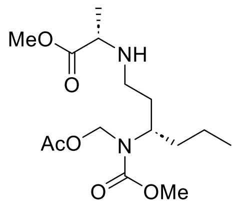

7

125.7 MHz, 26 °C, $CDCl_3$

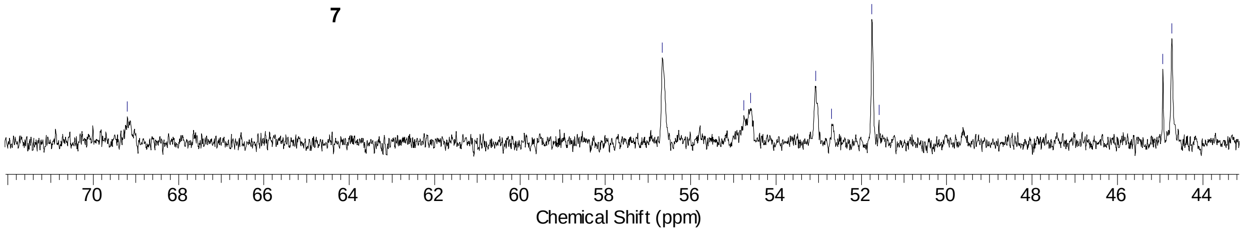

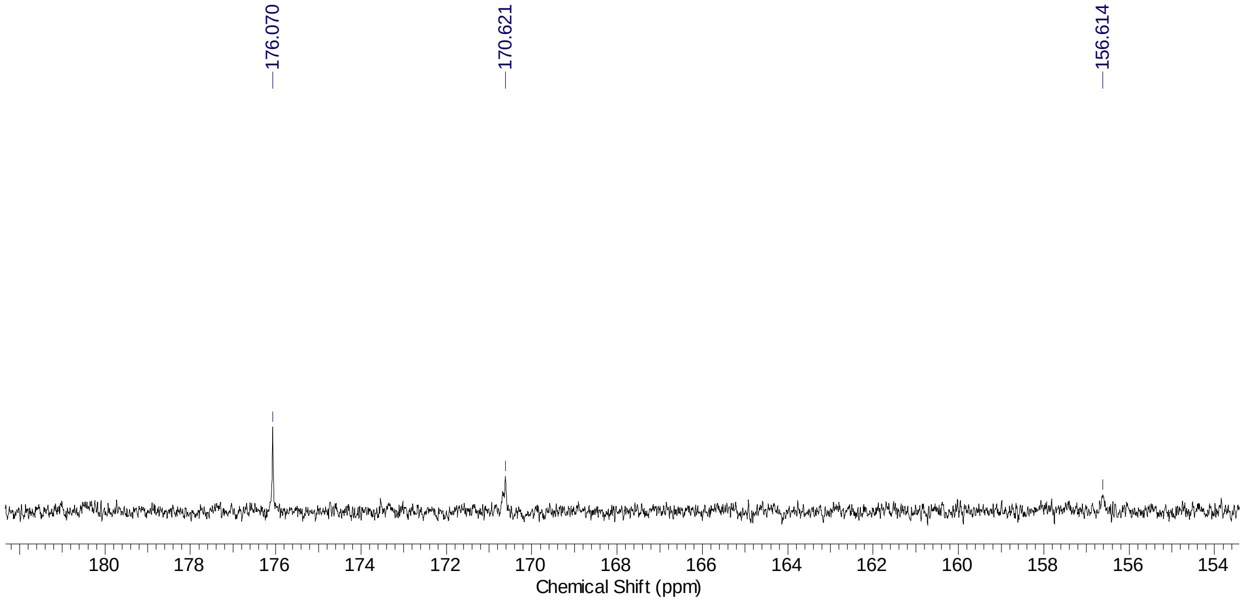

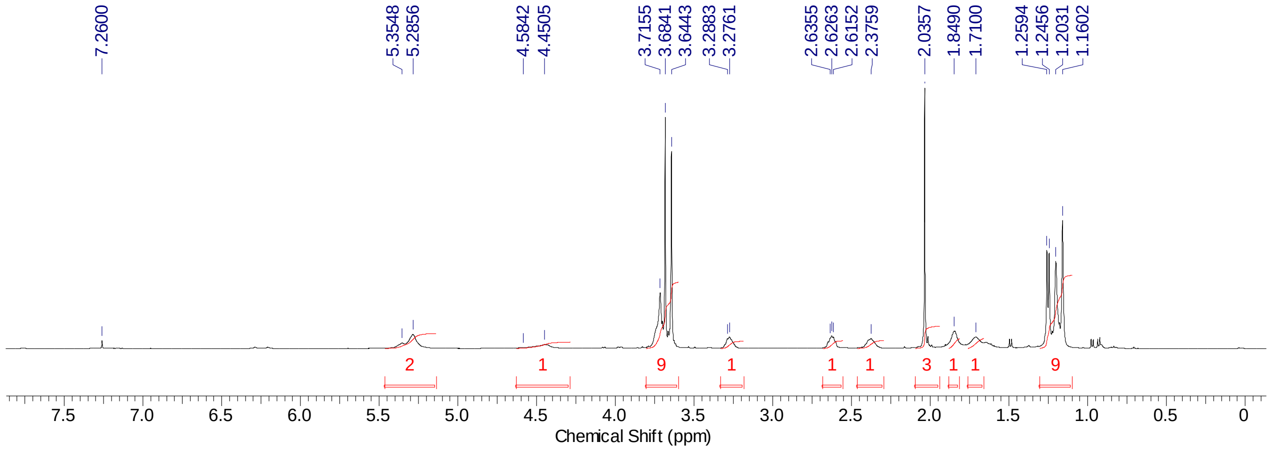
7.2600
5.3548
5.2856
4.5842
4.4505
3.7155
3.6841
3.6443
3.2883
3.2761
2.6355
2.6263
2.6152
2.3759
2.0357
1.8490
1.7100
1.2594
1.2456
1.2031
1.1602
2 1 9 1 1 1 3 1 1 9
Chemical Shift (ppm)

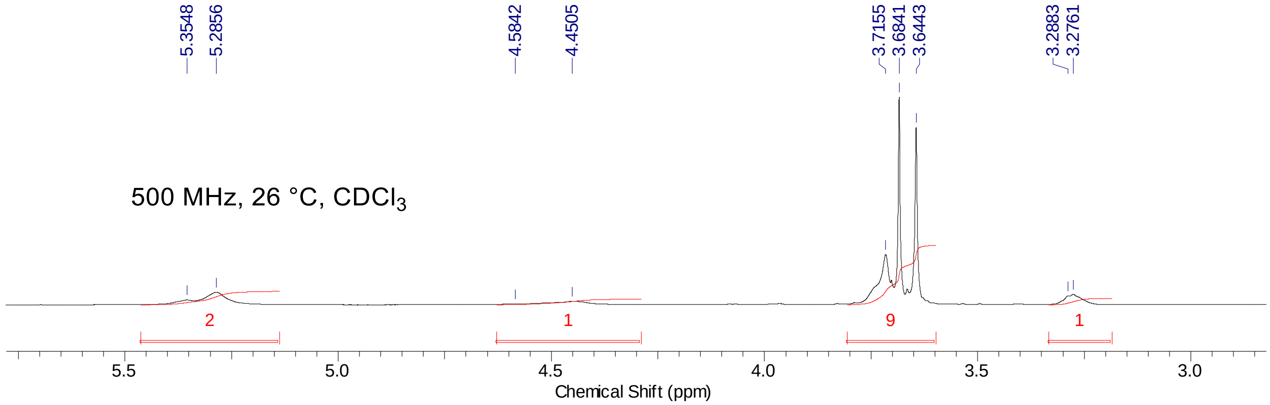
5.3548
5.2856
4.5842
4.4505
3.7155
3.6841
3.6443
3.2883
3.2761
500 MHz, 26 °C, CDCl3
2 1 9 1
Chemical Shift (ppm)

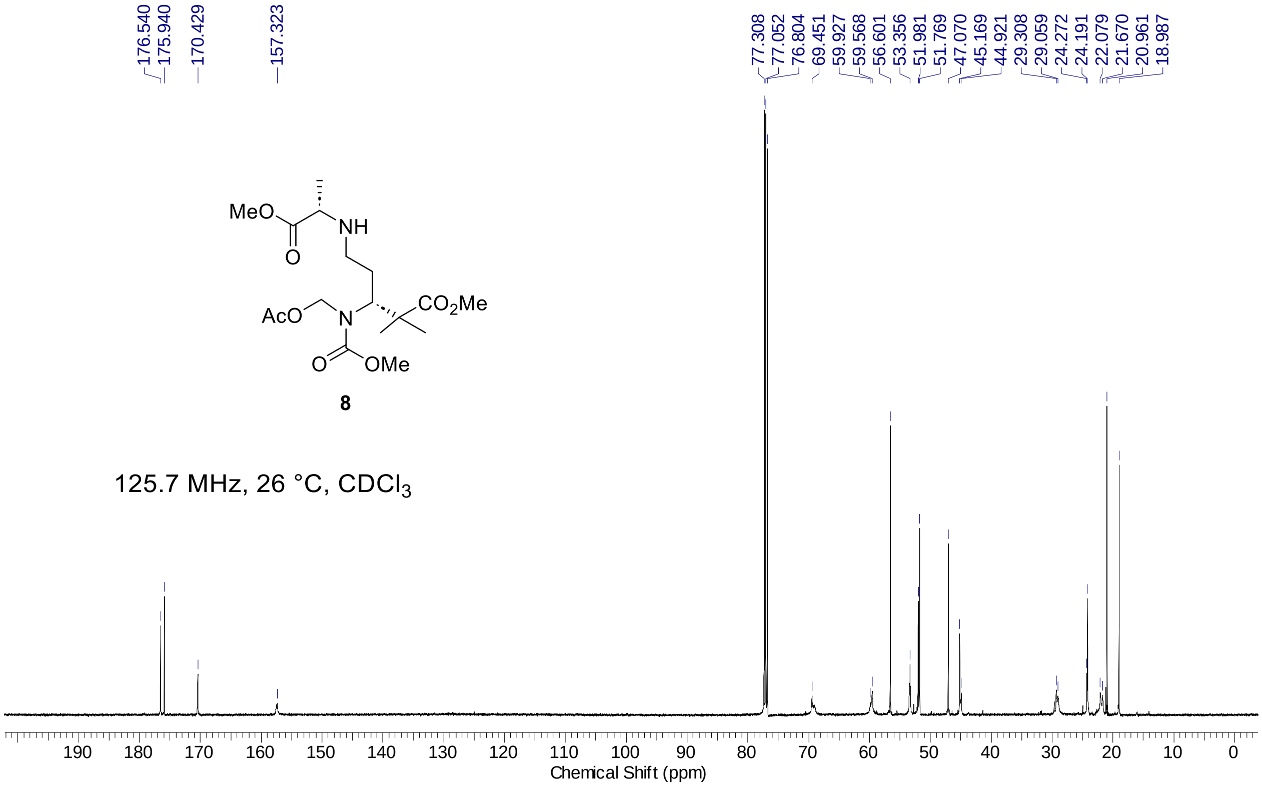
MeO
O
NH
AcO
N
CO2Me
O
OMe
8
176.540
175.940
170.429
157.323
77.308
77.052
76.804
69.451
59.927
59.568
56.601
53.356
51.981
51.769
47.070
45.169
44.921
29.308
29.059
24.272
24.191
22.079
21.670
20.961
18.987
125.7 MHz, 26 °C, CDCl3
Chemical Shift (ppm)

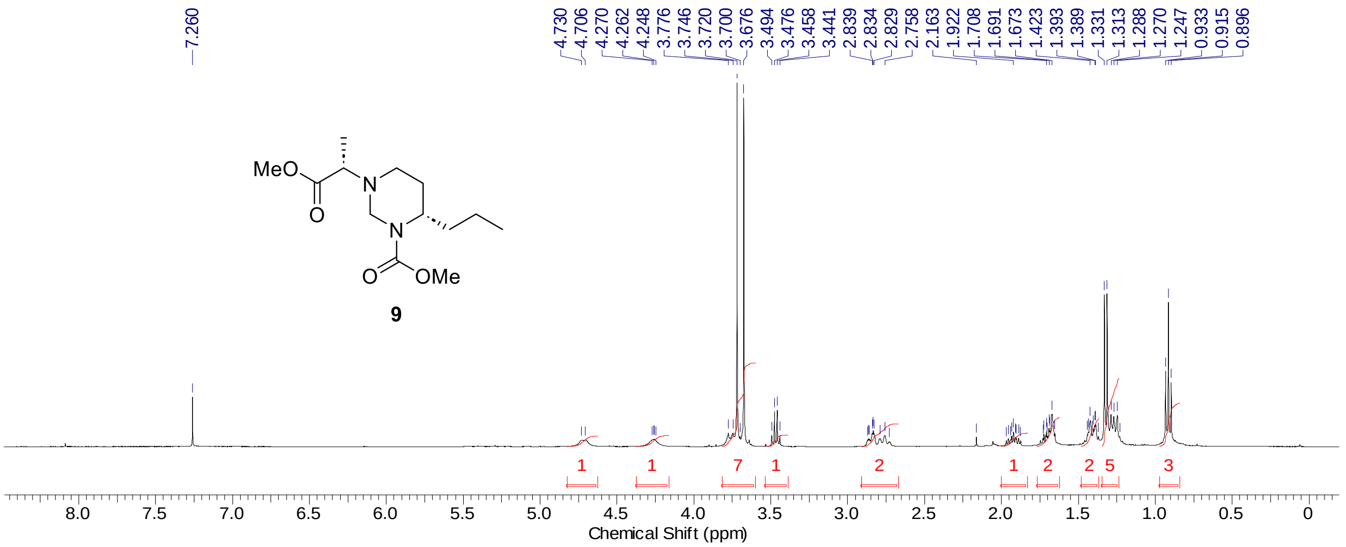
7.260
4.730 4.706 4.270 4.262 4.248 3.776 3.746 3.720 3.700 3.676 3.494 3.476 3.458 3.441 2.839 2.834 2.829 2.758 2.163 1.922 1.708 1.691 1.673 1.423 1.393 1.389 1.331 1.313 1.288 1.270 1.247 0.933 0.915 0.896
MeO
N
N
O
O
OMe
9
1 1 7 1 2 1 2 2 5 3
Chemical Shift (ppm)

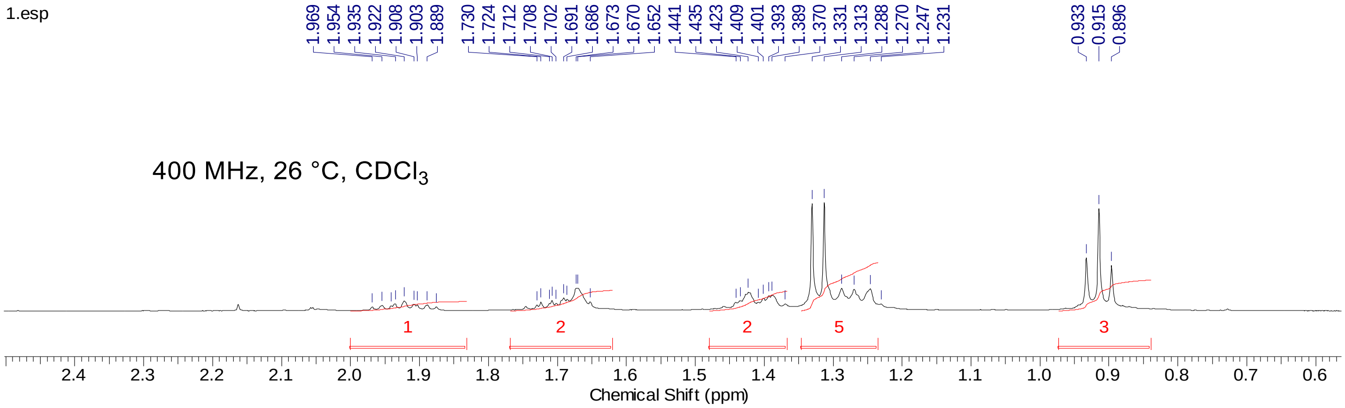
1.esp
1.969 1.954 1.935 1.922 1.908 1.903 1.889 1.730 1.724 1.712 1.708 1.702 1.691 1.686 1.673 1.670 1.652 1.441 1.435 1.423 1.409 1.401 1.393 1.389 1.370 1.331 1.313 1.288 1.270 1.247 1.231
0.933 0.915 0.896
400 MHz, 26 °C, CDCl3
1 2 2 5 3
Chemical Shift (ppm)

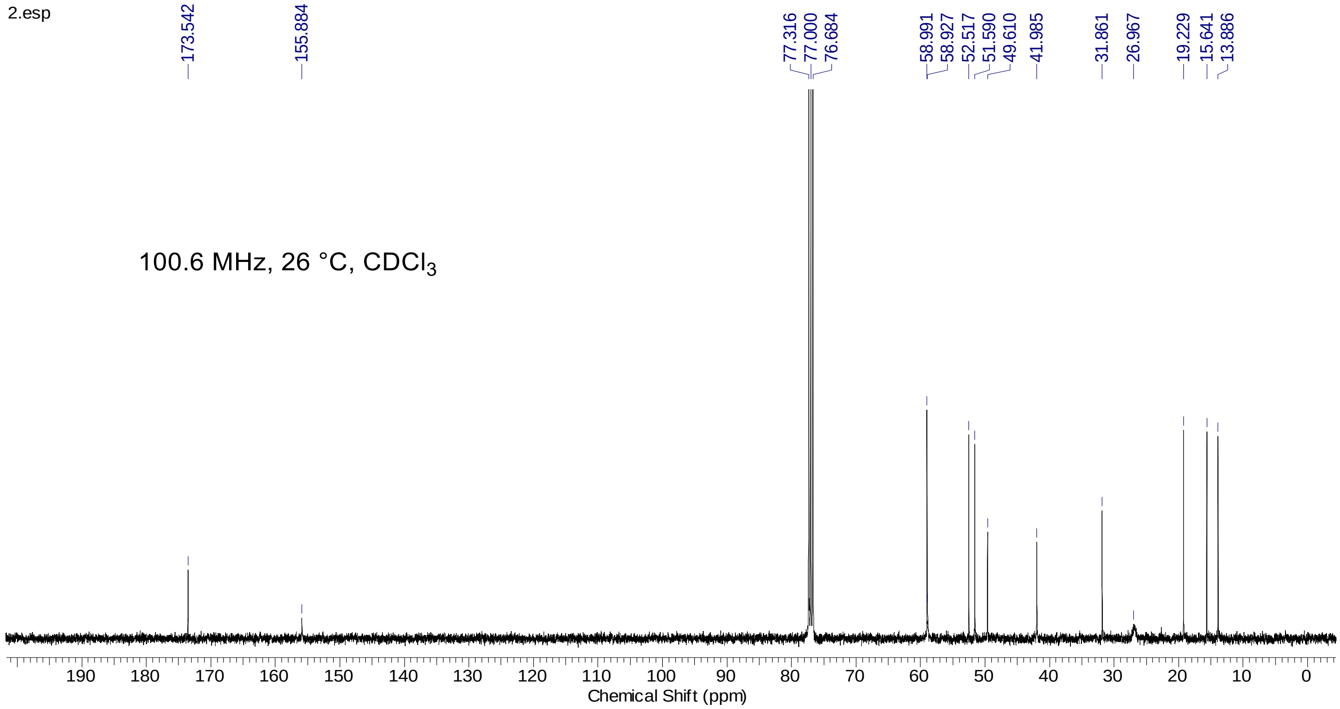
2.esp
173.542
155.884
77.316 77.000 76.684
58.991 58.927 52.517 51.590 49.610 41.985
31.861 26.967
19.229 15.641 13.886
100.6 MHz, 26 °C, CDCl3
Chemical Shift (ppm)

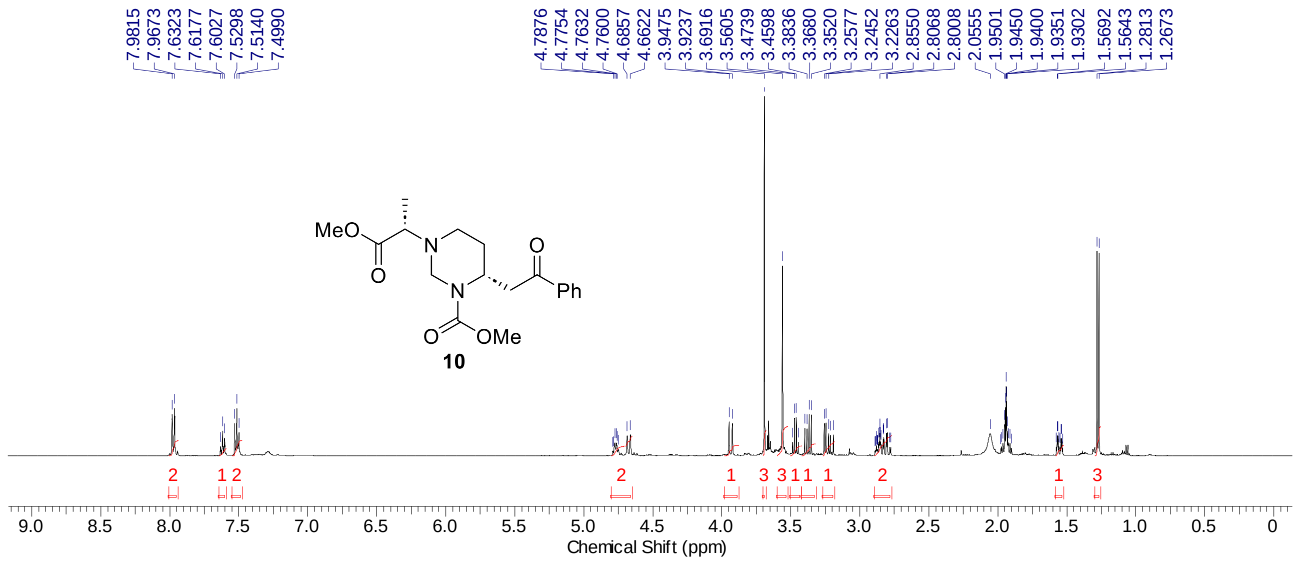
7.9815 7.9673 7.6323 7.6177 7.6027 7.5298 7.5140 7.4990
4.7876 4.7754 4.7632 4.7600 4.6857 4.6622 3.9475 3.9237 3.6916 3.5605 3.4739 3.4598 3.3836 3.3680 3.3520 3.2577 3.2452 3.2263 2.8550 2.8068 2.8008 2.0555 1.9501 1.9450 1.9400 1.9351 1.9302 1.5692 1.5643 1.2813 1.2673
MeO
O
N
N
O
Ph
O
OMe
10
2 1 2 2 1 3 3 1 1 1 2 1 3
9.0 8.5 8.0 7.5 7.0 6.5 6.0 5.5 5.0 4.5 4.0 3.5 3.0 2.5 2.0 1.5 1.0 0.5 0
Chemical Shift (ppm)

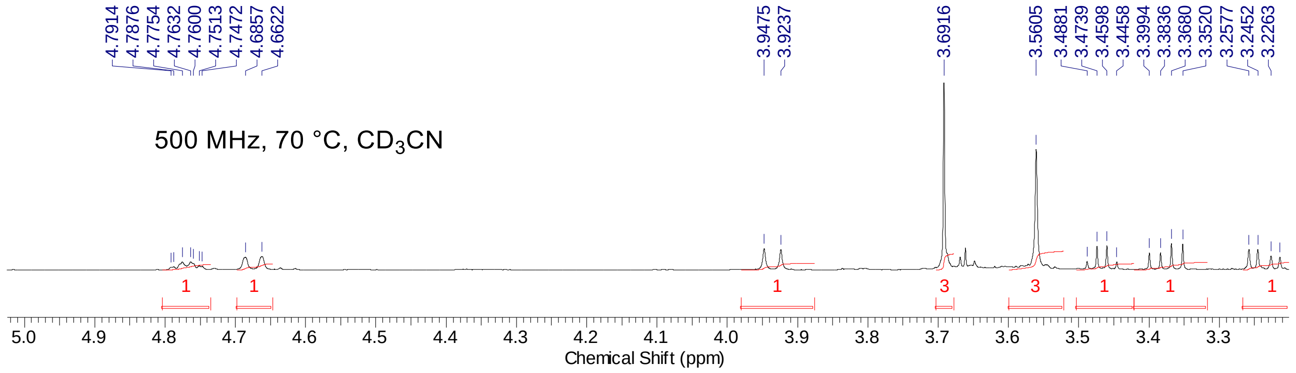
4.7914 4.7876 4.7754 4.7632 4.7600 4.7513 4.7472 4.6857 4.6622
3.9475 3.9237
3.6916
3.5605 3.4881 3.4739 3.4598 3.4458 3.3994 3.3836 3.3680 3.3520 3.2577 3.2452 3.2263
500 MHz, 70 °C, CD3CN
1 1 1 3 3 1 1 1
5.0 4.9 4.8 4.7 4.6 4.5 4.4 4.3 4.2 4.1 4.0 3.9 3.8 3.7 3.6 3.5 3.4 3.3
Chemical Shift (ppm)

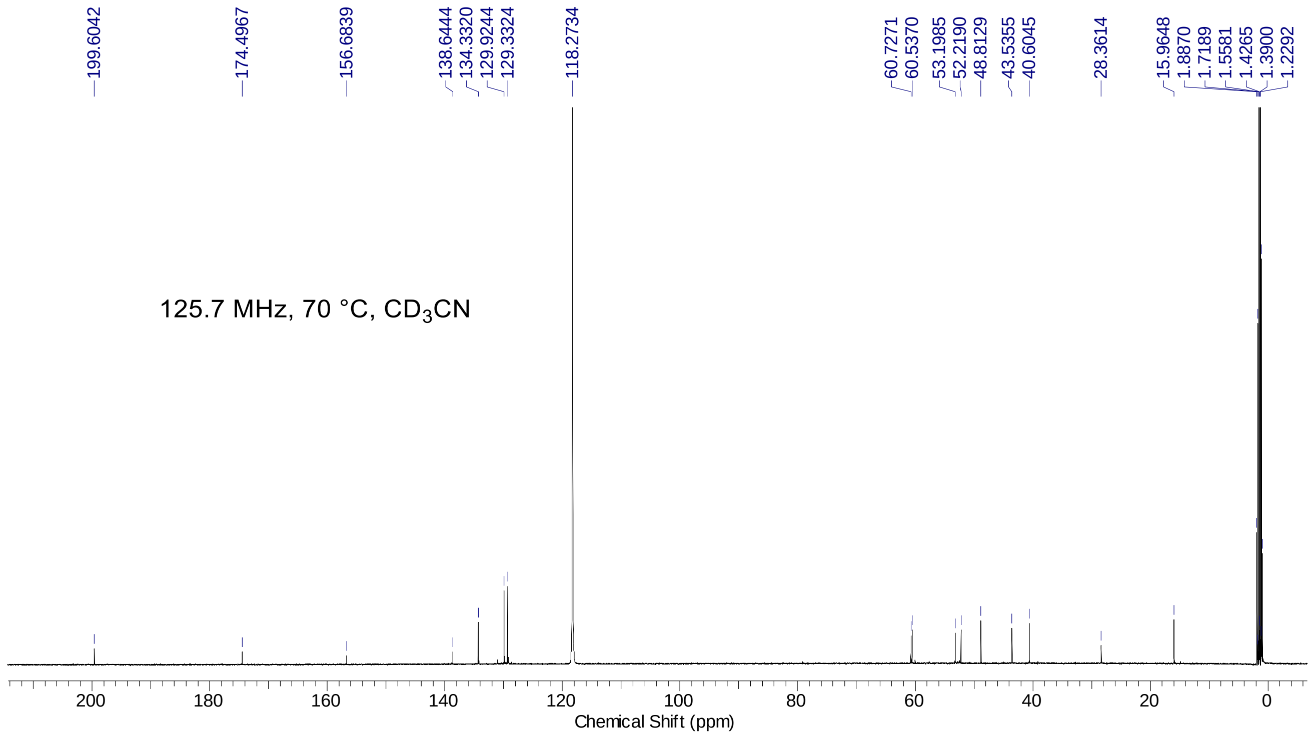
199.6042
174.4967
156.6839
138.6444 134.3320 129.9244 129.3324
118.2734
60.7271 60.5370 53.1985 52.2190 48.8129 43.5355 40.6045
28.3614
15.9648 1.8870 1.7189 1.5581 1.4265 1.3900 1.2292
125.7 MHz, 70 °C, CD3CN
200 180 160 140 120 100 80 60 40 20 0
Chemical Shift (ppm)

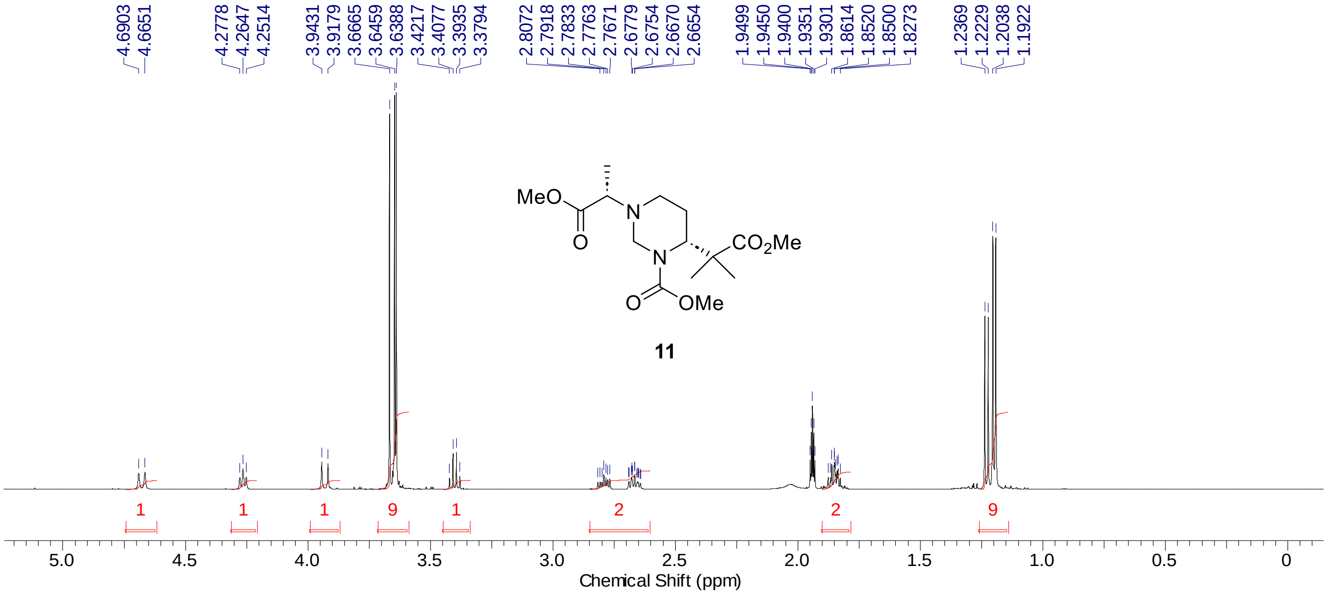
4.6903
4.6651
4.2778
4.2647
4.2514
3.9431
3.9179
3.6665
3.6459
3.6388
3.4217
3.4077
3.3935
3.3794
2.8072
2.7918
2.7833
2.7763
2.7671
2.6779
2.6754
2.6670
2.6654
1.9499
1.9450
1.9400
1.9351
1.9301
1.8614
1.8520
1.8500
1.8273
1.2369
1.2229
1.2038
1.1922
MeO
O
N
N
CO_2Me
O
OMe
11
1
1
1
9
1
2
2
9
Chemical Shift (ppm)

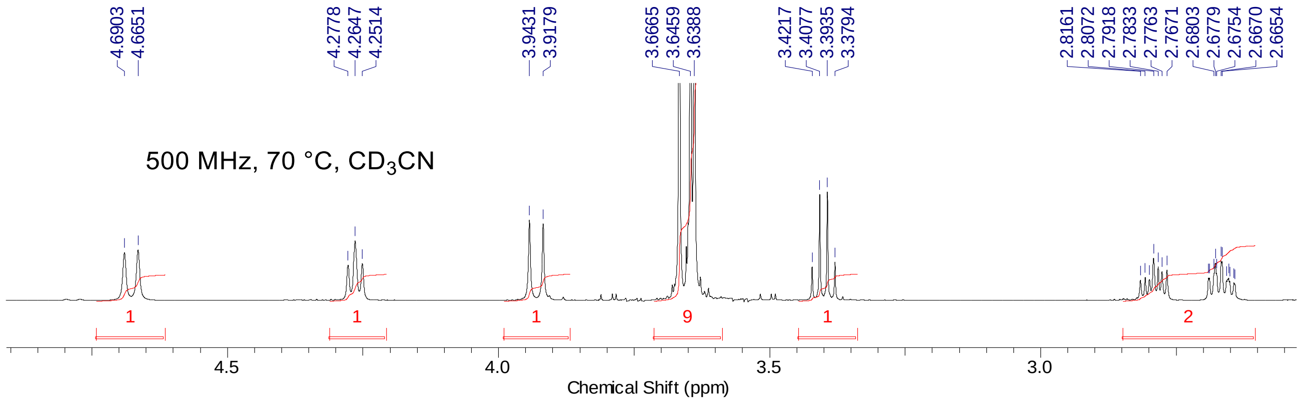
4.6903
4.6651
4.2778
4.2647
4.2514
3.9431
3.9179
3.6665
3.6459
3.6388
3.4217
3.4077
3.3935
3.3794
2.8161
2.8072
2.7918
2.7833
2.7763
2.7671
2.6803
2.6779
2.6754
2.6670
2.6654
500 MHz, 70 °C, CD_3CN
1
1
1
9
1
2
Chemical Shift (ppm)

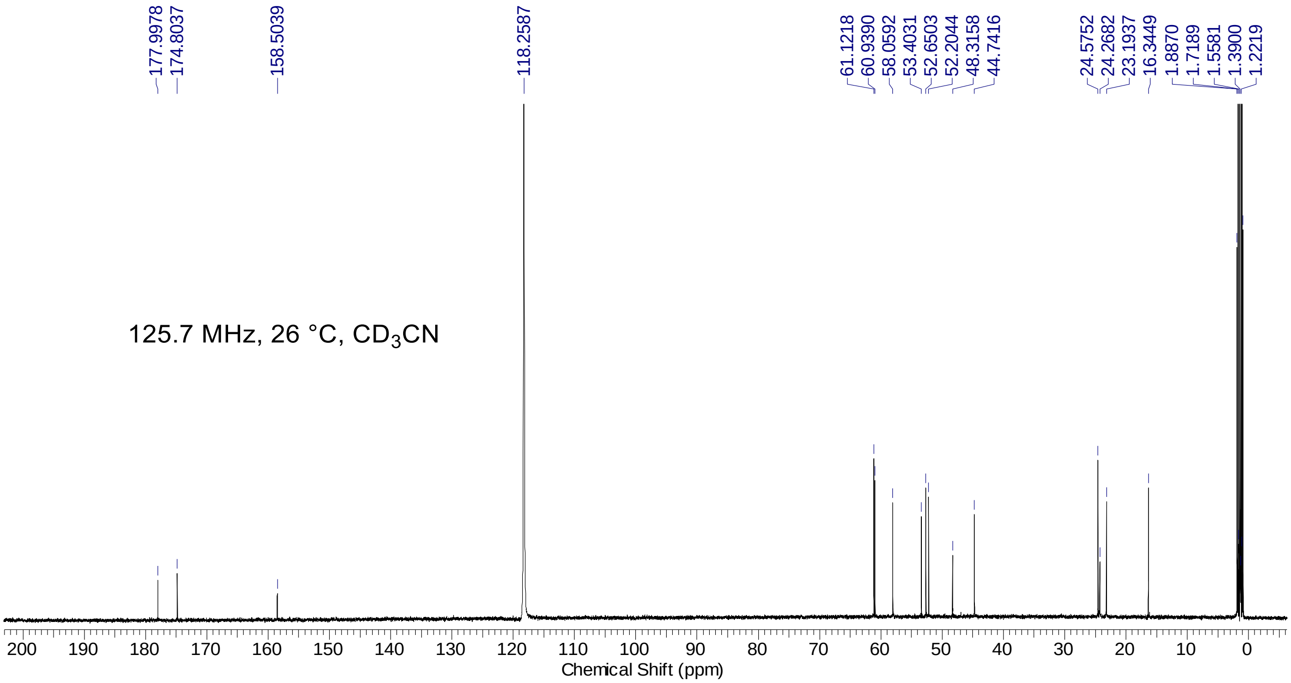
177.9978
174.8037
158.5039
118.2587
61.1218
60.9390
58.0592
53.4031
52.6503
52.2044
48.3158
44.7416
24.5752
24.2682
23.1937
16.3449
1.8870
1.7189
1.5581
1.3900
1.2219
125.7 MHz, 26 °C, CD_3CN
Chemical Shift (ppm)

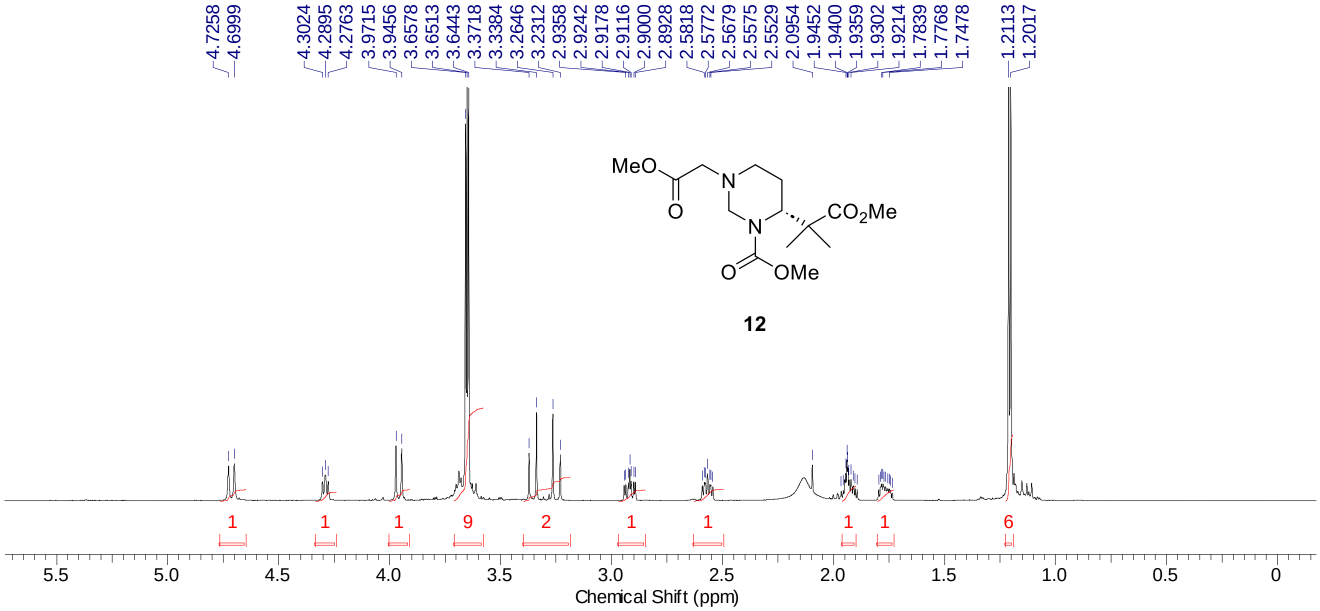
4.7258
4.6999
4.3024
4.2895
4.2763
3.9715
3.9456
3.6578
3.6513
3.6443
3.3718
3.3384
3.2646
3.2312
2.9358
2.9242
2.9178
2.9116
2.9000
2.8928
2.5818
2.5772
2.5679
2.5575
2.5529
2.0954
1.9452
1.9400
1.9359
1.9302
1.9214
1.7839
1.7768
1.7478
1.2113
1.2017
MeO
N
O
N
CO2Me
O
OMe
12
1
1
1
9
2
1
1
1
1
6
5.5 5.0 4.5 4.0 3.5 3.0 2.5 2.0 1.5 1.0 0.5 0
Chemical Shift (ppm)

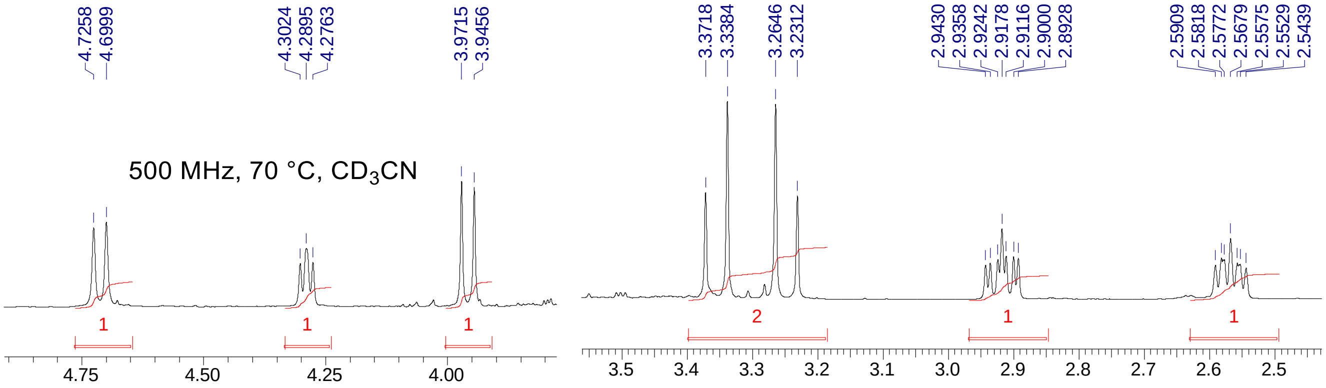
4.7258
4.6999
4.3024
4.2895
4.2763
3.9715
3.9456
500 MHz, 70 °C, CD3CN
1
1
1
4.75 4.50 4.25 4.00
3.3718
3.3384
3.2646
3.2312
2.9430
2.9358
2.9242
2.9178
2.9116
2.9000
2.8928
2.5909
2.5818
2.5772
2.5679
2.5575
2.5529
2.5439
2
1
1
3.5 3.4 3.3 3.2 3.1 3.0 2.9 2.8 2.7 2.6 2.5

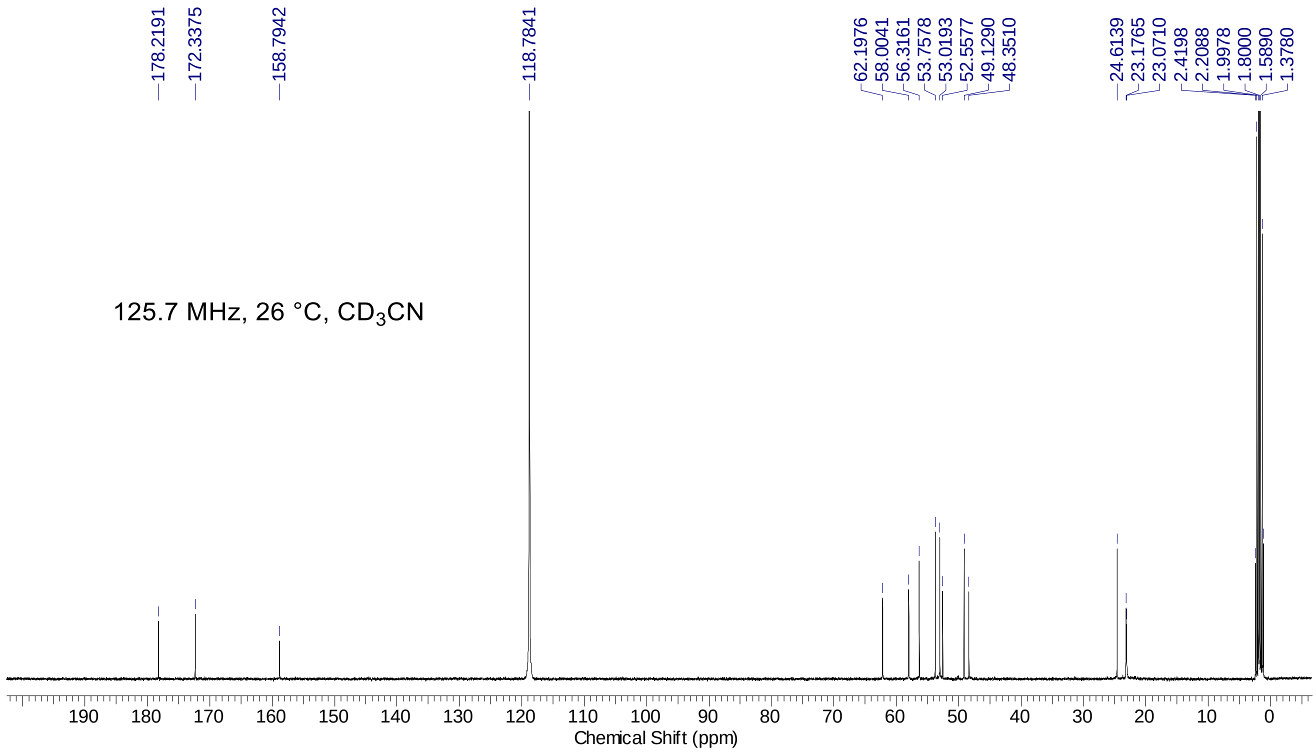
178.2191
172.3375
158.7942
118.7841
62.1976
58.0041
56.3161
53.7578
53.0193
52.5577
49.1290
48.3510
24.6139
23.1765
23.0710
2.4198
2.2088
1.9978
1.8000
1.5890
1.3780
125.7 MHz, 26 °C, CD3CN
190 180 170 160 150 140 130 120 110 100 90 80 70 60 50 40 30 20 10 0
Chemical Shift (ppm)

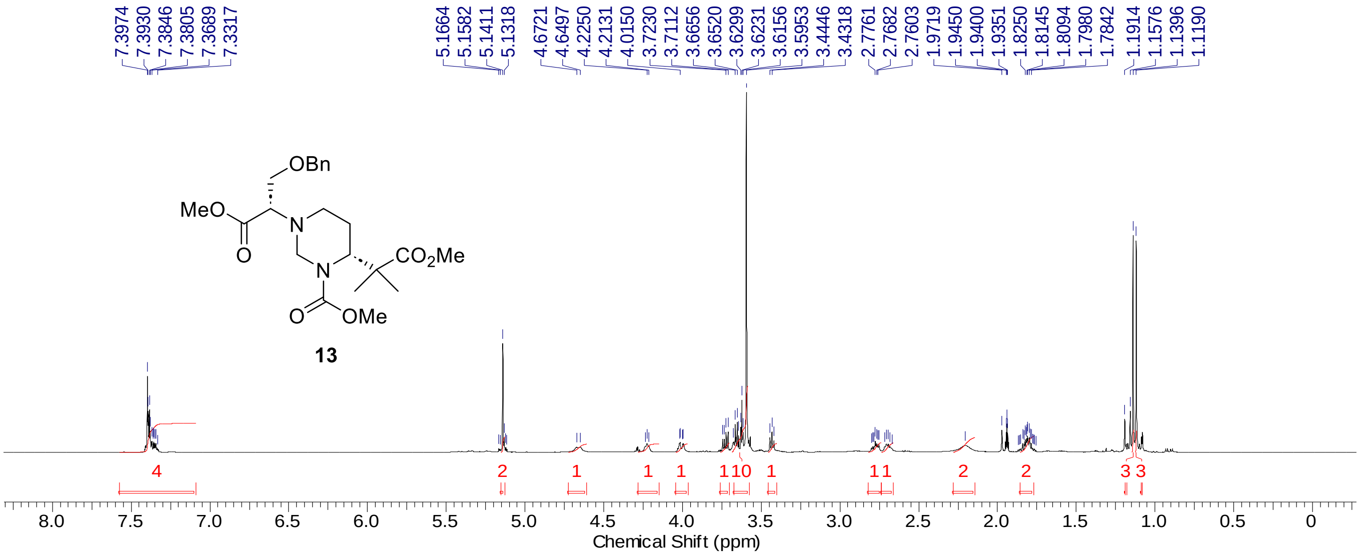
7.3974
7.3930
7.3846
7.3805
7.3689
7.3317
5.1664
5.1582
5.1411
5.1318
4.6721
4.6497
4.2250
4.2131
4.0150
3.7230
3.7112
3.6656
3.6520
3.6299
3.6231
3.6156
3.5953
3.4446
3.4318
2.7761
2.7682
2.7603
1.9719
1.9450
1.9400
1.9351
1.8250
1.8145
1.8094
1.7980
1.7842
1.1914
1.1576
1.1396
1.1190
OBn
MeO
O
N
N
CO_2Me
O
OMe
13
4
2
1
1
1
1
10
1
1
1
2
2
3
3
8.0
7.5
7.0
6.5
6.0
5.5
5.0
4.5
4.0
3.5
3.0
2.5
2.0
1.5
1.0
0.5
0
Chemical Shift (ppm)

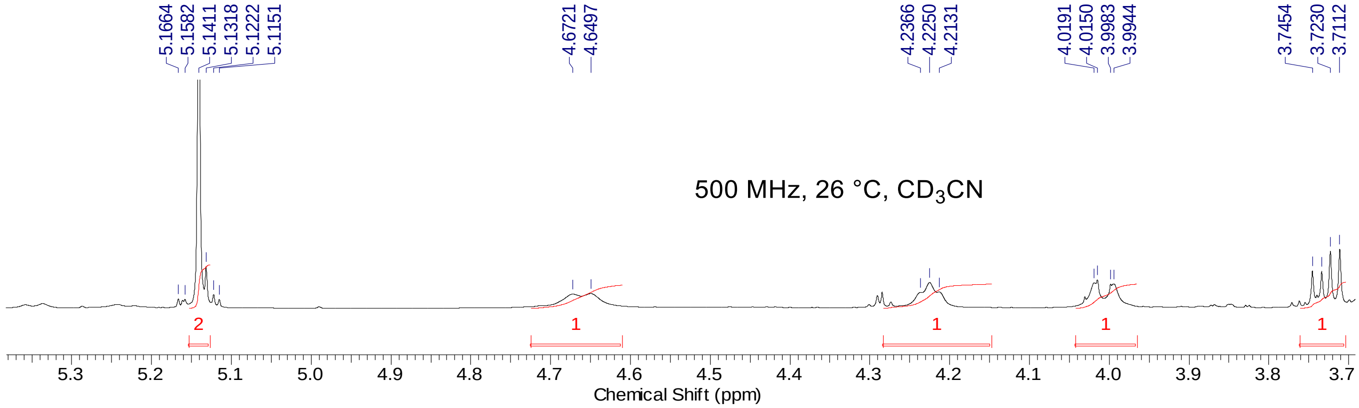
5.1664
5.1582
5.1411
5.1318
5.1222
5.1151
4.6721
4.6497
4.2366
4.2250
4.2131
4.0191
4.0150
3.9983
3.9944
3.7454
3.7230
3.7112
500 MHz, 26 °C, CD_3CN
2
1
1
1
1
5.3
5.2
5.1
5.0
4.9
4.8
4.7
4.6
4.5
4.4
4.3
4.2
4.1
4.0
3.9
3.8
3.7
Chemical Shift (ppm)

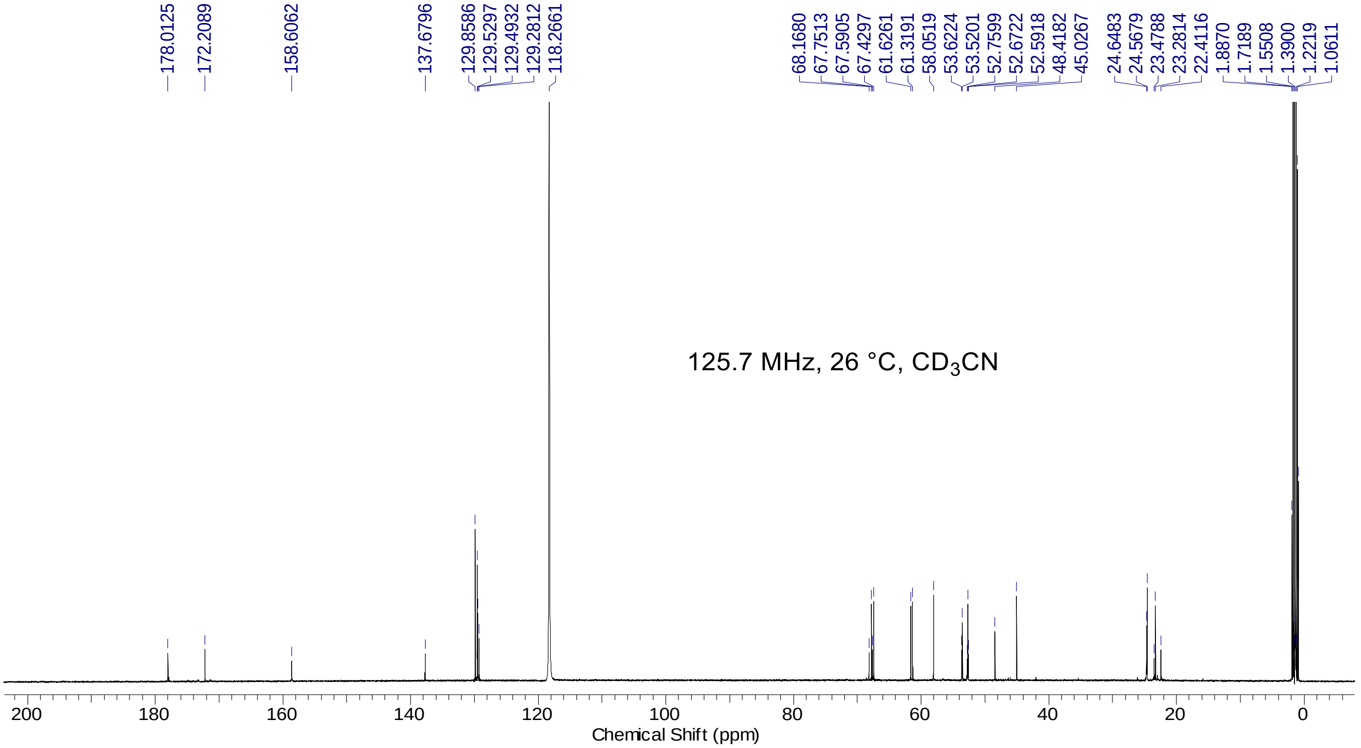
178.0125
172.2089
158.6062
137.6796
129.8586
129.5297
129.4932
129.2812
118.2661
68.1680
67.7513
67.5905
67.4297
61.6261
61.3191
58.0519
53.6224
53.5201
52.7599
52.6722
52.5918
48.4182
45.0267
24.6483
24.5679
23.4788
23.2814
22.4116
1.8870
1.7189
1.5508
1.3900
1.2219
1.0611
125.7 MHz, 26 °C, CD_3CN
200
180
160
140
120
100
80
60
40
20
0
Chemical Shift (ppm)

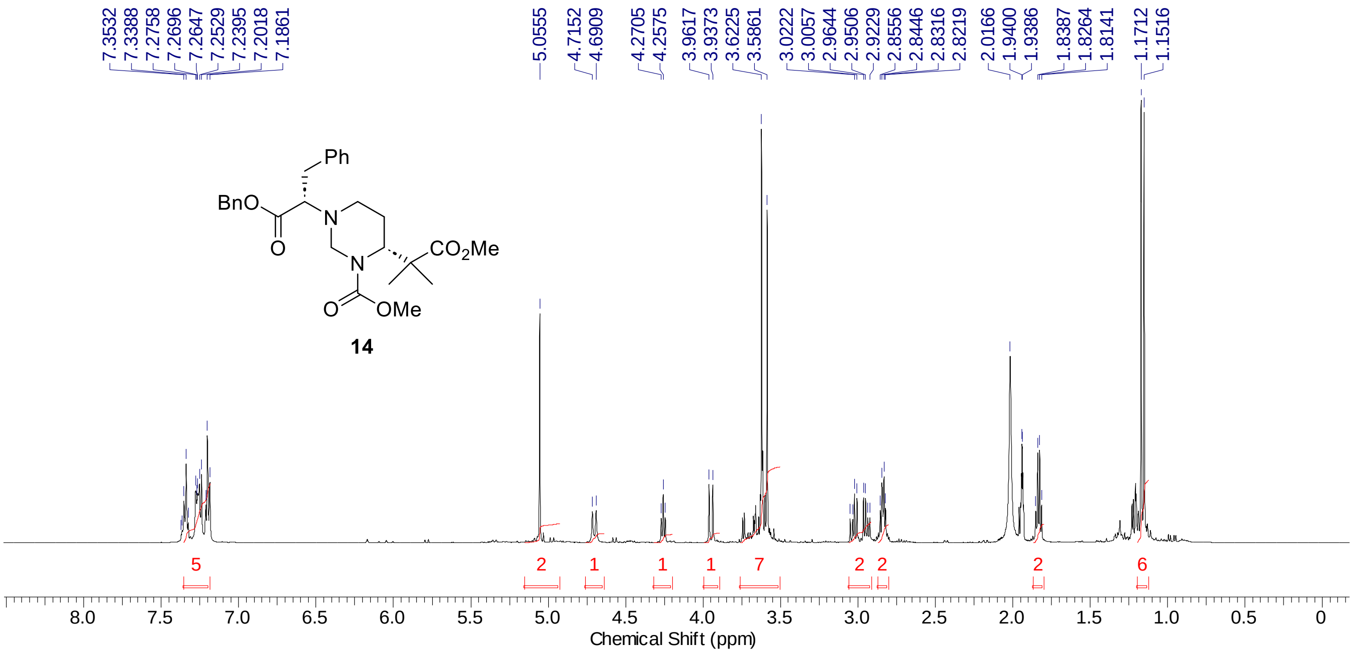
7.3532 7.3388 7.2758 7.2696 7.2647 7.2529 7.2395 7.2018 7.1861
5.0555
4.7152 4.6909
4.2705 4.2575
3.9617 3.9373
3.6225 3.5861
3.0222 3.0057 2.9644 2.9506 2.9229
2.8556 2.8446 2.8316 2.8219
2.0166 1.9400 1.9386
1.8387 1.8264 1.8141
1.1712 1.1516
Ph
BnO
N
N
O
CO_2Me
O
OMe
14
5 2 1 1 1 7 2 2 2 6
8.0 7.5 7.0 6.5 6.0 5.5 5.0 4.5 4.0 3.5 3.0 2.5 2.0 1.5 1.0 0.5 0
Chemical Shift (ppm)

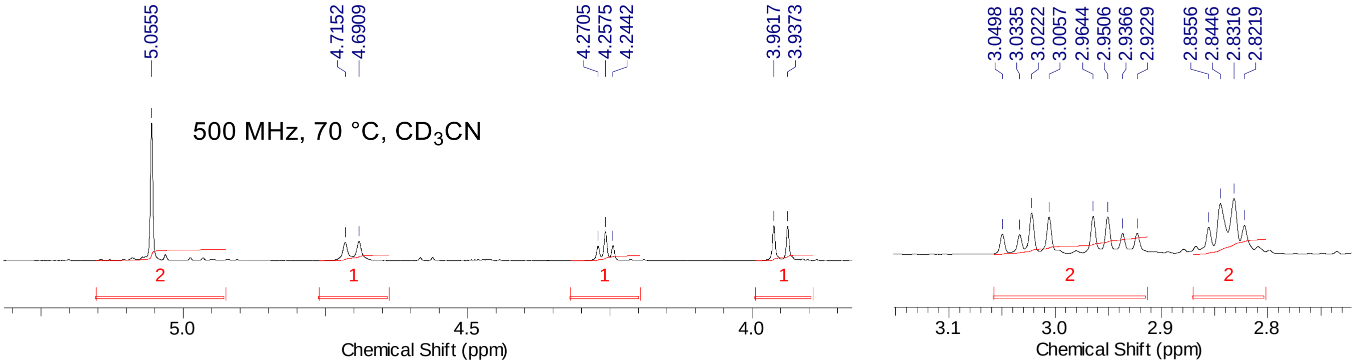
5.0555
4.7152 4.6909
4.2705 4.2575 4.2442
3.9617 3.9373
3.0498 3.0335 3.0222 3.0057 2.9644 2.9506 2.9366 2.9229
2.8556 2.8446 2.8316 2.8219
500 MHz, 70 °C, CD_3CN
2 1 1 1
5.0 4.5 4.0
Chemical Shift (ppm)
2 2
3.1 3.0 2.9 2.8
Chemical Shift (ppm)

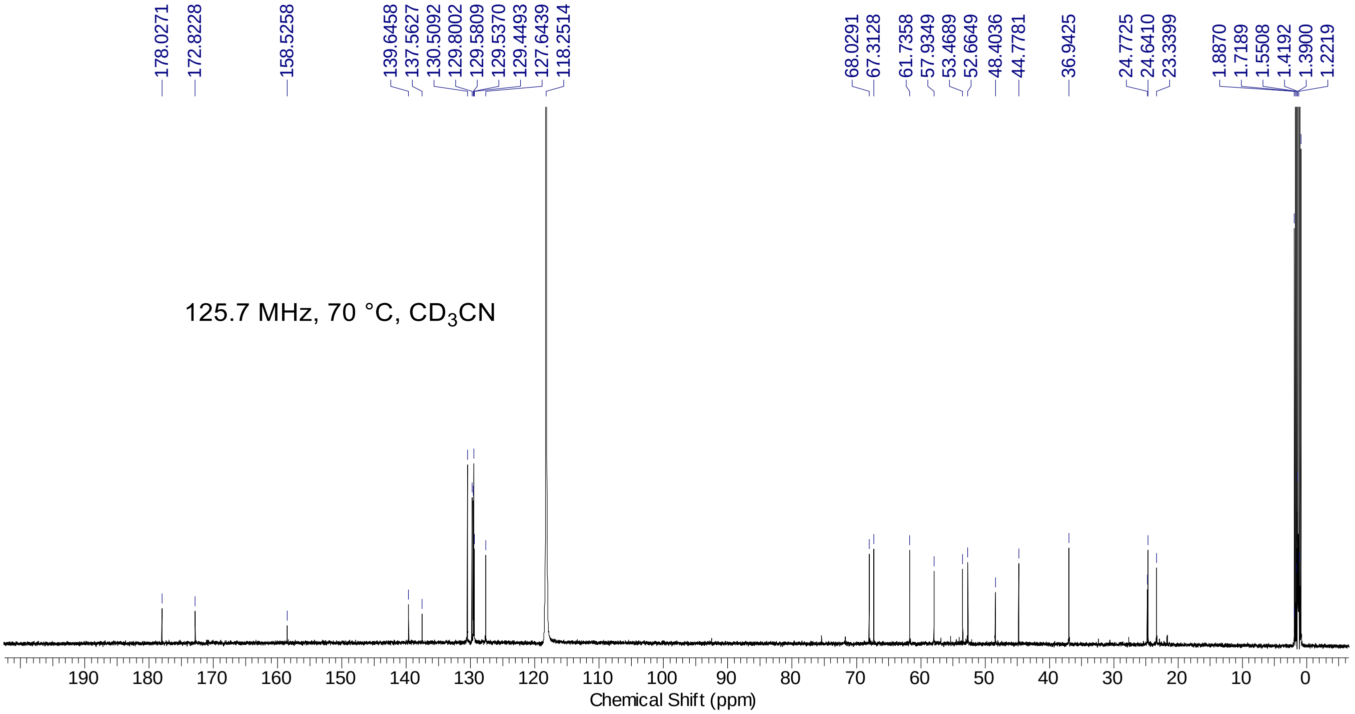
178.0271
172.8228
158.5258
139.6458 137.5627 130.5092 129.8002 129.5809 129.5370 129.4493 127.6439 118.2514
68.0291 67.3128
61.7358 57.9349 53.4689 52.6649
48.4036
44.7781
36.9425
24.7725 24.6410 23.3399
1.8870 1.7189 1.5508 1.4192 1.3900 1.2219
125.7 MHz, 70 °C, CD_3CN
190 180 170 160 150 140 130 120 110 100 90 80 70 60 50 40 30 20 10 0
Chemical Shift (ppm)

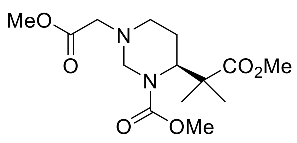

15

500 MHz, 70 °C, CD_3CN

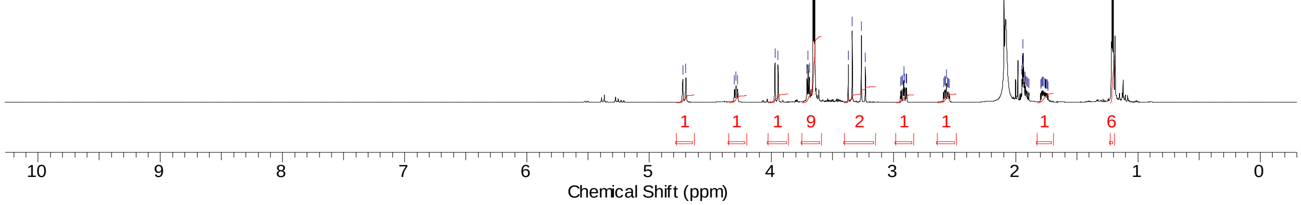

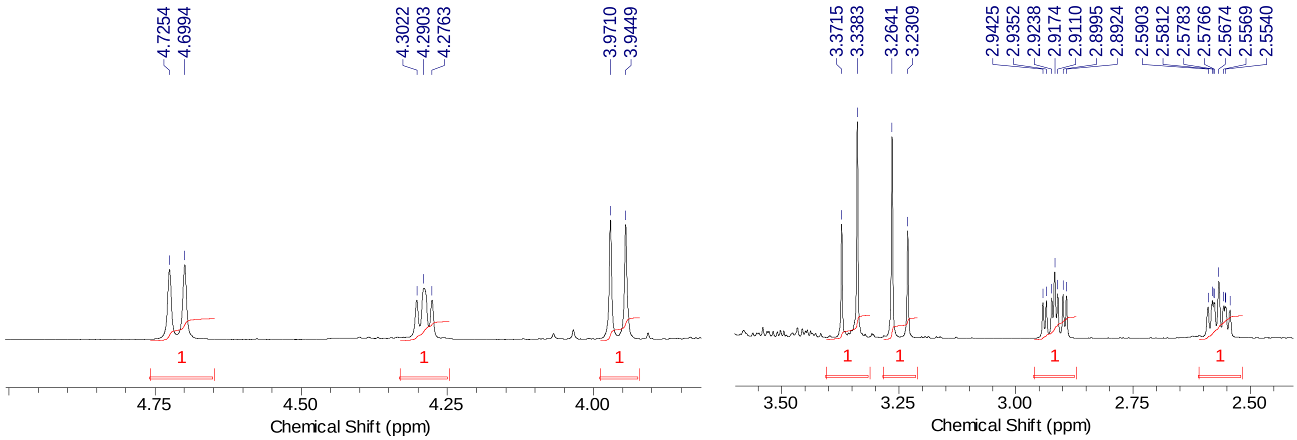

125.7 MHz, 26 °C, CD_3CN

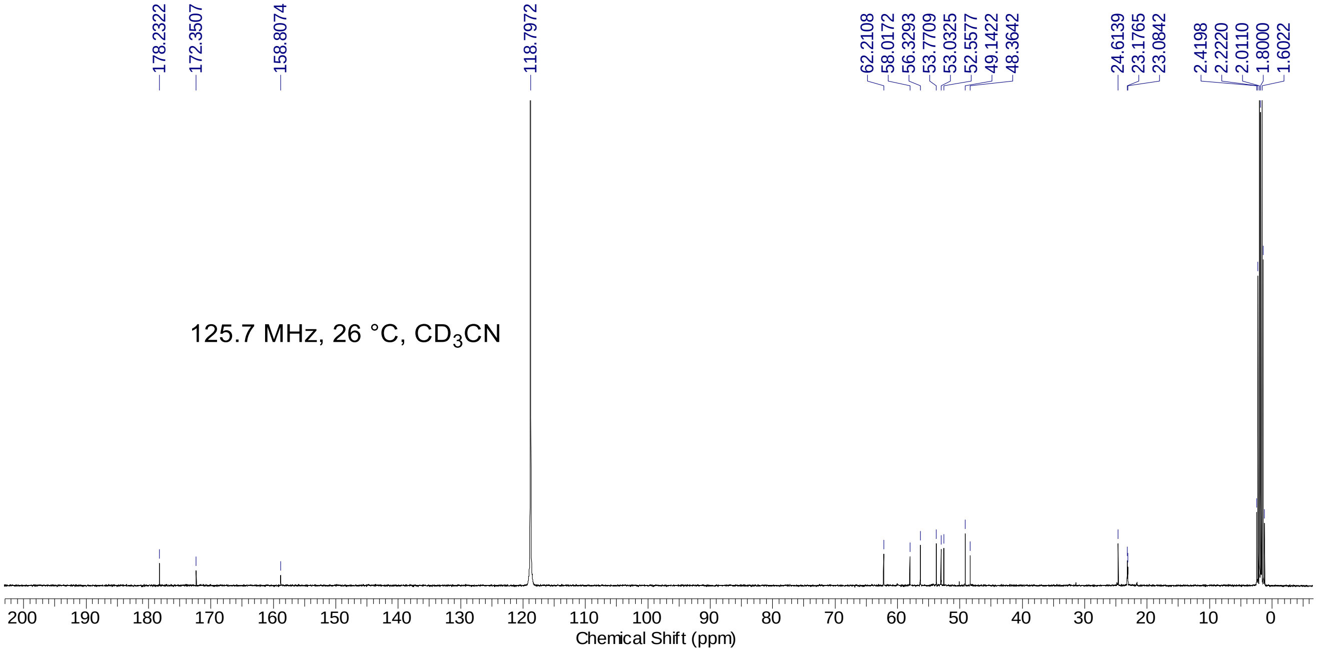

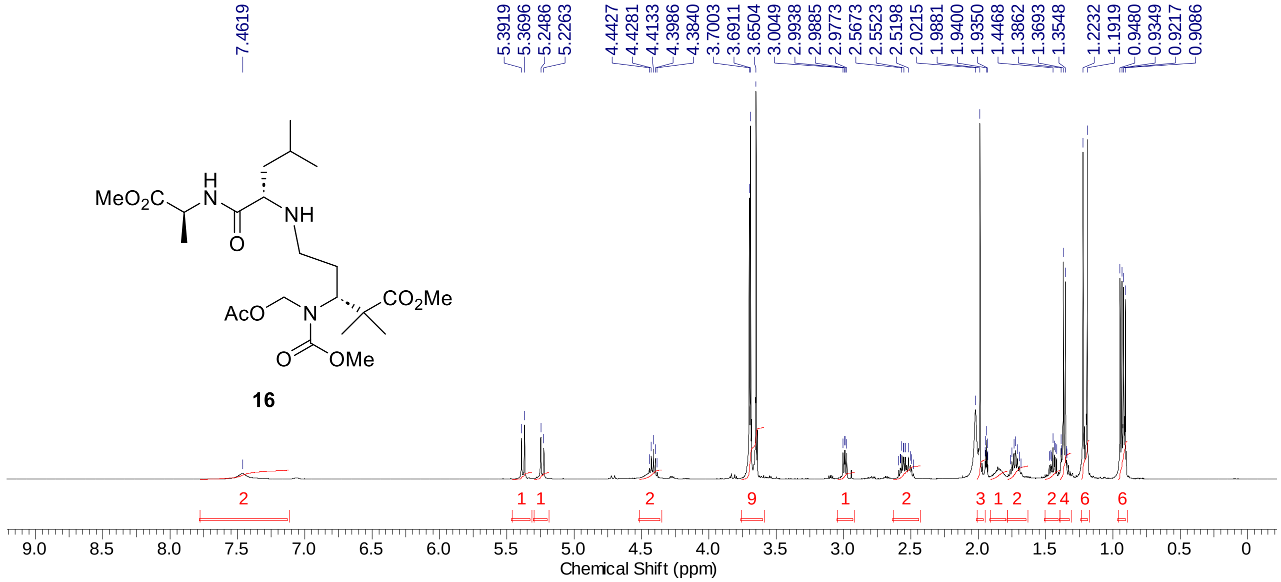

MeO2C
H
N
O
NH
AcO
N
CO2Me
O
OMe
16
7.4619
5.3919
5.3696
5.2486
5.2263
4.4427
4.4281
4.4133
4.3986
4.3840
3.7003
3.6911
3.6504
3.0049
2.9938
2.9885
2.9773
2.5673
2.5523
2.5198
2.0215
1.9881
1.9400
1.9350
1.4468
1.3862
1.3693
1.3548
1.2232
1.1919
0.9480
0.9349
0.9217
0.9086
2 1 1 2 9 1 2 3 1 2 2 4 6 6
9.0 8.5 8.0 7.5 7.0 6.5 6.0 5.5 5.0 4.5 4.0 3.5 3.0 2.5 2.0 1.5 1.0 0.5 0
Chemical Shift (ppm)

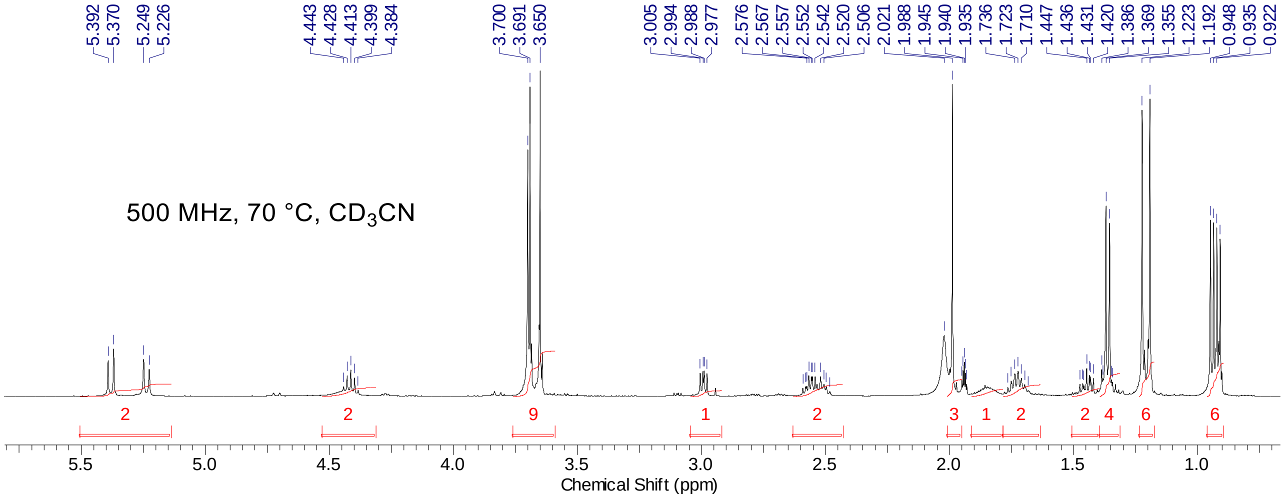

5.392
5.370
5.249
5.226
4.443
4.428
4.413
4.399
4.384
3.700
3.691
3.650
3.005
2.994
2.988
2.977
2.576
2.567
2.557
2.552
2.542
2.520
2.506
2.021
1.988
1.945
1.940
1.935
1.736
1.723
1.710
1.447
1.436
1.431
1.420
1.386
1.369
1.355
1.223
1.192
0.948
0.935
0.922
500 MHz, 70 °C, CD3CN
2 2 9 1 2 3 1 2 2 4 6 6
5.5 5.0 4.5 4.0 3.5 3.0 2.5 2.0 1.5 1.0
Chemical Shift (ppm)

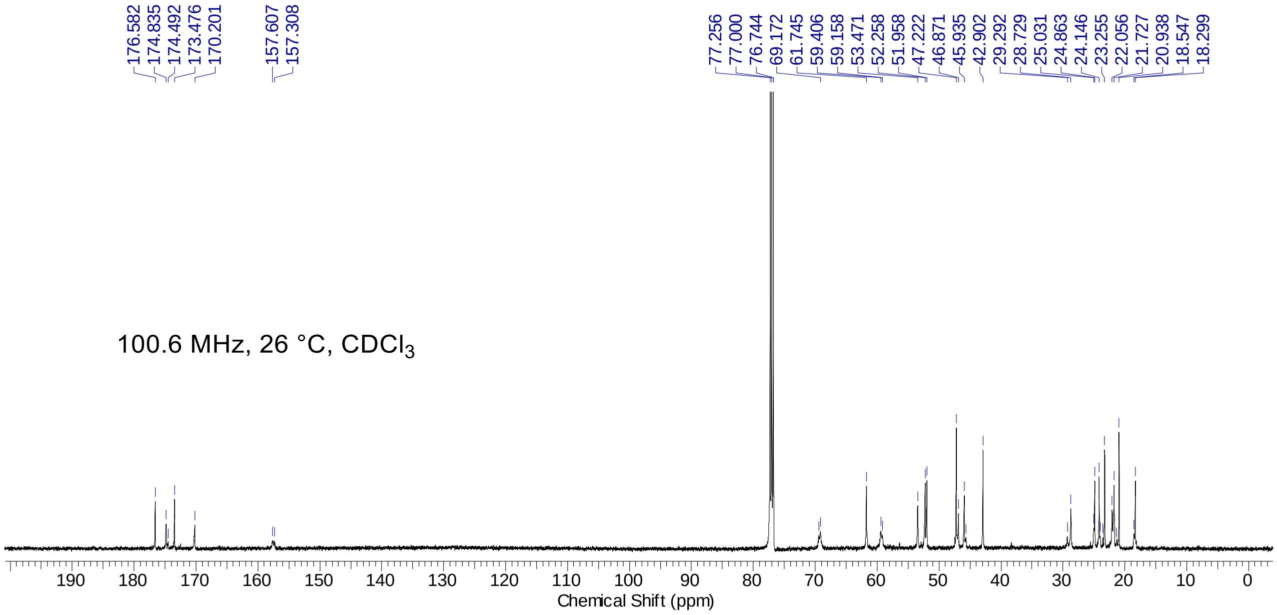

176.582
174.835
174.492
173.476
170.201
157.607
157.308
77.256
77.000
76.744
69.172
61.745
59.406
59.158
53.471
52.258
51.958
47.222
46.871
45.935
42.902
29.292
28.729
25.031
24.863
24.146
23.255
22.056
21.727
20.938
18.547
18.299
100.6 MHz, 26 °C, CDCl3
190 180 170 160 150 140 130 120 110 100 90 80 70 60 50 40 30 20 10 0
Chemical Shift (ppm)

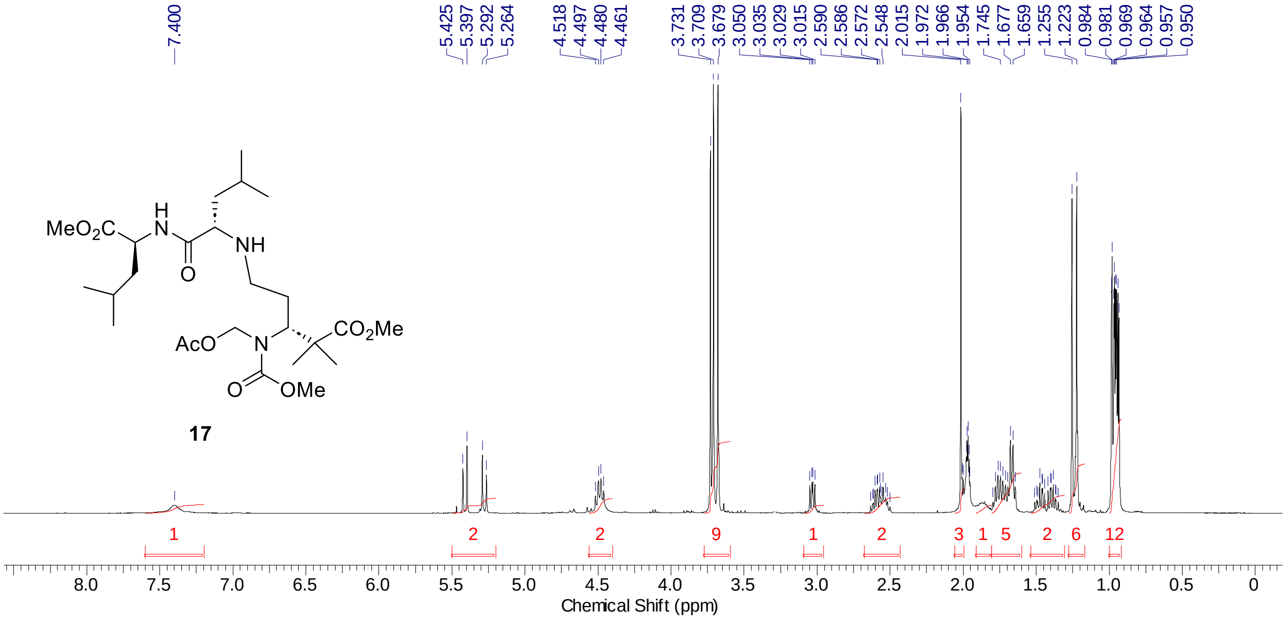
7.400
5.425
5.397
5.292
5.264
4.518
4.497
4.480
4.461
3.731
3.709
3.679
3.050
3.035
3.029
3.015
2.590
2.586
2.572
2.548
2.015
1.972
1.966
1.954
1.745
1.677
1.659
1.255
1.223
0.984
0.981
0.969
0.964
0.957
0.950
MeO2C
H
N
O
NH
AcO
N
CO2Me
O
OMe
17
1
2
2
9
1
2
3
1
5
2
6
12
8.0 7.5 7.0 6.5 6.0 5.5 5.0 4.5 4.0 3.5 3.0 2.5 2.0 1.5 1.0 0.5 0
Chemical Shift (ppm)

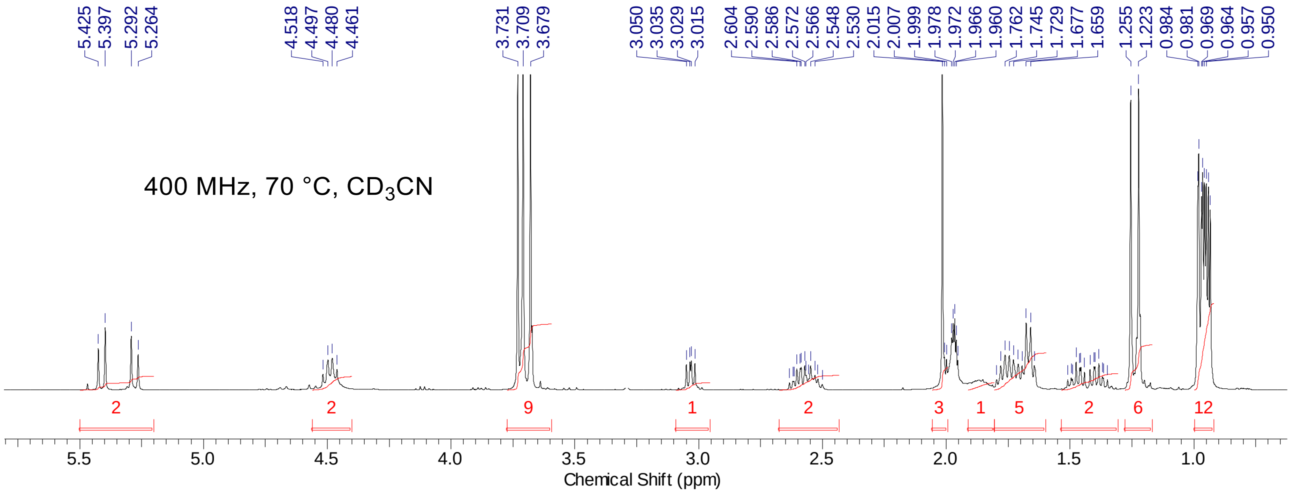
5.425
5.397
5.292
5.264
4.518
4.497
4.480
4.461
3.731
3.709
3.679
3.050
3.035
3.029
3.015
2.604
2.590
2.586
2.572
2.566
2.548
2.530
2.015
2.007
1.999
1.978
1.972
1.966
1.960
1.762
1.745
1.729
1.677
1.659
1.255
1.223
0.984
0.981
0.969
0.964
0.957
0.950
400 MHz, 70 °C, CD3CN
2
2
9
1
2
3
1
5
2
6
12
5.5 5.0 4.5 4.0 3.5 3.0 2.5 2.0 1.5 1.0
Chemical Shift (ppm)

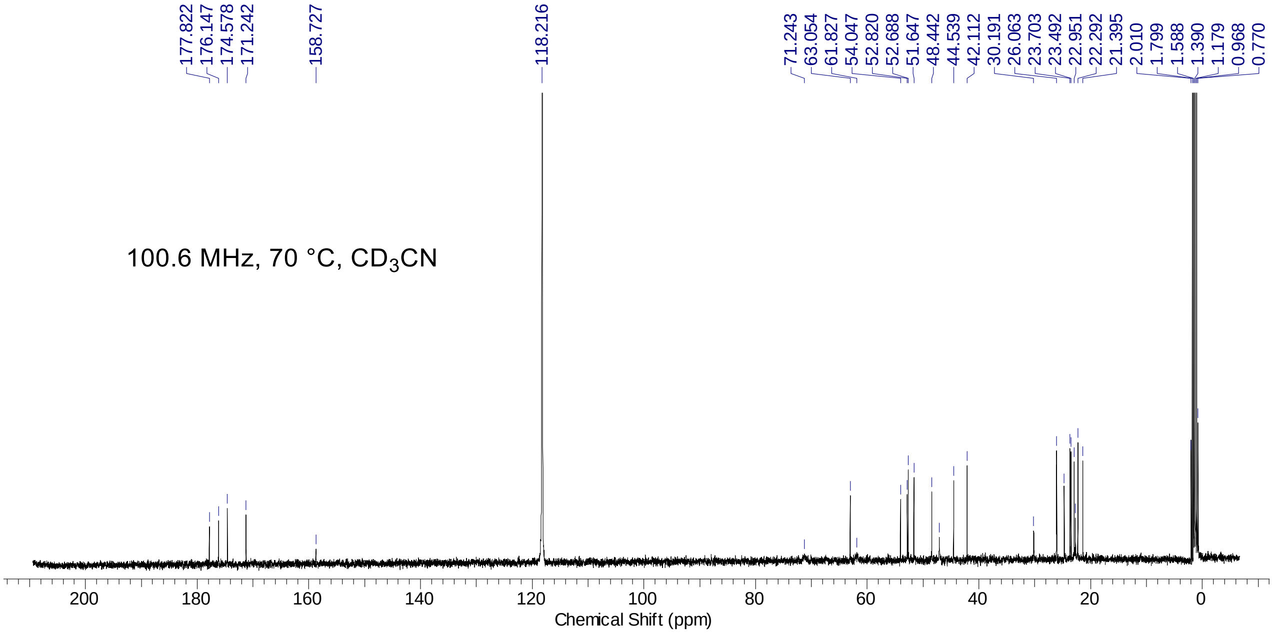
177.822
176.147
174.578
171.242
158.727
118.216
71.243
63.054
61.827
54.047
52.820
52.688
51.647
48.442
44.539
42.112
30.191
26.063
23.703
23.492
22.951
22.292
21.395
2.010
1.799
1.588
1.390
1.179
0.968
0.770
100.6 MHz, 70 °C, CD3CN
200 180 160 140 120 100 80 60 40 20 0
Chemical Shift (ppm)

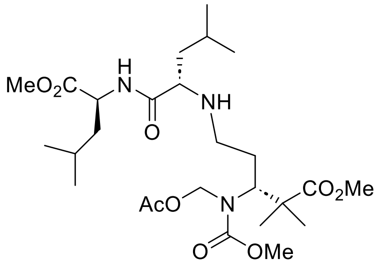

17

HSQC, 70 °C, CD_3CN

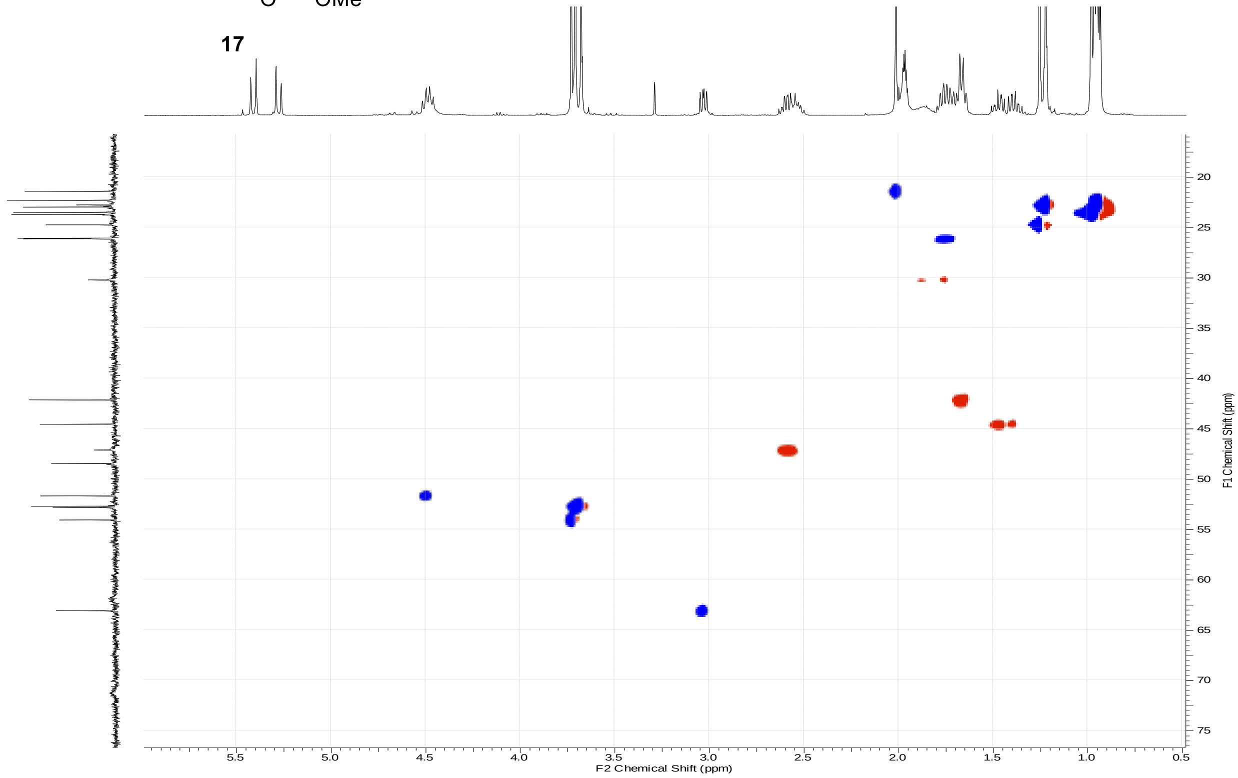

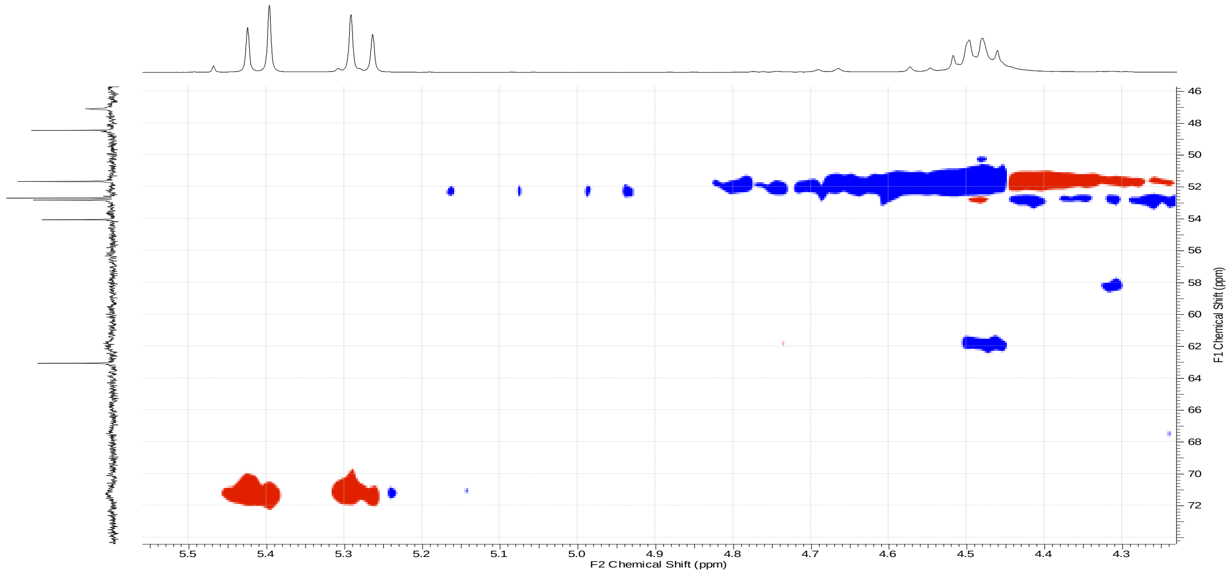

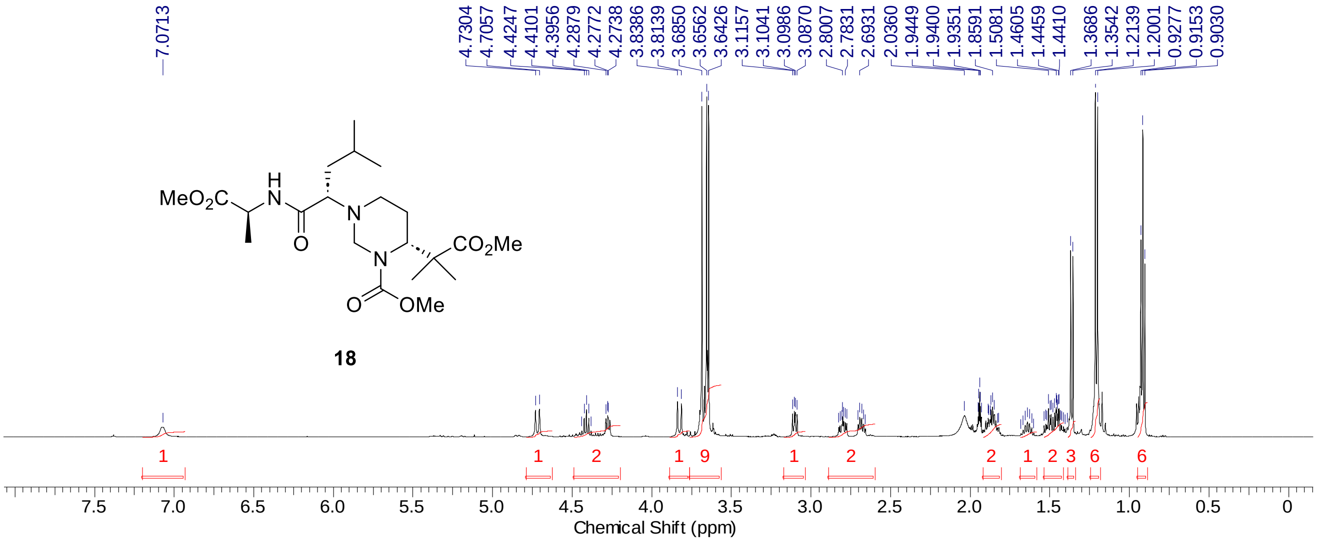

18

7.0713
4.7304 4.7057 4.4247 4.4101 4.3956 4.2879 4.2772 4.2738 3.8386 3.8139 3.6850 3.6562 3.6426 3.1157 3.1041 3.0986 3.0870 2.8007 2.7831 2.6931 2.0360 1.9449 1.9400 1.9351 1.8591 1.5081 1.4605 1.4459 1.4410 1.3686 1.3542 1.2139 1.2001 0.9277 0.9153 0.9030
1 1 2 1 9 1 2 2 1 2 3 6 6
Chemical Shift (ppm)

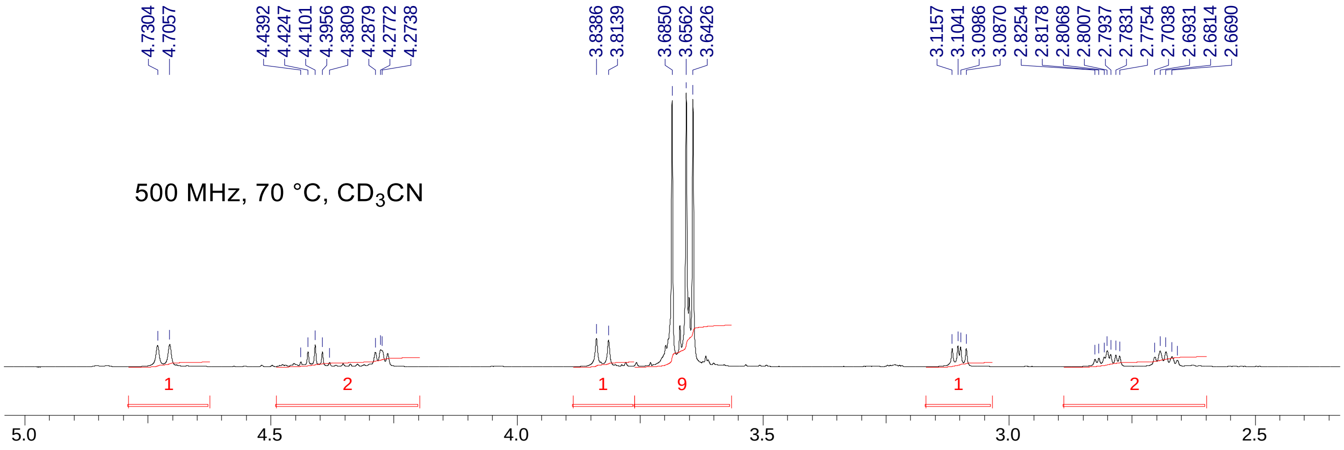
4.7304 4.7057 4.4392 4.4247 4.4101 4.3956 4.3809 4.2879 4.2772 4.2738 3.8386 3.8139 3.6850 3.6562 3.6426 3.1157 3.1041 3.0986 3.0870 2.8254 2.8178 2.8068 2.8007 2.7937 2.7831 2.7754 2.7038 2.6931 2.6814 2.6690
500 MHz, 70 °C, CD3CN
1 2 1 9 1 2

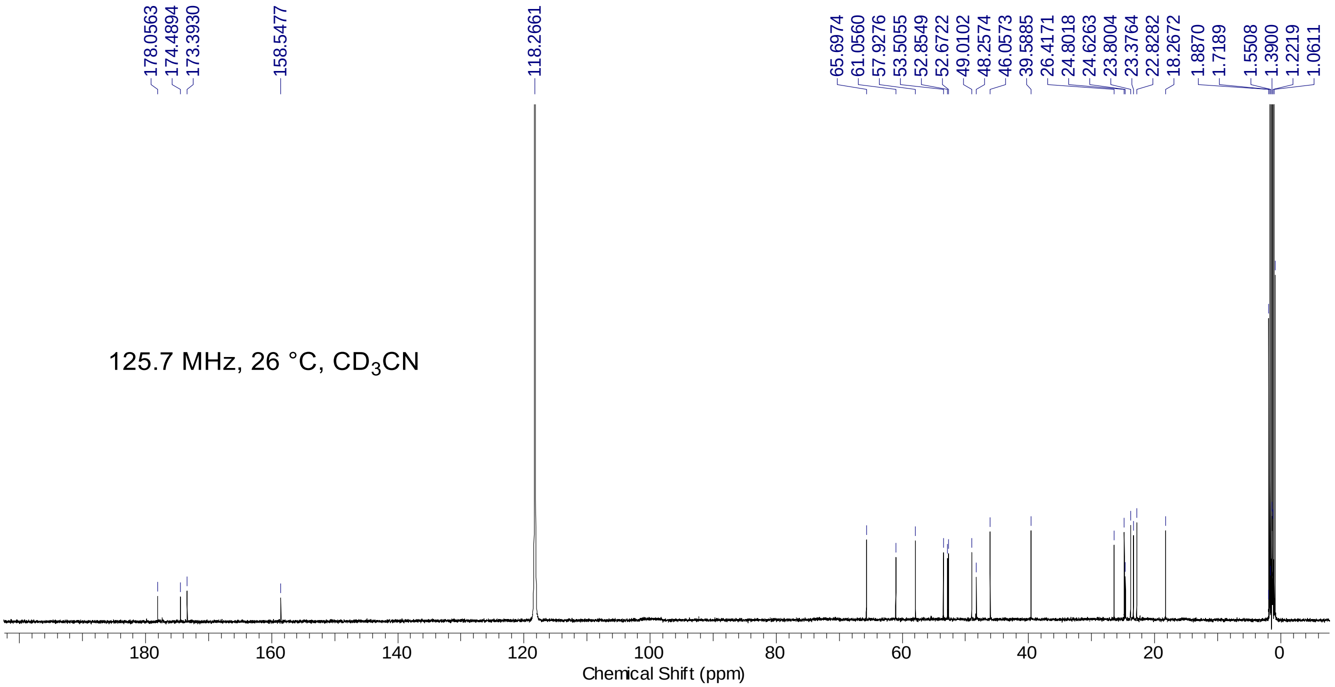
178.0563 174.4894 173.3930 158.5477 118.2661 65.6974 61.0560 57.9276 53.5055 52.8549 52.6722 49.0102 48.2574 46.0573 39.5885 26.4171 24.8018 24.6263 23.8004 23.3764 22.8282 18.2672 1.8870 1.7189 1.5508 1.3900 1.2219 1.0611
125.7 MHz, 26 °C, CD3CN
Chemical Shift (ppm)

DA1-94-A.HSQC 70.1R.ESP

HSQC 70 °C

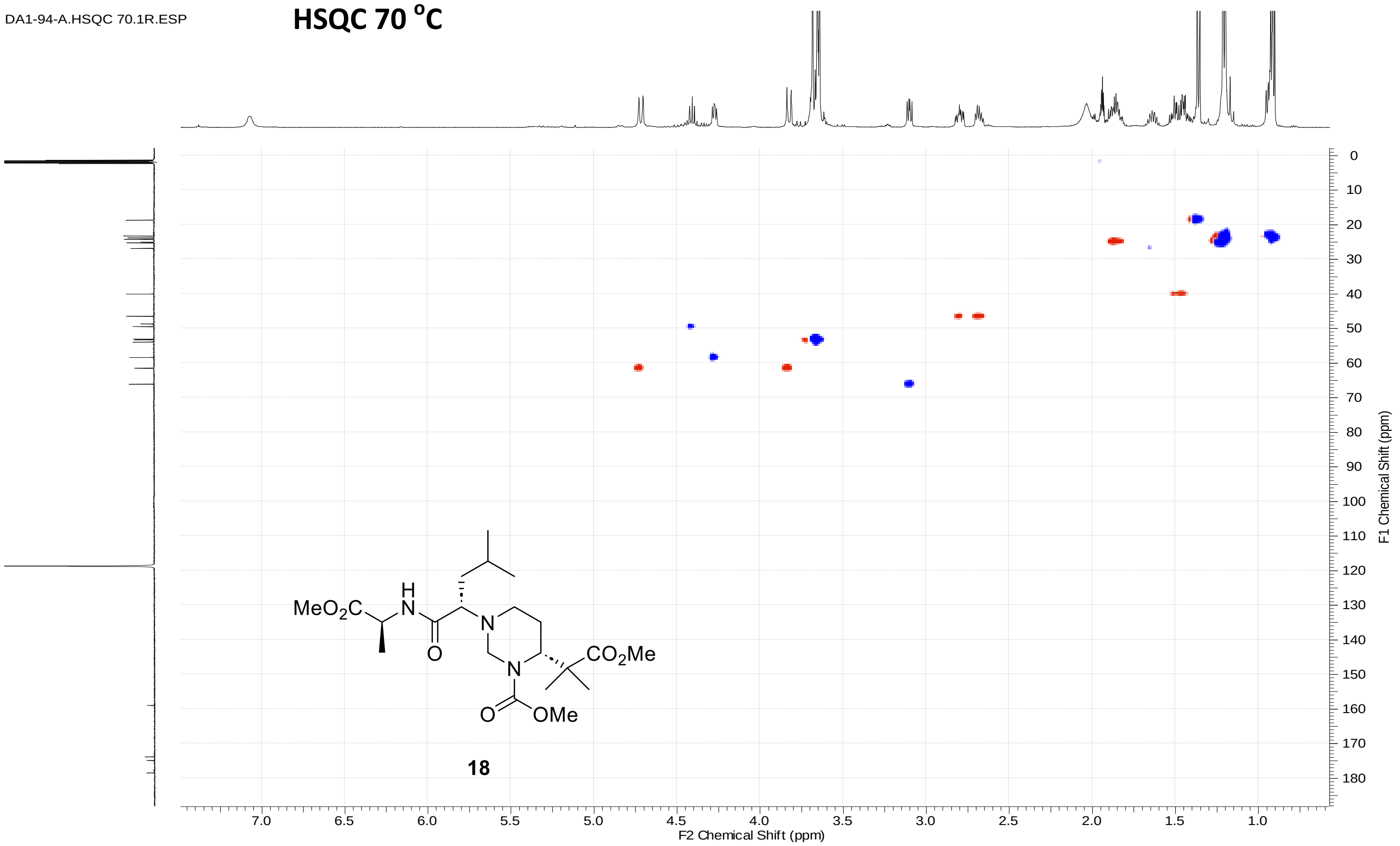

DA1-94-A.HSQC 70.1R.ESP

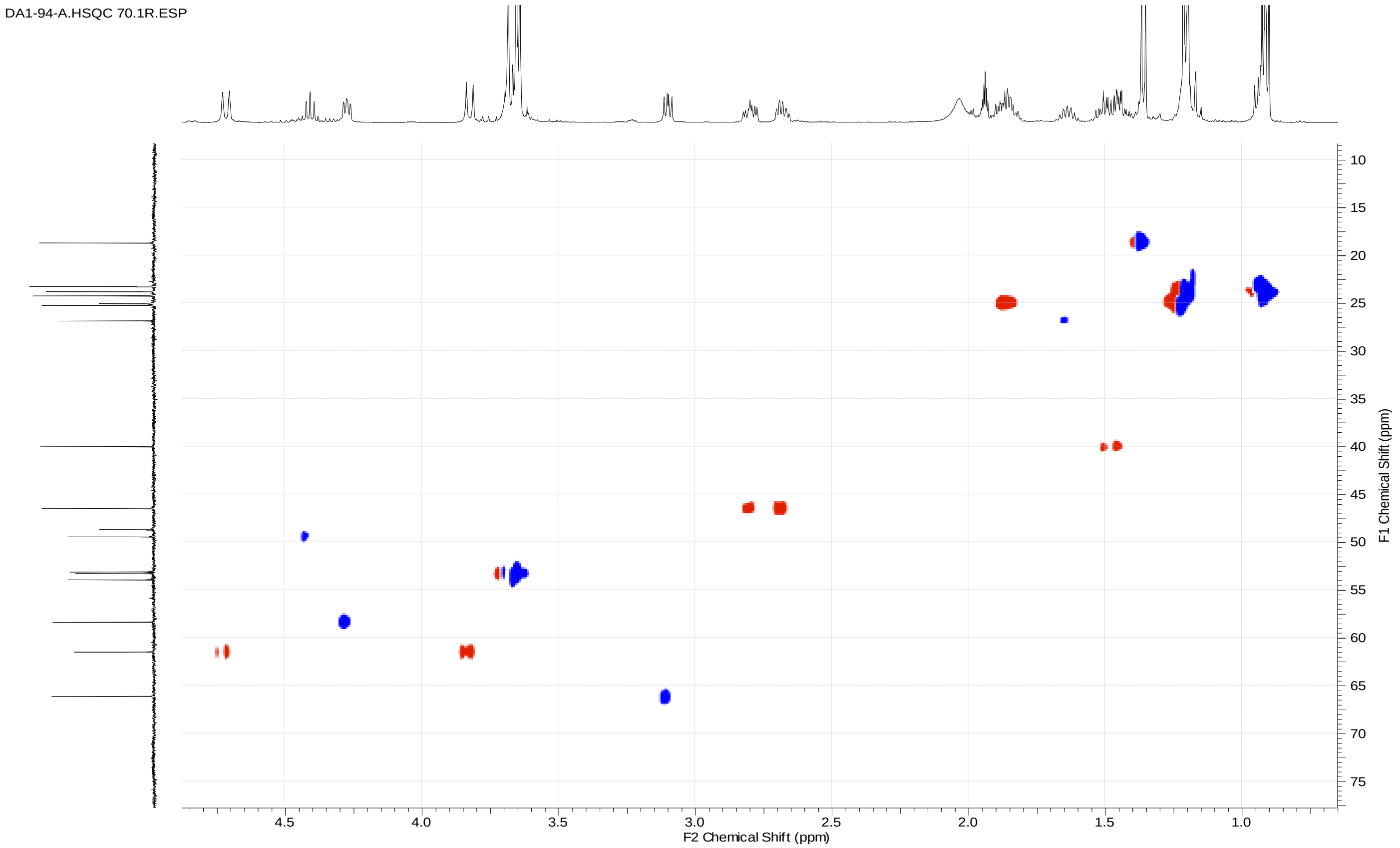

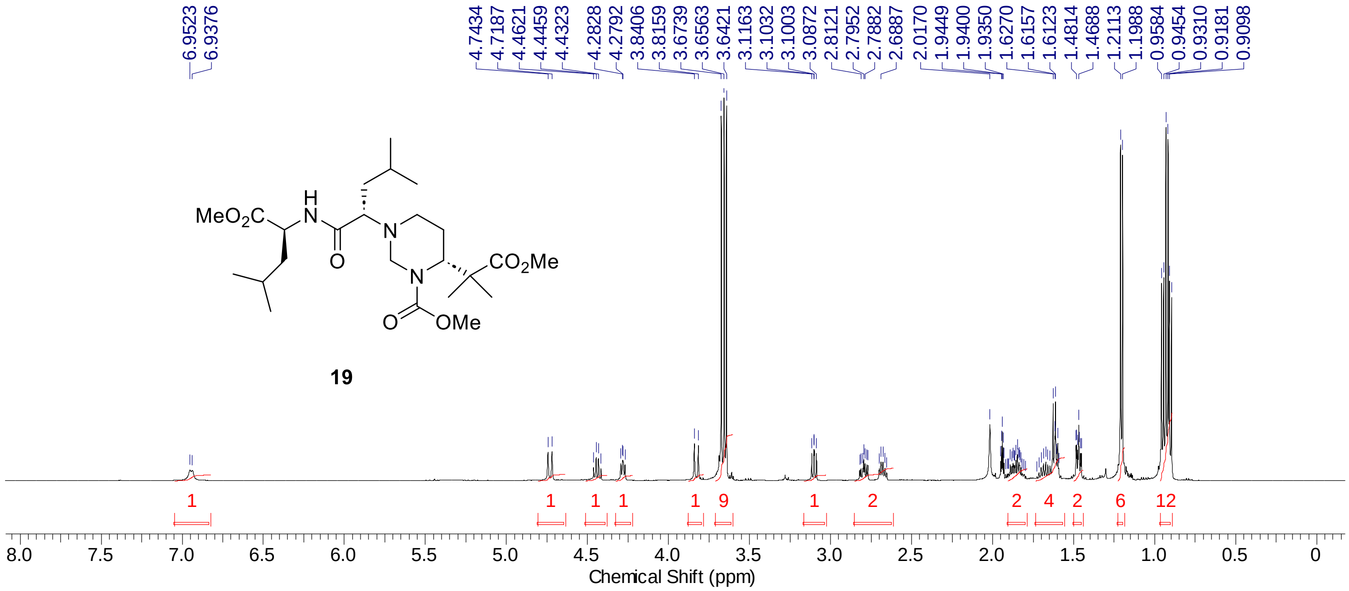
6.9523
6.9376
4.7434
4.7187
4.4621
4.4459
4.4323
4.2828
4.2792
3.8406
3.8159
3.6739
3.6563
3.6421
3.1163
3.1032
3.1003
3.0872
2.8121
2.7952
2.7882
2.6887
2.0170
1.9449
1.9400
1.9350
1.6270
1.6157
1.6123
1.4814
1.4688
1.2113
1.1988
0.9584
0.9454
0.9310
0.9181
0.9098
1 1 1 1 1 9 1 2 2 4 2 6 12
8.0 7.5 7.0 6.5 6.0 5.5 5.0 4.5 4.0 3.5 3.0 2.5 2.0 1.5 1.0 0.5 0
Chemical Shift (ppm)

19

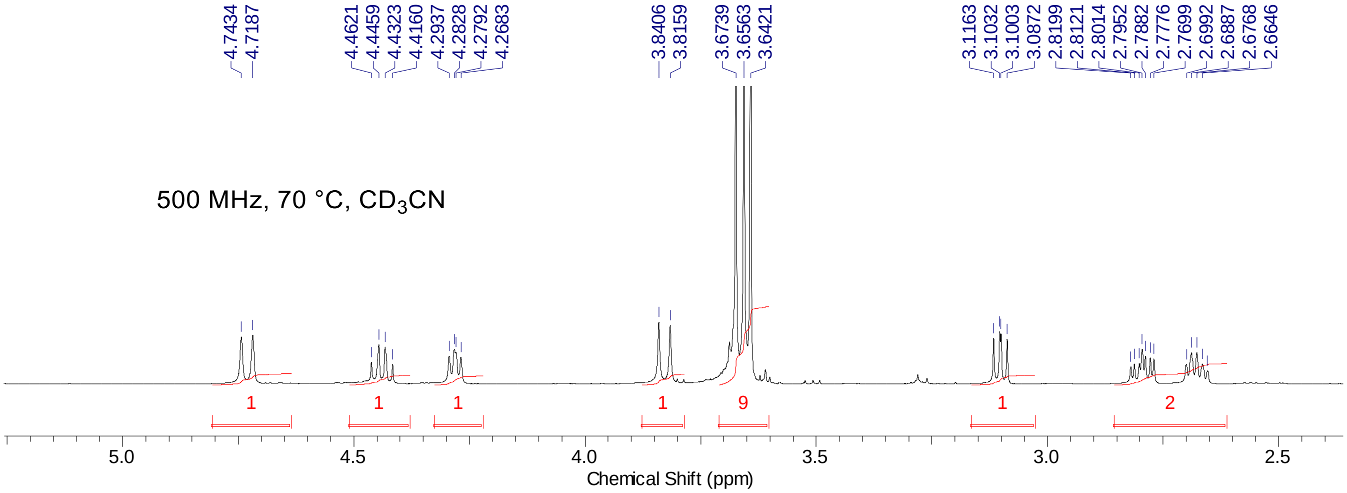
4.7434
4.7187
4.4621
4.4459
4.4323
4.4160
4.2937
4.2828
4.2792
4.2683
3.8406
3.8159
3.6739
3.6563
3.6421
3.1163
3.1032
3.1003
3.0872
2.8199
2.8121
2.8014
2.7952
2.7882
2.7776
2.7699
2.6992
2.6887
2.6768
2.6646
500 MHz, 70 °C, CD3CN
1 1 1 1 9 1 2
5.0 4.5 4.0 3.5 3.0 2.5
Chemical Shift (ppm)

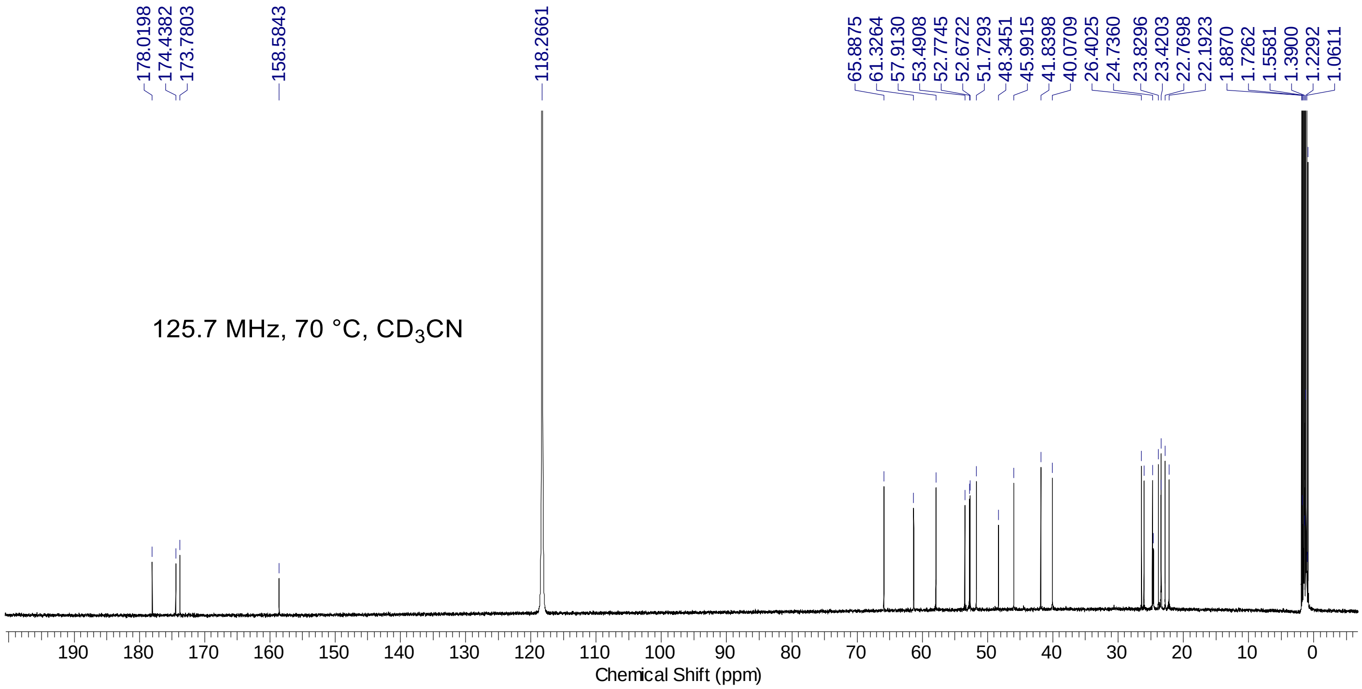
178.0198
174.4382
173.7803
158.5843
118.2661
65.8875
61.3264
57.9130
53.4908
52.7745
52.6722
51.7293
48.3451
45.9915
41.8398
40.0709
26.4025
24.7360
23.8296
23.4203
22.7698
22.1923
1.8870
1.7262
1.5581
1.3900
1.2292
1.0611
125.7 MHz, 70 °C, CD3CN
190 180 170 160 150 140 130 120 110 100 90 80 70 60 50 40 30 20 10 0
Chemical Shift (ppm)

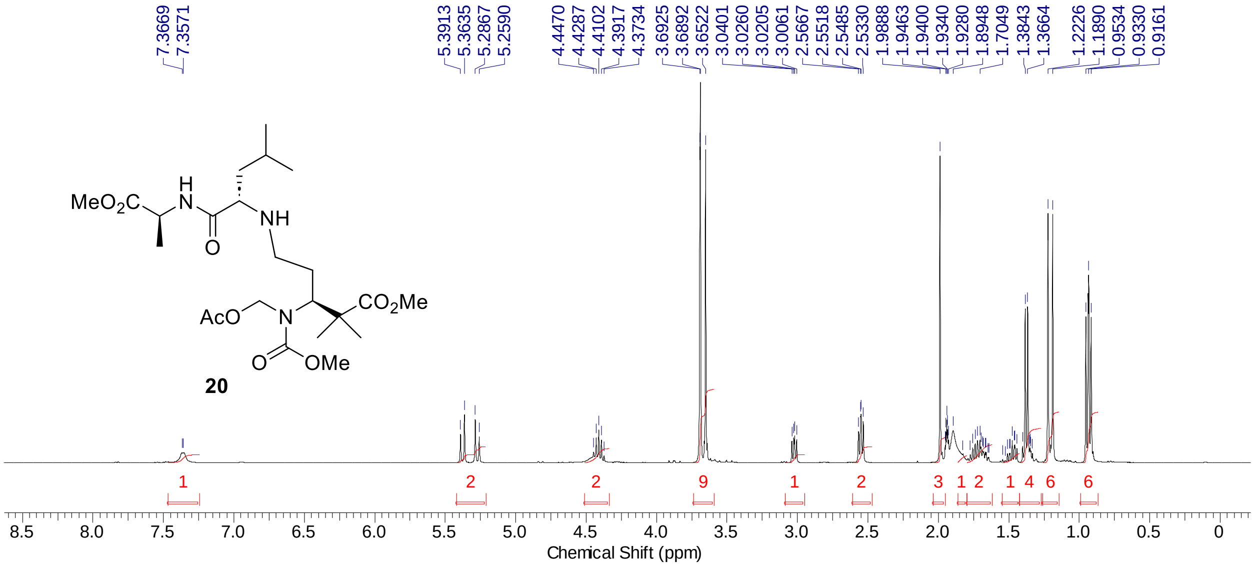
7.3669
7.3571
5.3913
5.3635
5.2867
5.2590
4.4470
4.4287
4.4102
4.3917
4.3734
3.6925
3.6892
3.6522
3.0401
3.0260
3.0205
3.0061
2.5667
2.5518
2.5485
2.5330
1.9888
1.9463
1.9400
1.9340
1.9280
1.8948
1.7049
1.3843
1.3664
1.2226
1.1890
0.9534
0.9330
0.9161
MeO2C
H
N
O
NH
AcO
N
CO2Me
O
OMe
20
1 2 2 9 1 2 3 1 2 1 4 6 6
Chemical Shift (ppm)

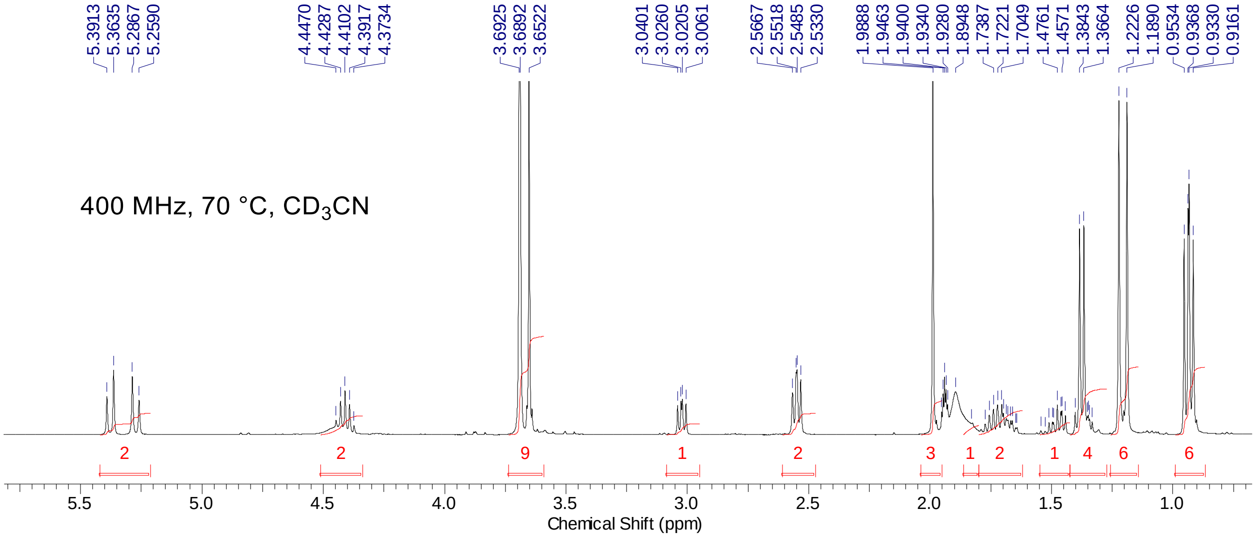
5.3913
5.3635
5.2867
5.2590
4.4470
4.4287
4.4102
4.3917
4.3734
3.6925
3.6892
3.6522
3.0401
3.0260
3.0205
3.0061
2.5667
2.5518
2.5485
2.5330
1.9888
1.9463
1.9400
1.9340
1.9280
1.8948
1.7387
1.7221
1.7049
1.4761
1.4571
1.3843
1.3664
1.2226
1.1890
0.9534
0.9368
0.9330
0.9161
400 MHz, 70 °C, CD3CN
2 2 9 1 2 3 1 2 1 4 6 6
Chemical Shift (ppm)

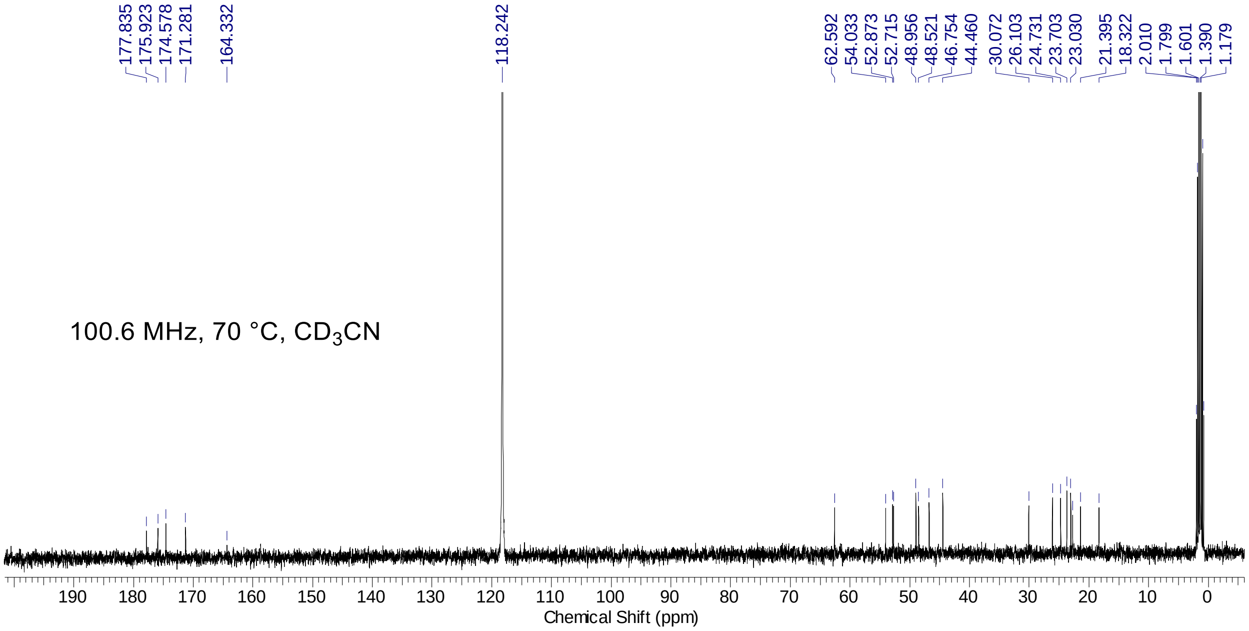
177.835
175.923
174.578
171.281
164.332
118.242
62.592
54.033
52.873
52.715
48.956
48.521
46.754
44.460
30.072
26.103
24.731
23.703
23.030
21.395
18.322
2.010
1.799
1.601
1.390
1.179
100.6 MHz, 70 °C, CD3CN
Chemical Shift (ppm)

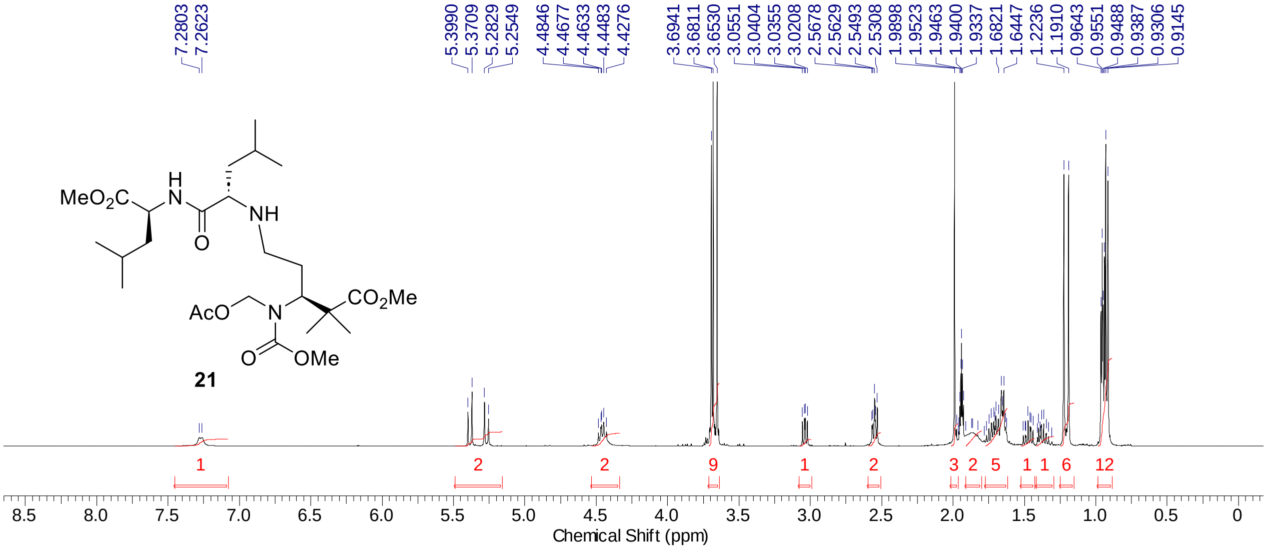
7.2803
7.2623
5.3990
5.3709
5.2829
5.2549
4.4846
4.4677
4.4633
4.4483
4.4276
3.6941
3.6811
3.6530
3.0551
3.0404
3.0355
3.0208
2.5678
2.5629
2.5493
2.5308
1.9898
1.9523
1.9463
1.9400
1.9337
1.6821
1.6447
1.2236
1.1910
0.9643
0.9551
0.9488
0.9387
0.9306
0.9145
MeO$_2$C
H
N
O
NH
CO$_2$Me
AcO
N
O
OMe
21
1
2
2
9
1
2
3
2
5
1
1
6
12
Chemical Shift (ppm)

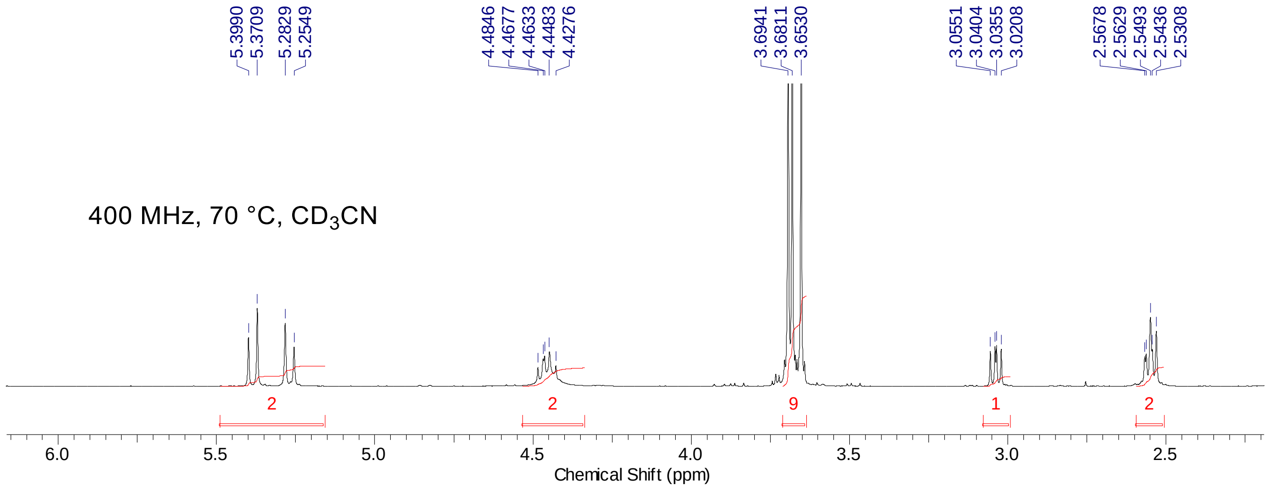
5.3990
5.3709
5.2829
5.2549
4.4846
4.4677
4.4633
4.4483
4.4276
3.6941
3.6811
3.6530
3.0551
3.0404
3.0355
3.0208
2.5678
2.5629
2.5493
2.5436
2.5308
400 MHz, 70 °C, CD$_3$CN
2
2
9
1
2
Chemical Shift (ppm)

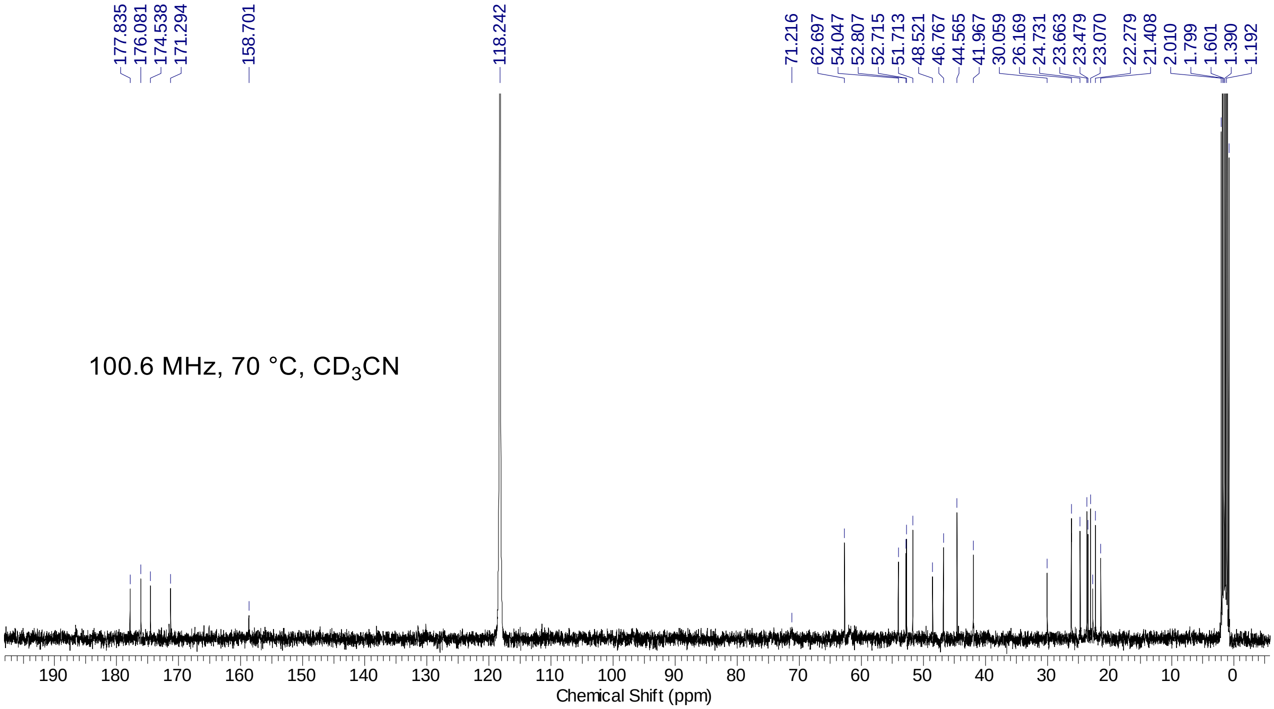
177.835
176.081
174.538
171.294
158.701
118.242
71.216
62.697
54.047
52.807
52.715
51.713
48.521
46.767
44.565
41.967
30.059
26.169
24.731
23.663
23.479
23.070
22.279
21.408
2.010
1.799
1.601
1.390
1.192
100.6 MHz, 70 °C, CD$_3$CN
Chemical Shift (ppm)

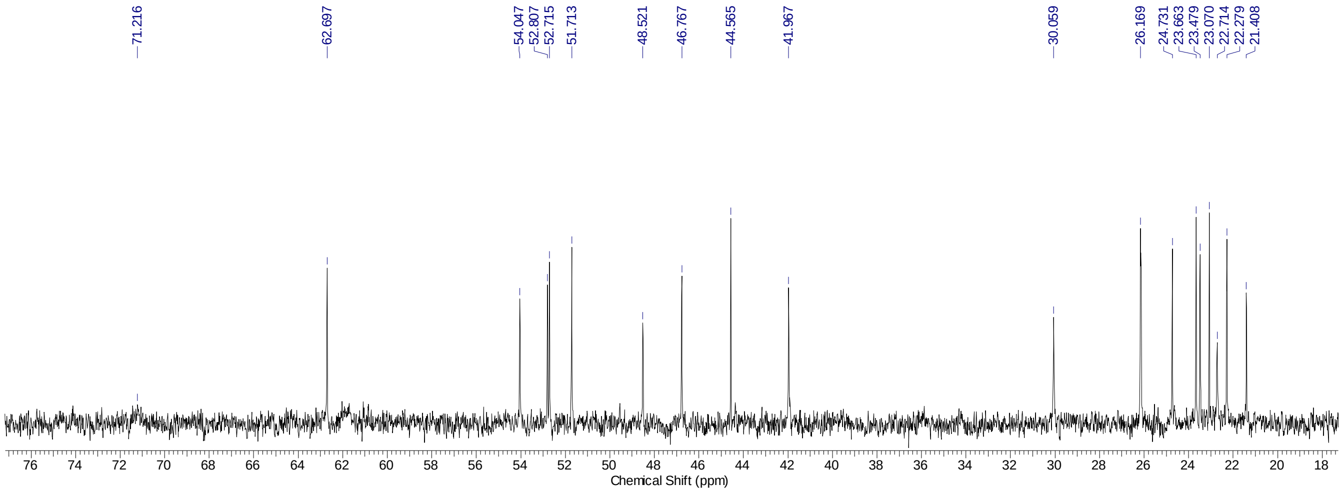

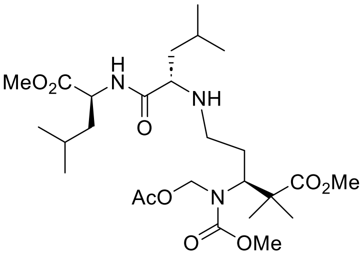

21

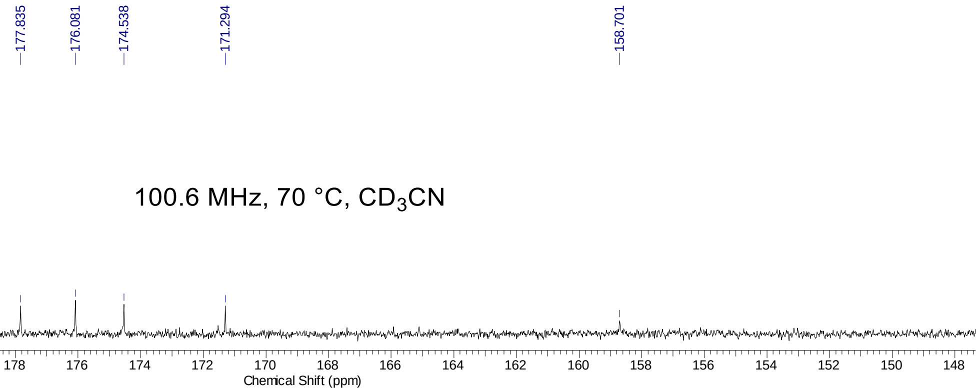

100.6 MHz, 70 °C, CD_3CN

COSY, 400 MHz, 70 °C, CD_3CN

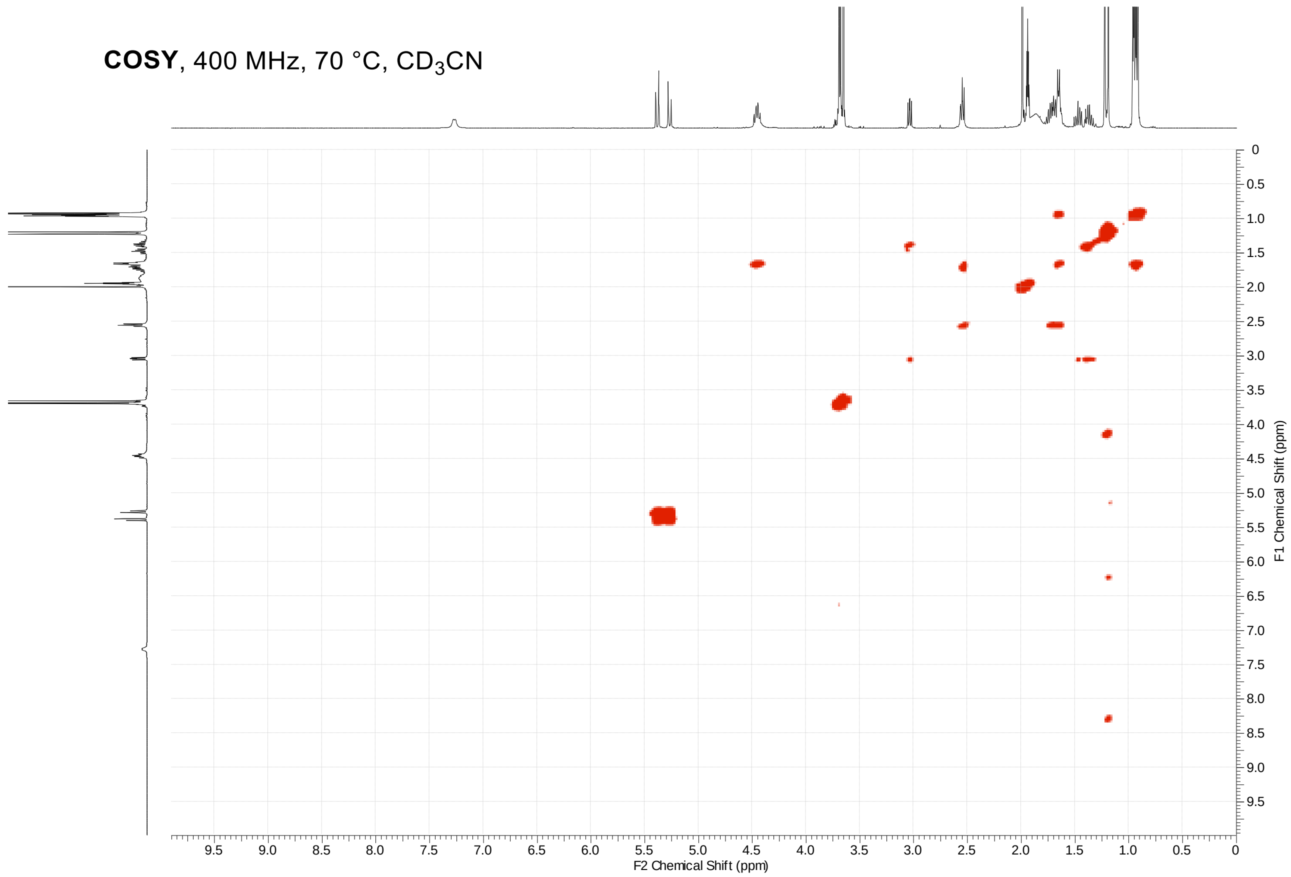

HSQC
70 °C, CD_3CN

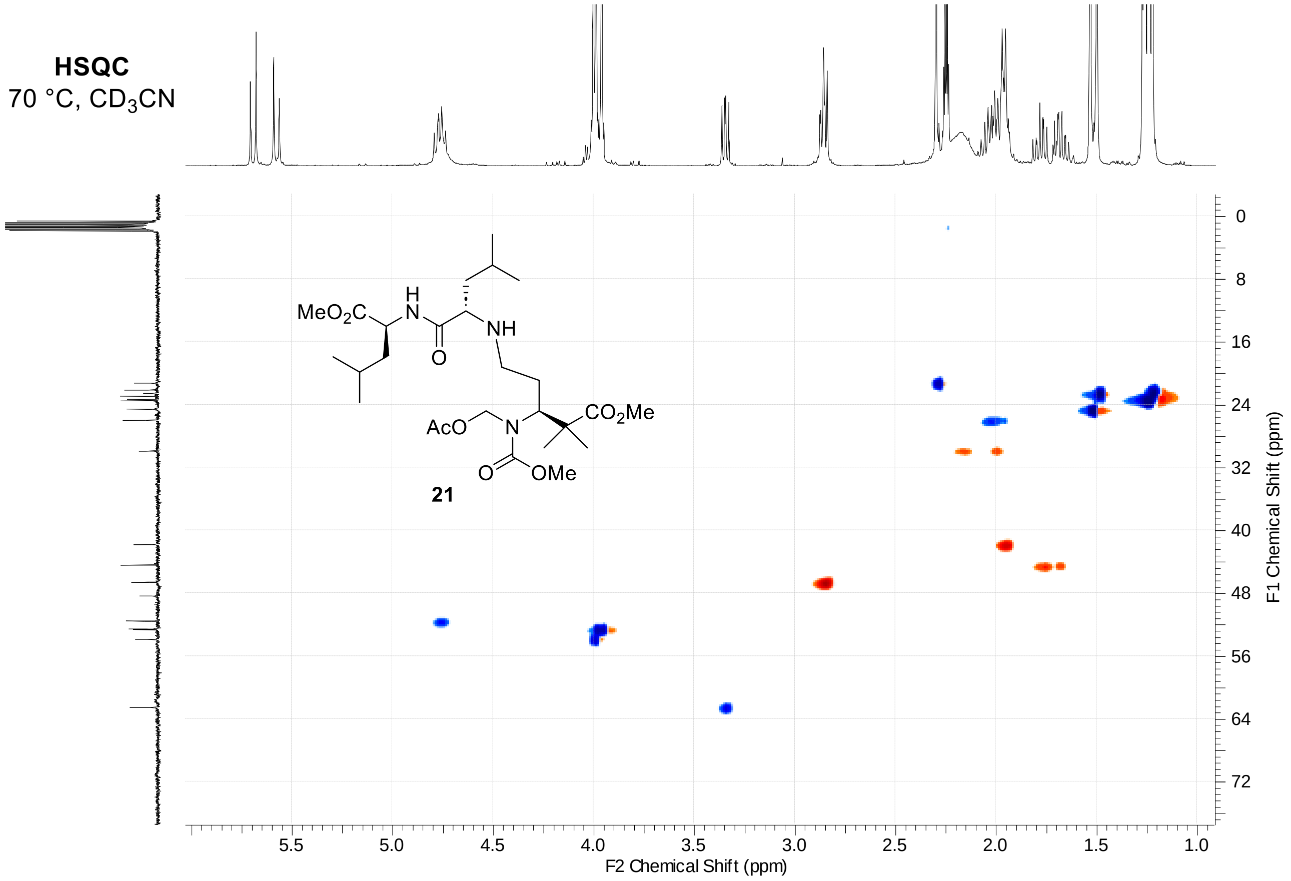

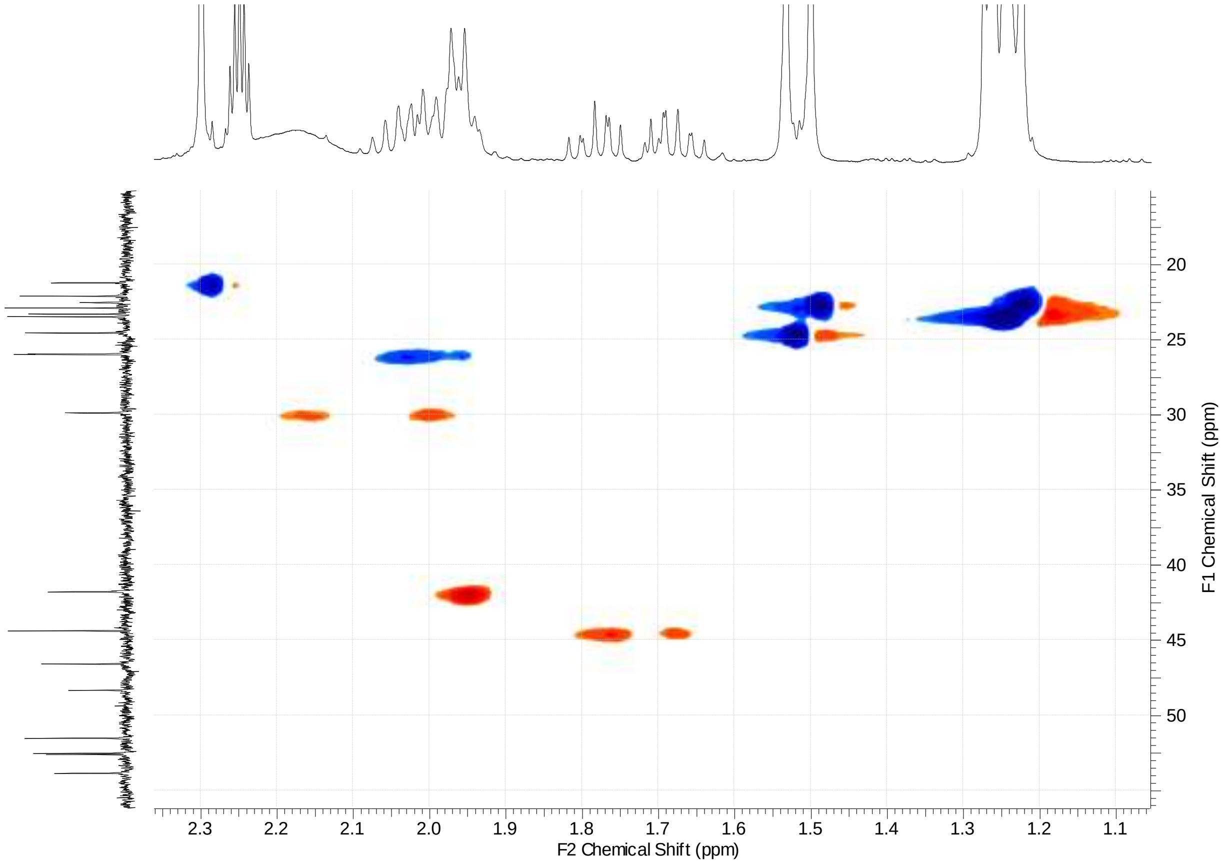

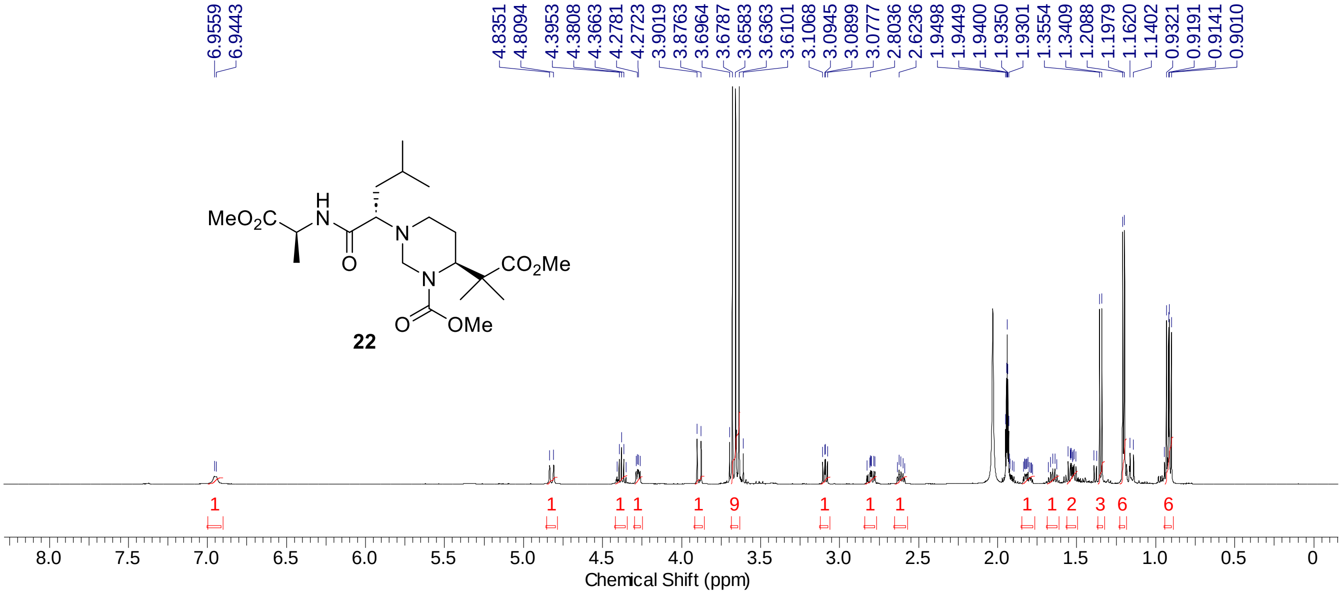

MeO$_2$C
H
N
N
O
N
CO$_2$Me
O
OMe
22
6.9559
6.9443
4.8351
4.8094
4.3953
4.3808
4.3663
4.2781
4.2723
3.9019
3.8763
3.6964
3.6787
3.6583
3.6363
3.6101
3.1068
3.0945
3.0899
3.0777
2.8036
2.6236
1.9498
1.9449
1.9400
1.9350
1.9301
1.3554
1.3409
1.2088
1.1979
1.1620
1.1402
0.9321
0.9191
0.9141
0.9010
1 1 1 1 1 9 1 1 1 1 1 2 3 6 6
8.0 7.5 7.0 6.5 6.0 5.5 5.0 4.5 4.0 3.5 3.0 2.5 2.0 1.5 1.0 0.5 0
Chemical Shift (ppm)

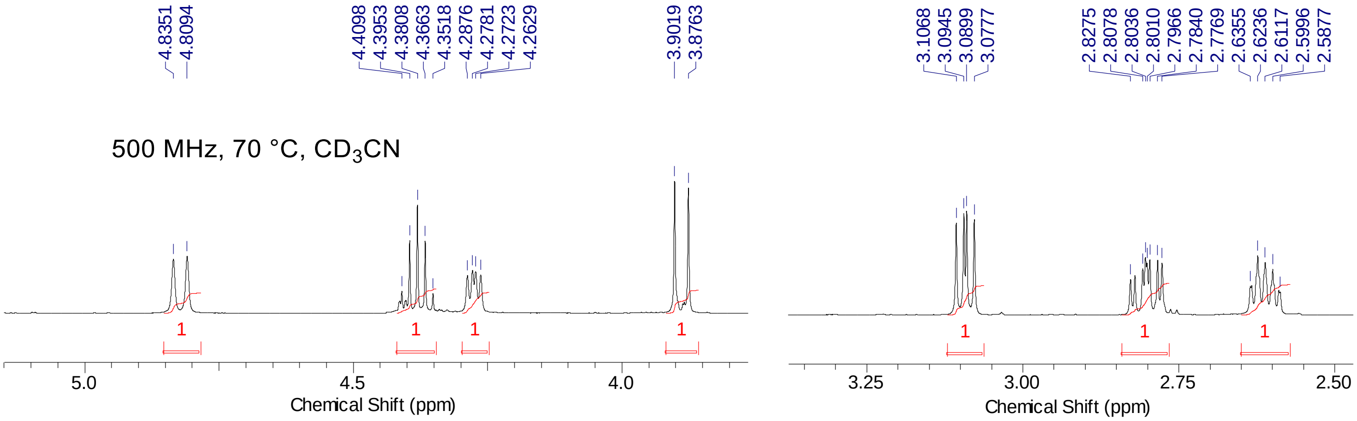

4.8351
4.8094
4.4098
4.3953
4.3808
4.3663
4.3518
4.2876
4.2781
4.2723
4.2629
3.9019
3.8763
500 MHz, 70 °C, CD$_3$CN
1 1 1 1
5.0 4.5 4.0
Chemical Shift (ppm)
3.1068
3.0945
3.0899
3.0777
2.8275
2.8078
2.8036
2.8010
2.7966
2.7840
2.7769
2.6355
2.6236
2.6117
2.5996
2.5877
1 1 1
3.25 3.00 2.75 2.50
Chemical Shift (ppm)

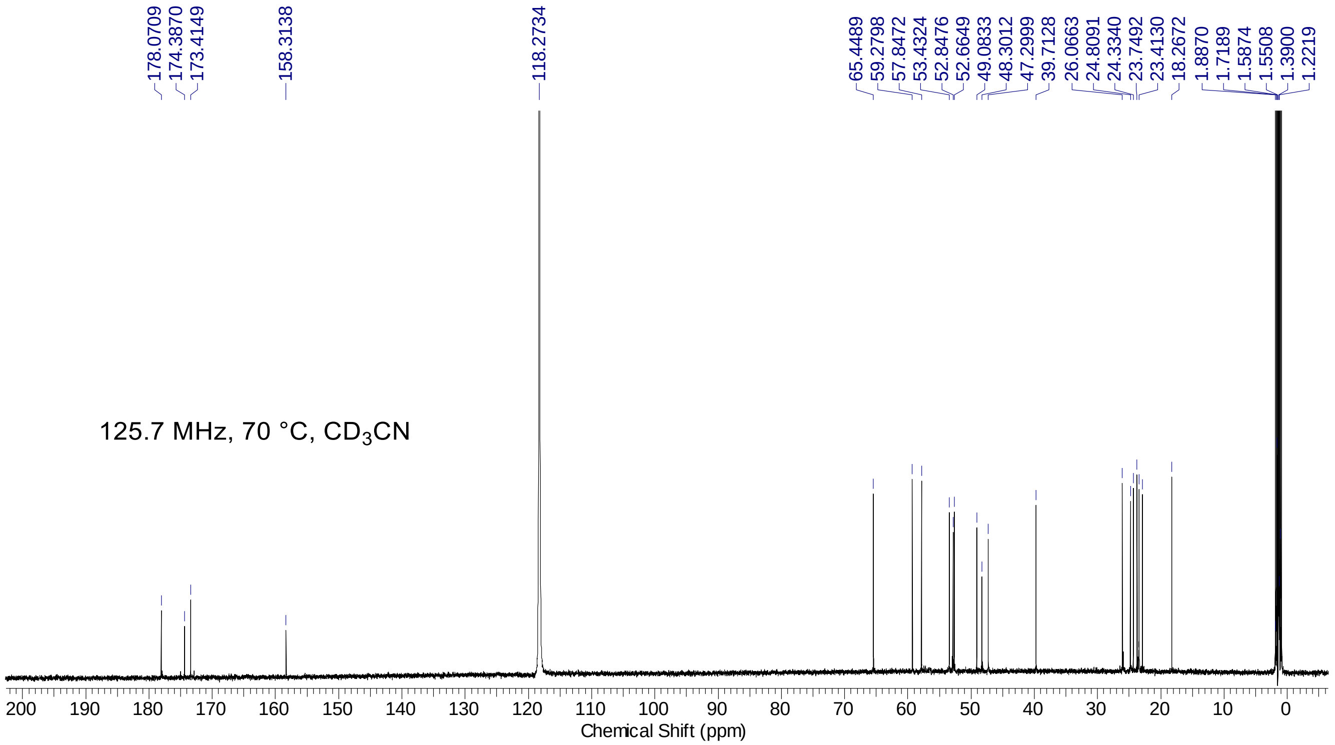

178.0709
174.3870
173.4149
158.3138
118.2734
65.4489
59.2798
57.8472
53.4324
52.8476
52.6649
49.0833
48.3012
47.2999
39.7128
26.0663
24.8091
24.3340
23.7492
23.4130
18.2672
1.8870
1.7189
1.5874
1.5508
1.3900
1.2219
125.7 MHz, 70 °C, CD$_3$CN
200 190 180 170 160 150 140 130 120 110 100 90 80 70 60 50 40 30 20 10 0
Chemical Shift (ppm)

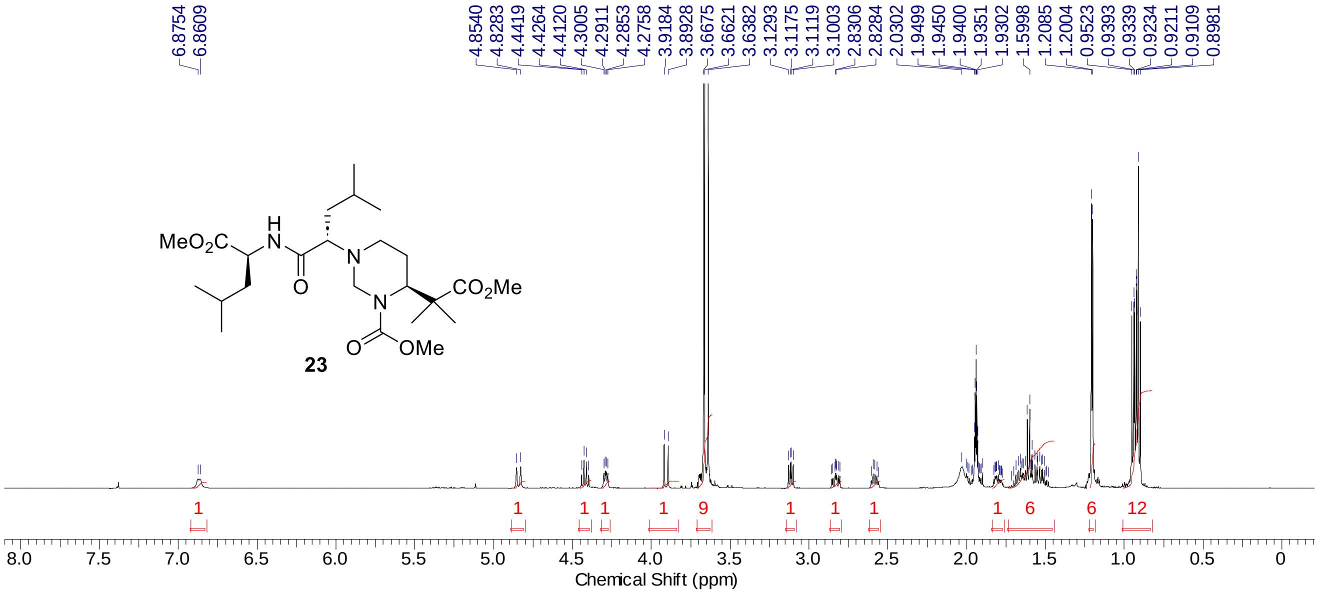
23
Chemical Shift (ppm)

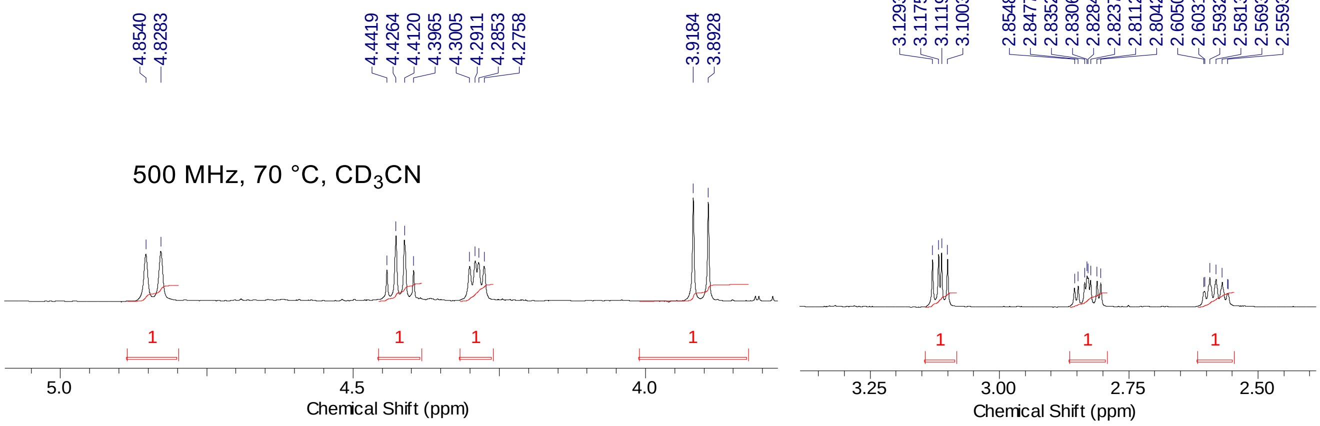
500 MHz, 70 °C, CD_3CN
Chemical Shift (ppm)
Chemical Shift (ppm)

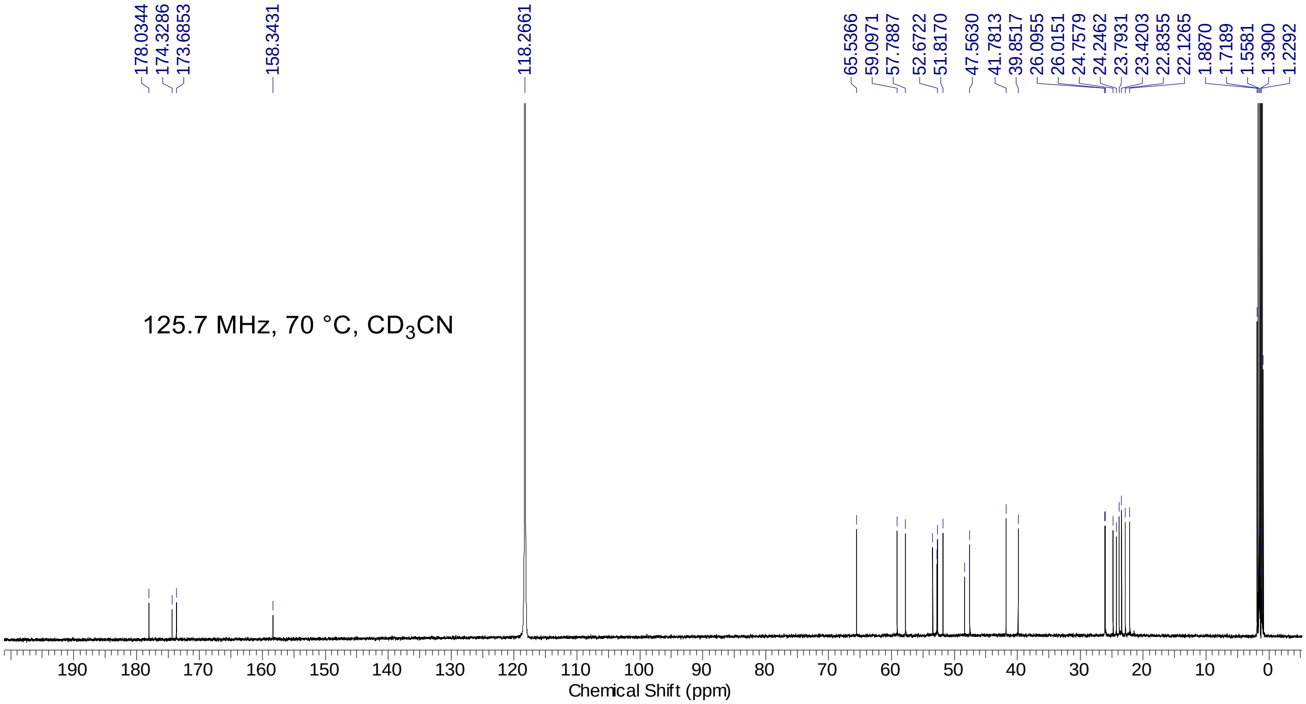
125.7 MHz, 70 °C, CD_3CN
Chemical Shift (ppm)

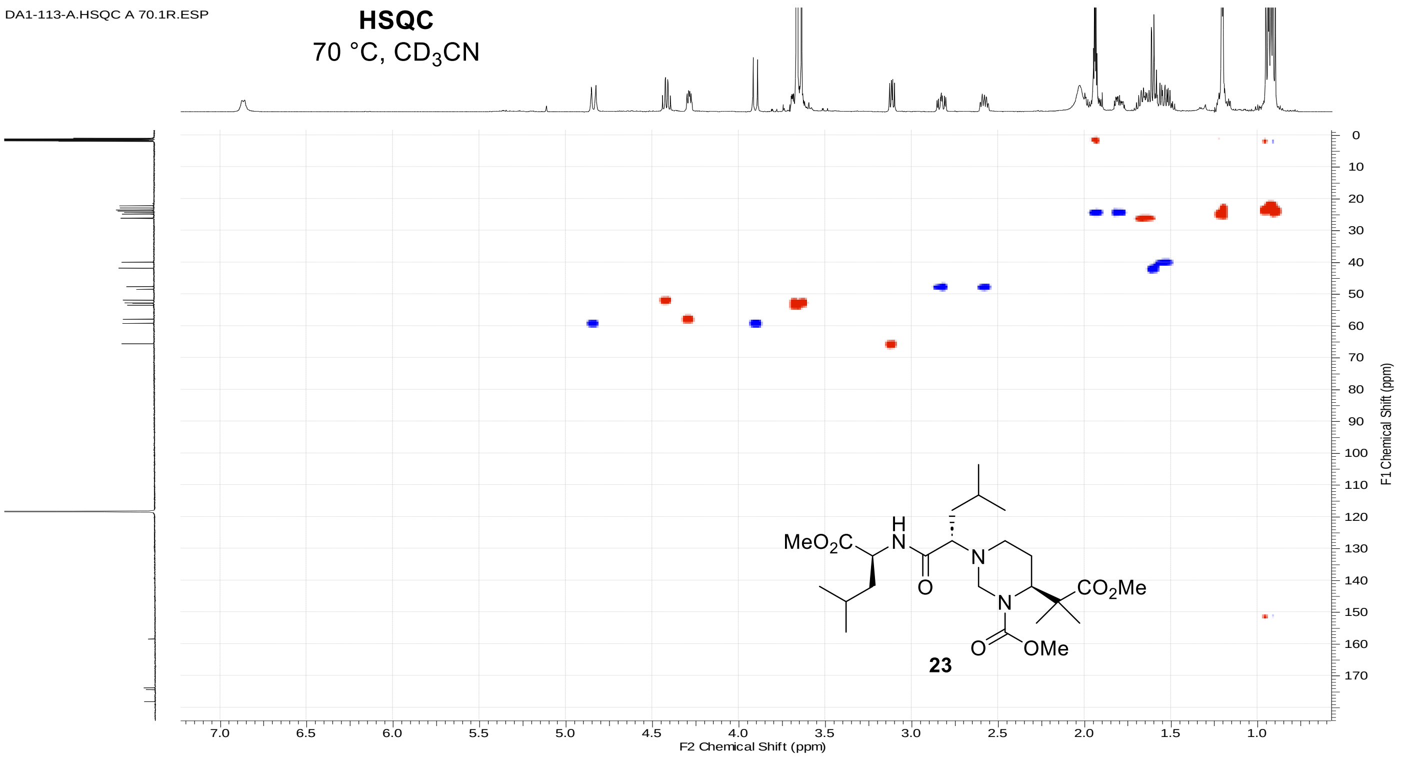
DA1-113-A.HSQC A 70.1R.ESP
HSQC
70 °C, CD3CN
MeO2C
H
N
O
N
N
CO2Me
O
OMe
23
F2 Chemical Shift (ppm)
F1 Chemical Shift (ppm)

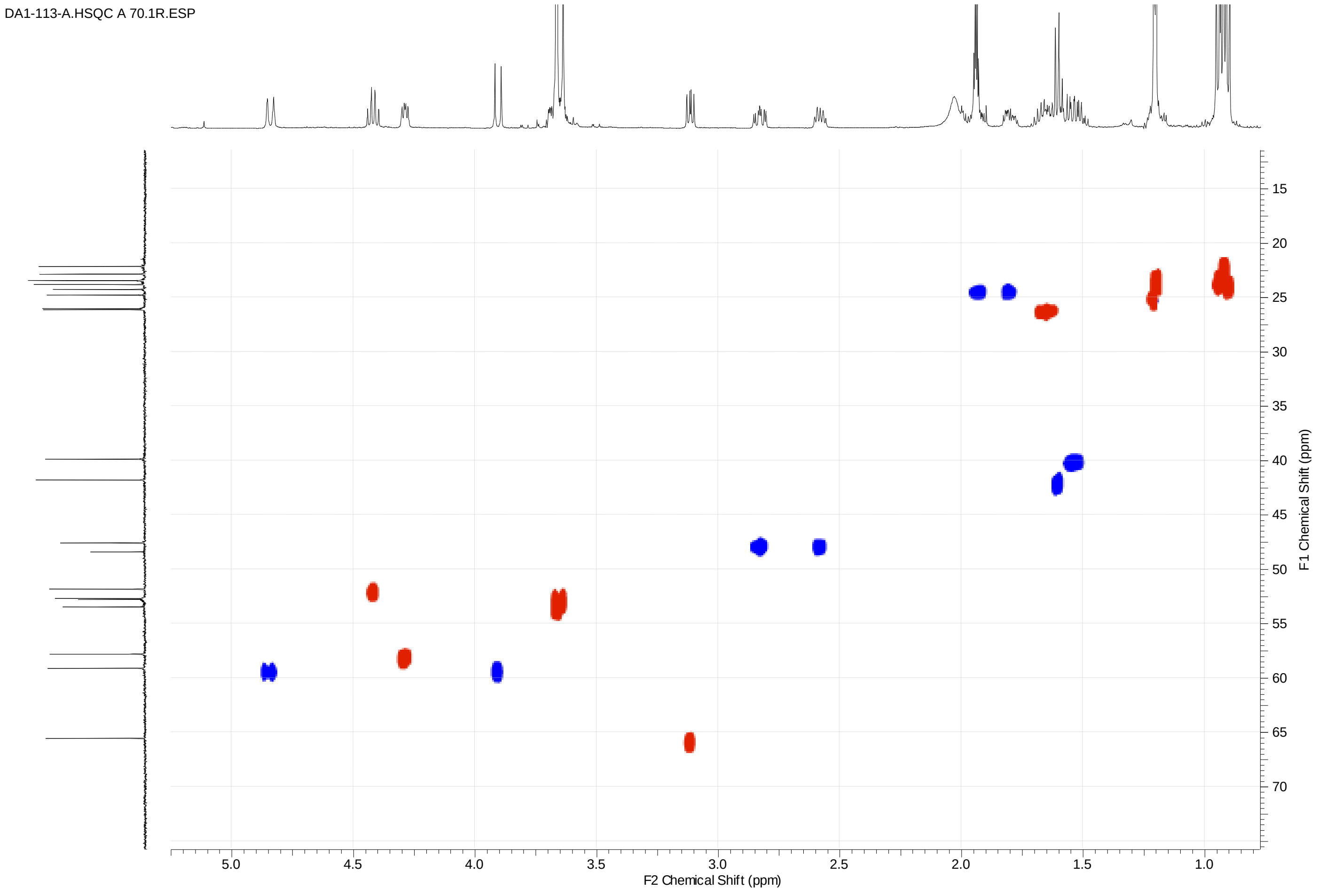
DA1-113-A.HSQC A 70.1R.ESP
F2 Chemical Shift (ppm)
F1 Chemical Shift (ppm)

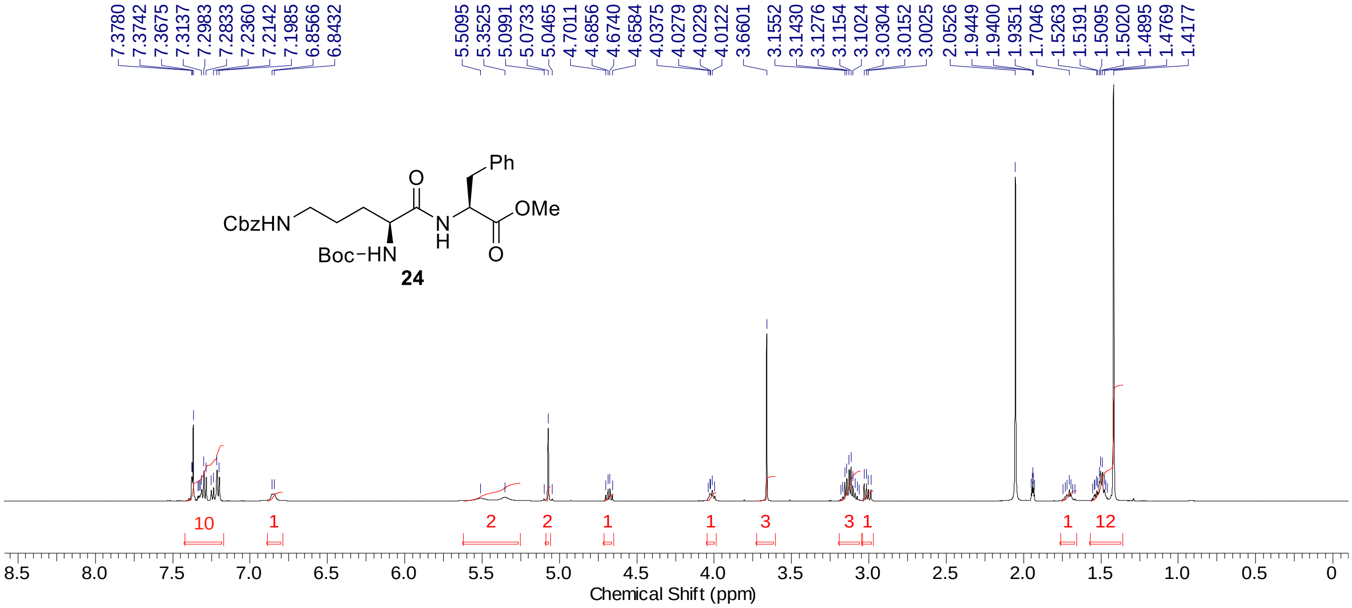

CbzHN
Boc−HN
O
N
H
Ph
OMe
O
7.3780 7.3742 7.3675 7.3137 7.2983 7.2833 7.2360 7.2142 7.1985 6.8566 6.8432
5.5095 5.3525 5.0991 5.0733 5.0465 4.7011 4.6856 4.6740 4.6584 4.0375 4.0279 4.0229 4.0122 3.6601
3.1552 3.1430 3.1276 3.1154 3.1024 3.0304 3.0152 3.0025 2.0526 1.9449 1.9400 1.9351 1.7046 1.5263 1.5191 1.5095 1.5020 1.4895 1.4769 1.4177
10 1 2 2 1 1 3 3 1 1 12
Chemical Shift (ppm)

24

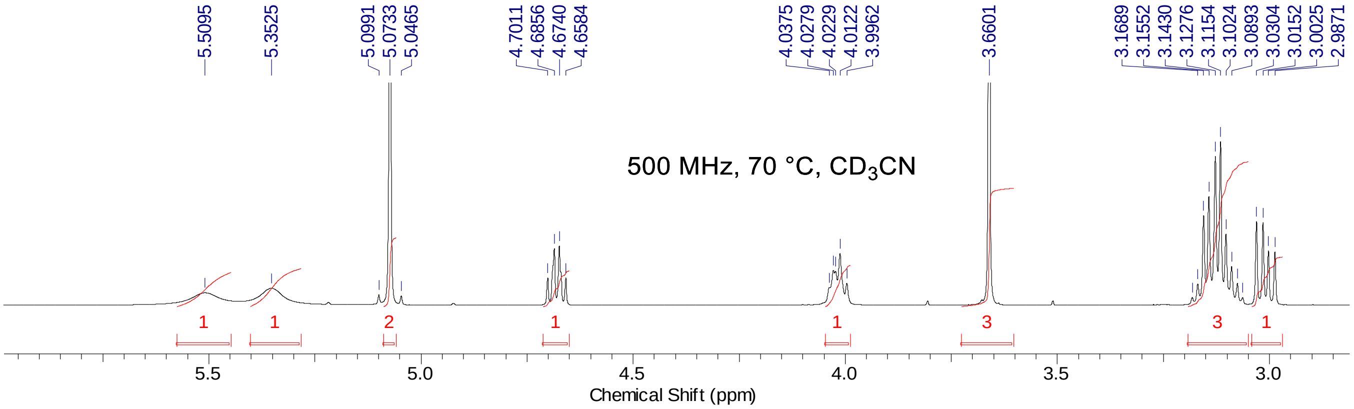

5.5095 5.3525 5.0991 5.0733 5.0465 4.7011 4.6856 4.6740 4.6584
4.0375 4.0279 4.0229 4.0122 3.9962 3.6601
3.1689 3.1552 3.1430 3.1276 3.1154 3.1024 3.0893 3.0304 3.0152 3.0025 2.9871
500 MHz, 70 °C, CD_3CN
1 1 2 1 1 3 3 1
Chemical Shift (ppm)

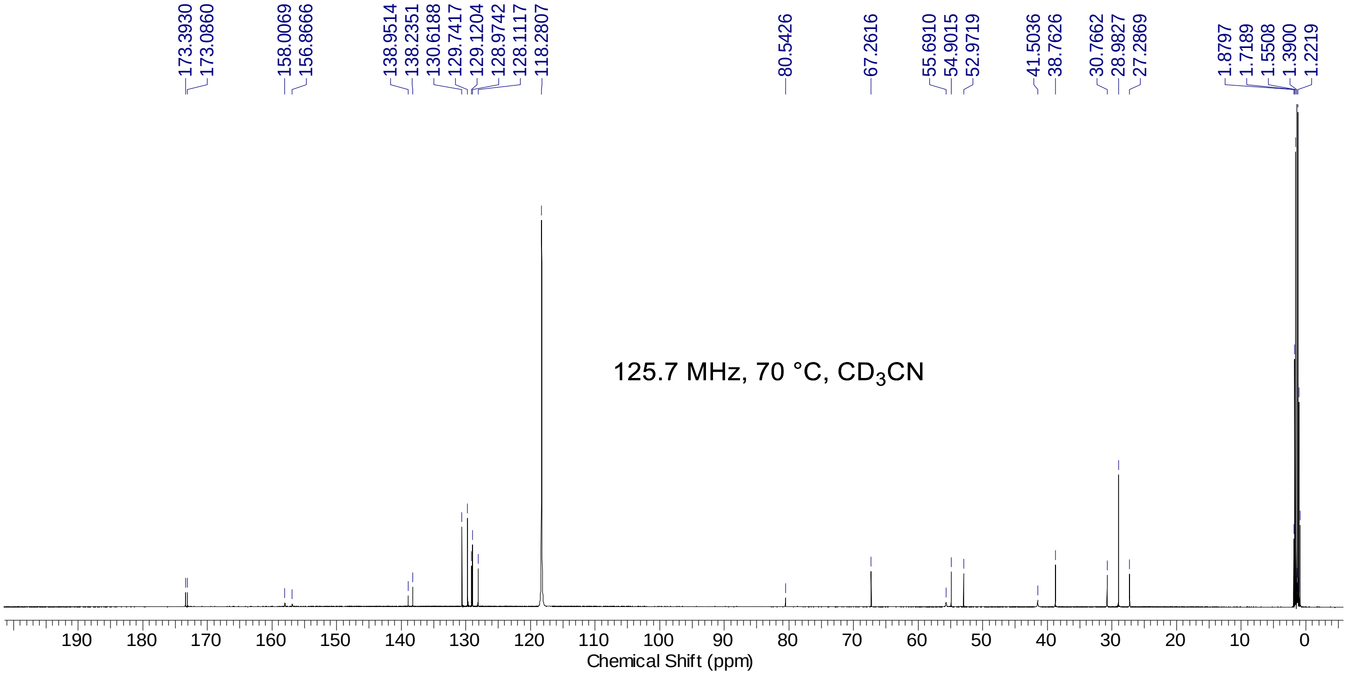

173.3930 173.0860 158.0069 156.8666 138.9514 138.2351 130.6188 129.7417 129.1204 128.9742 128.1117 118.2807
80.5426 67.2616 55.6910 54.9015 52.9719 41.5036 38.7626 30.7662 28.9827 27.2869
1.8797 1.7189 1.5508 1.3900 1.2219
125.7 MHz, 70 °C, CD_3CN
Chemical Shift (ppm)

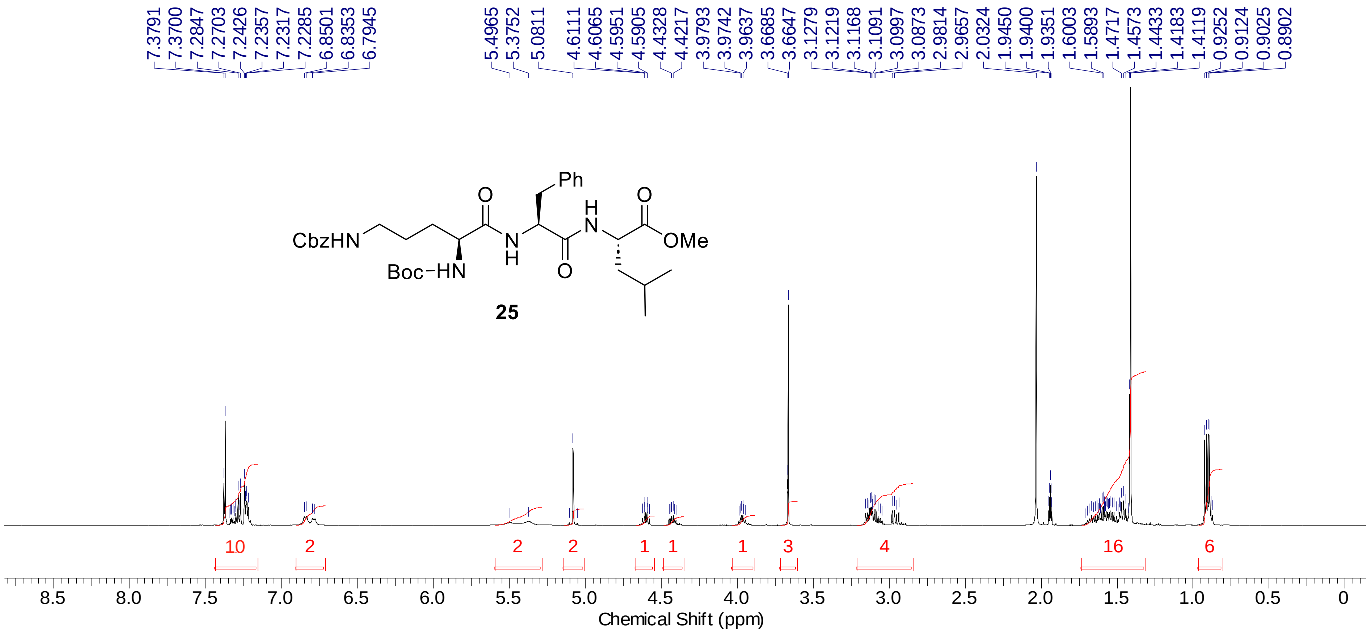
CbzHN
Boc–HN
Ph
OMe
25
Chemical Shift (ppm)

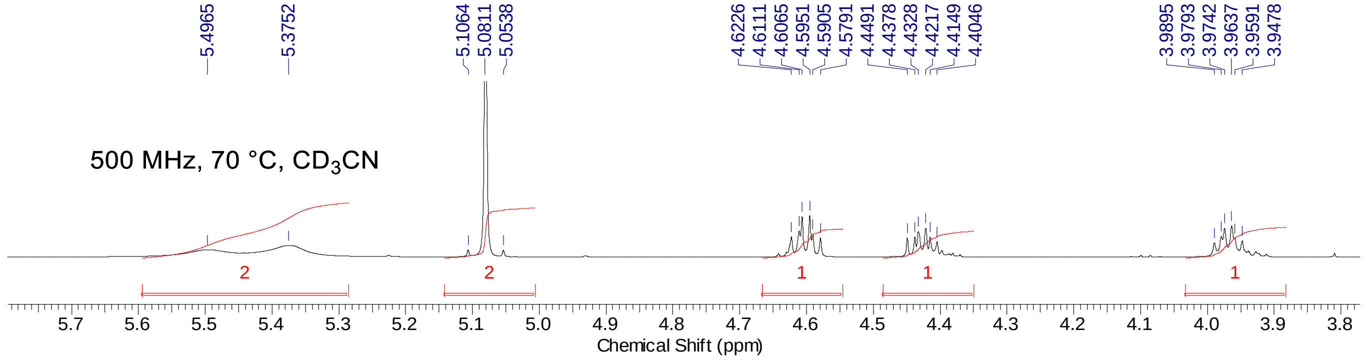
500 MHz, 70 °C, CD$_3$CN
Chemical Shift (ppm)

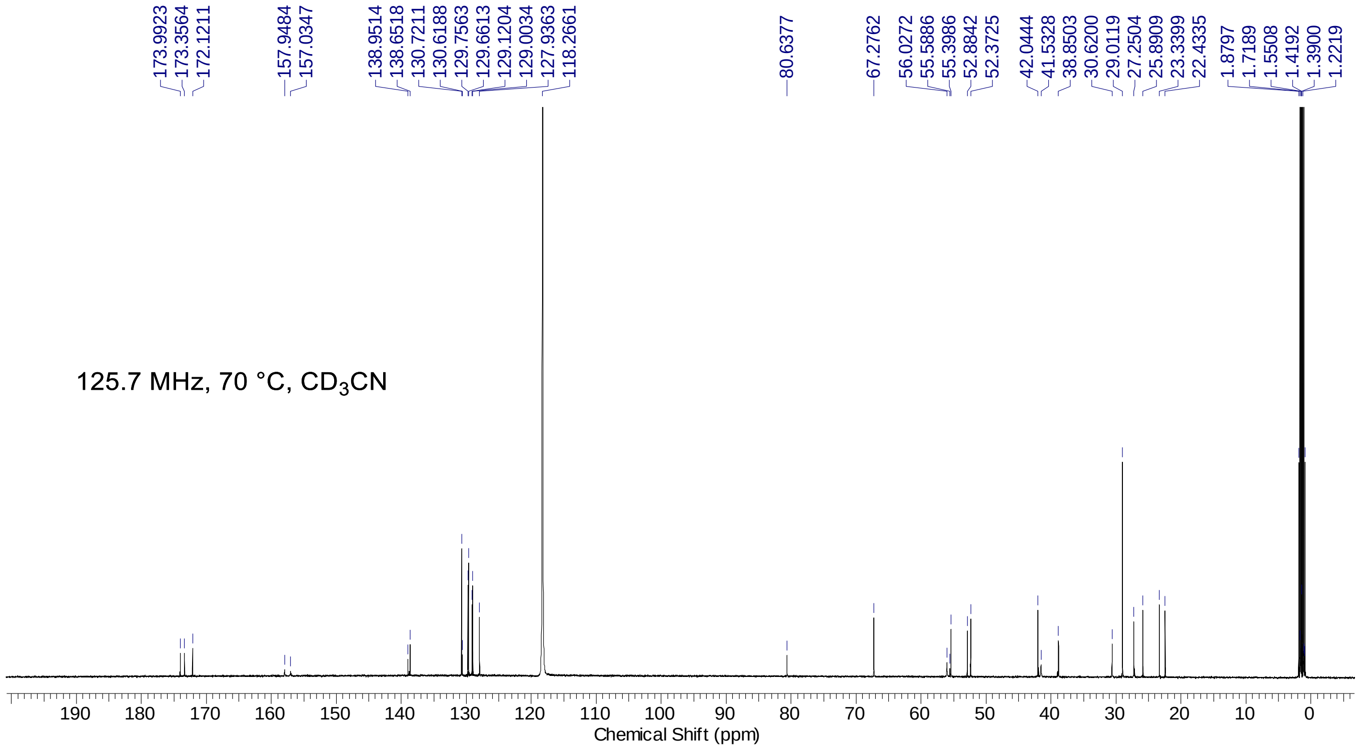
125.7 MHz, 70 °C, CD$_3$CN
Chemical Shift (ppm)

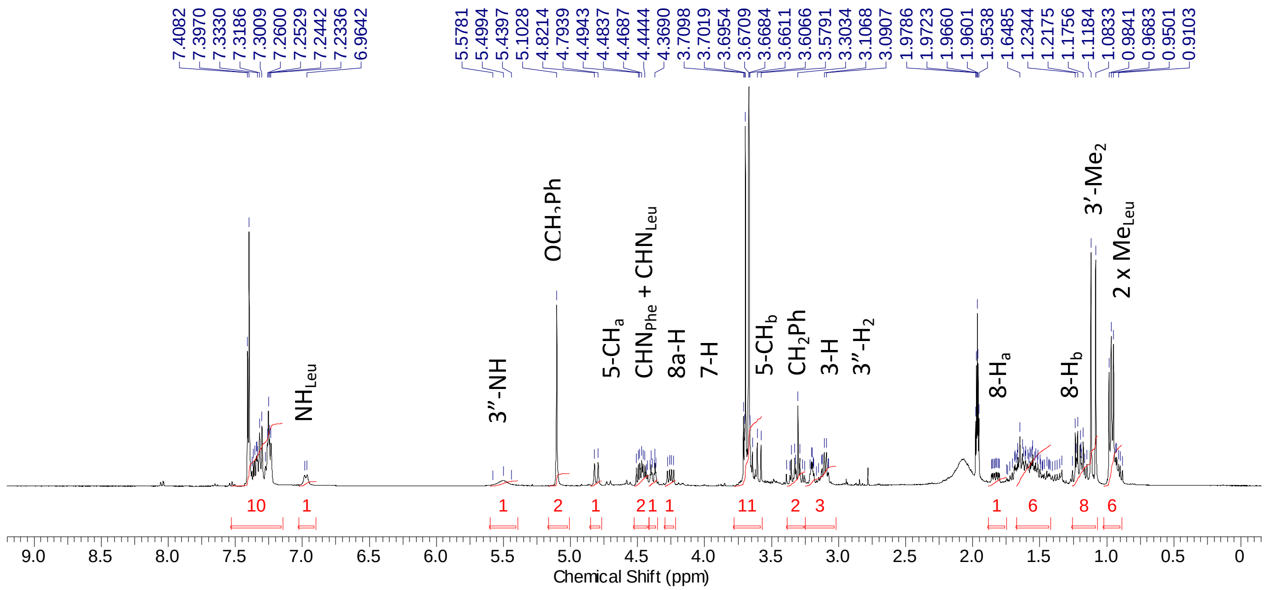
7.4082
7.3970
7.3330
7.3186
7.3009
7.2600
7.2529
7.2442
7.2336
6.9642
5.5781
5.4994
5.4397
5.1028
4.8214
4.7939
4.4943
4.4837
4.4687
4.4444
4.3690
3.7098
3.7019
3.6954
3.6709
3.6684
3.6611
3.6066
3.5791
3.3034
3.1068
3.0907
1.9786
1.9723
1.9660
1.9601
1.9538
1.6485
1.2344
1.2175
1.1756
1.1184
1.0833
0.9841
0.9683
0.9501
0.9103
NH_{Leu}
3''-NH
OCH_2Ph
5-CH_a
CHN_{Phe} + CHN_{Leu}
8a-H
7-H
5-CH_b
CH_2Ph
3-H
3''-H_2
8-H_a
8-H_b
3'-Me_2
2 x Me_{Leu}
10
1
1
2
1
2
1
1
11
2
3
1
6
8
6
Chemical Shift (ppm)

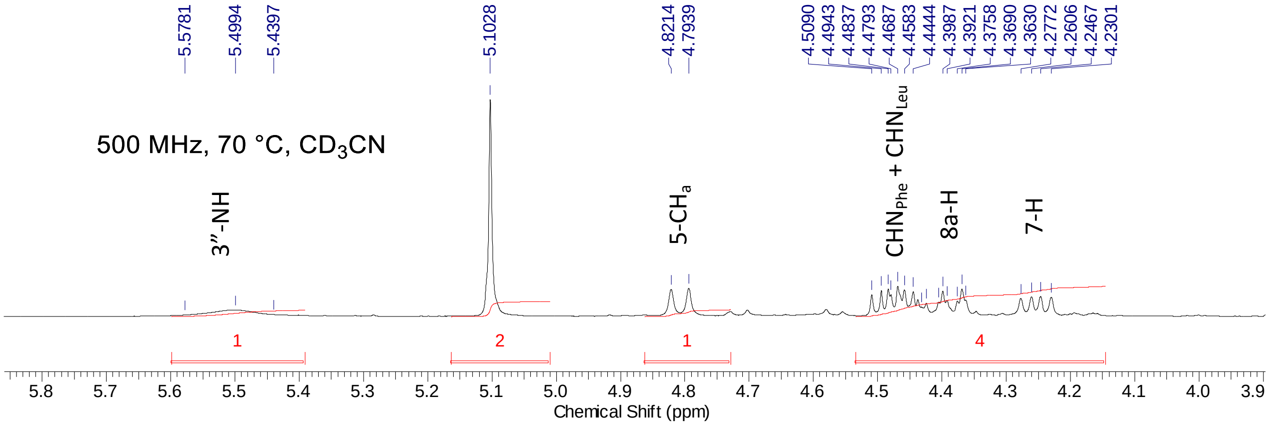
5.5781
5.4994
5.4397
5.1028
4.8214
4.7939
4.5090
4.4943
4.4837
4.4793
4.4687
4.4583
4.4444
4.3987
4.3921
4.3758
4.3690
4.3630
4.2772
4.2606
4.2467
4.2301
500 MHz, 70 °C, CD_3CN
3''-NH
5-CH_a
CHN_{Phe} + CHN_{Leu}
8a-H
7-H
1
2
1
4
Chemical Shift (ppm)

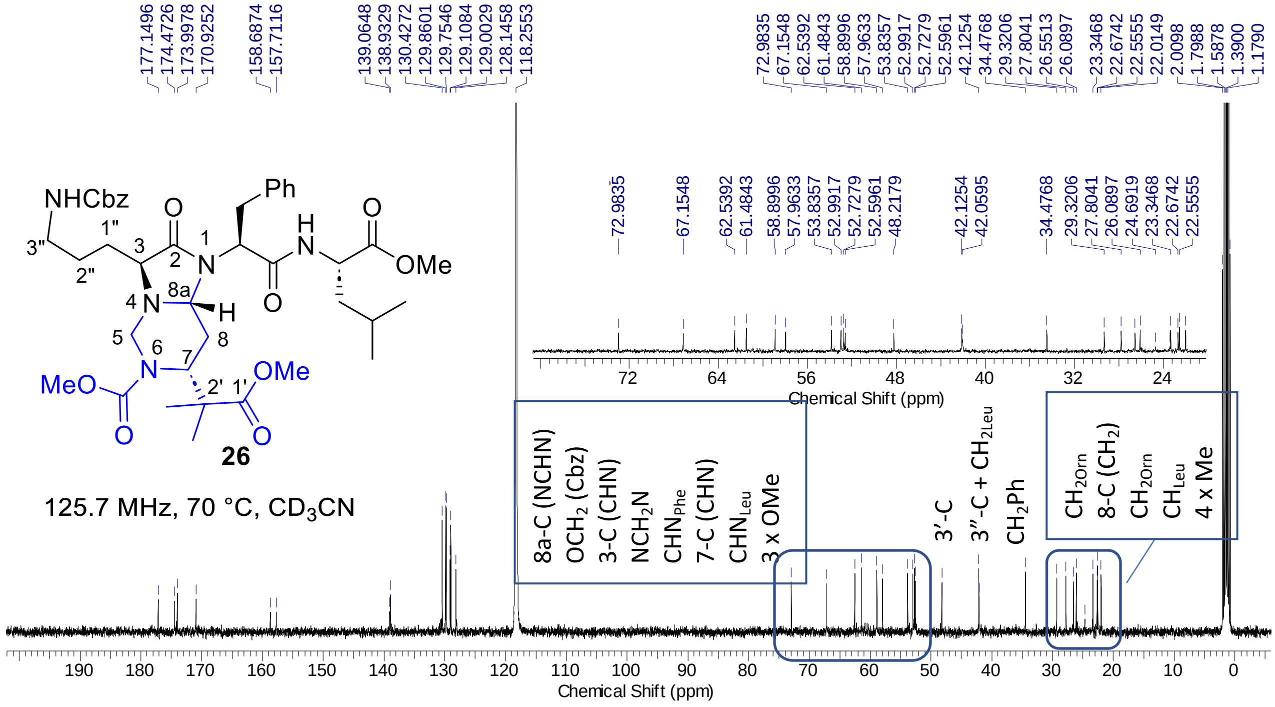
177.1496
174.4726
173.9978
170.9252
158.6874
157.7116
139.0648
138.9329
130.4272
129.8601
129.7546
129.1084
129.0029
128.1458
118.2553
72.9835
67.1548
62.5392
61.4843
58.8996
57.9633
53.8357
52.9917
52.7279
52.5961
42.1254
34.4768
29.3206
27.8041
26.5513
26.0897
23.3468
22.6742
22.5555
22.0149
2.0098
1.7988
1.5878
1.3900
1.1790
48.2179
42.0595
24.6919
26
125.7 MHz, 70 °C, CD_3CN
8a-C (NCHN)
OCH_2 (Cbz)
3-C (CHN)
NCH_2N
CHN_{Phe}
7-C (CHN)
CHN_{Leu}
3 x OMe
3'-C
3''-C + CH_{2Leu}
CH_2Ph
CH_{2Orn}
8-C (CH_2)
CH_{2Orn}
CH_{Leu}
4 x Me
Chemical Shift (ppm)

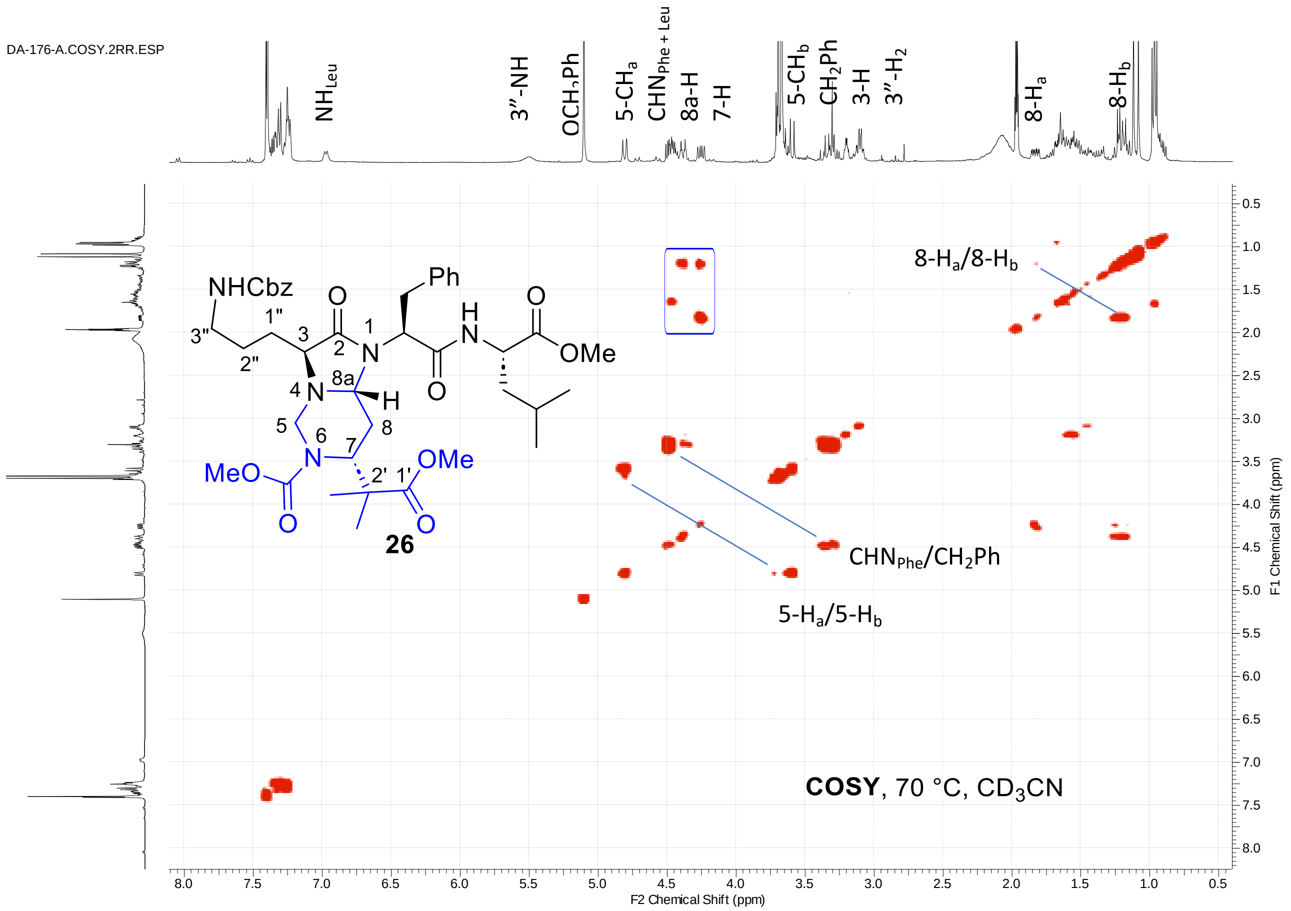
DA-176-A.COSY.2RR.ESP
NH$_{Leu}$
3''-NH
OCH$_2$Ph
5-CH$_a$
CHN$_{Phe + Leu}$
8a-H
7-H
5-CH$_b$
CH$_2$Ph
3-H
3''-H$_2$
8-H$_a$
8-H$_b$
8-H$_a$/8-H$_b$
CHN$_{Phe}$/CH$_2$Ph
5-H$_a$/5-H$_b$
NHCbz
Ph
OMe
MeO
26
COSY, 70 °C, CD$_3$CN
F2 Chemical Shift (ppm)
F1 Chemical Shift (ppm)

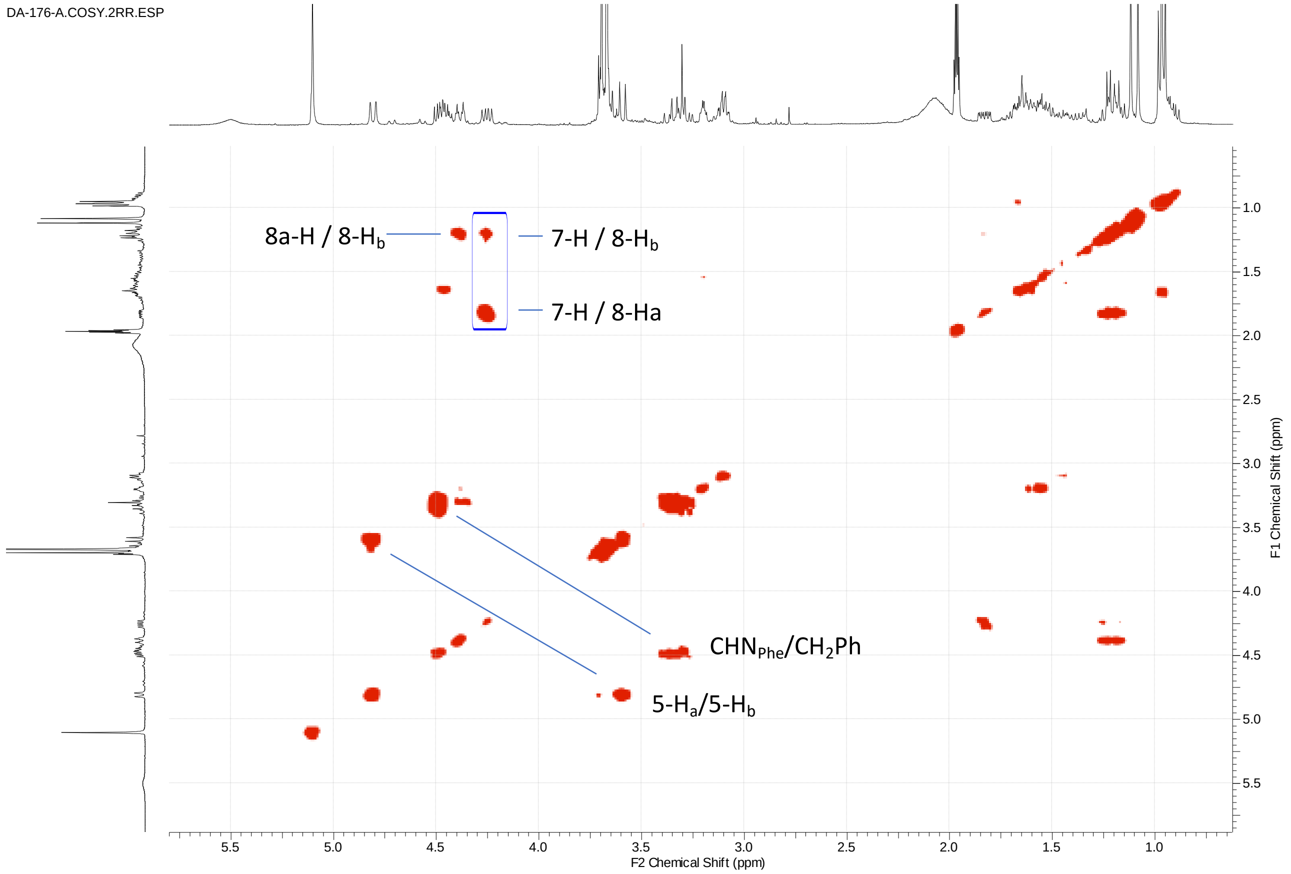
DA-176-A.COSY.2RR.ESP
8a-H / 8-H$_b$
7-H / 8-H$_b$
7-H / 8-Ha
CHN$_{Phe}$/CH$_2$Ph
5-H$_a$/5-H$_b$
F2 Chemical Shift (ppm)
F1 Chemical Shift (ppm)

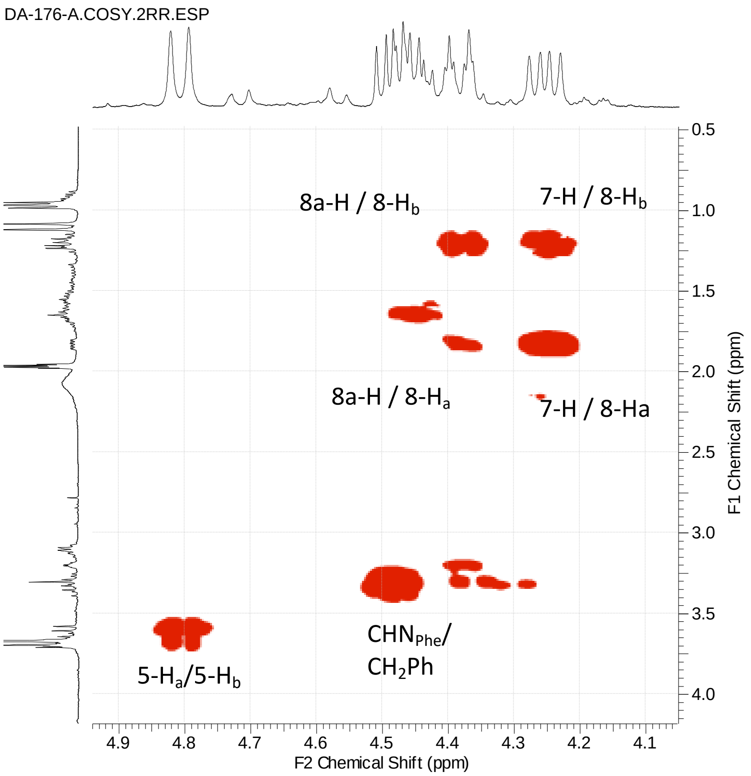

COSY (deeper layers)

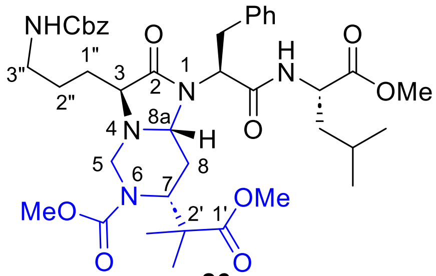

26

COSY, 70 °C, CD_3CN

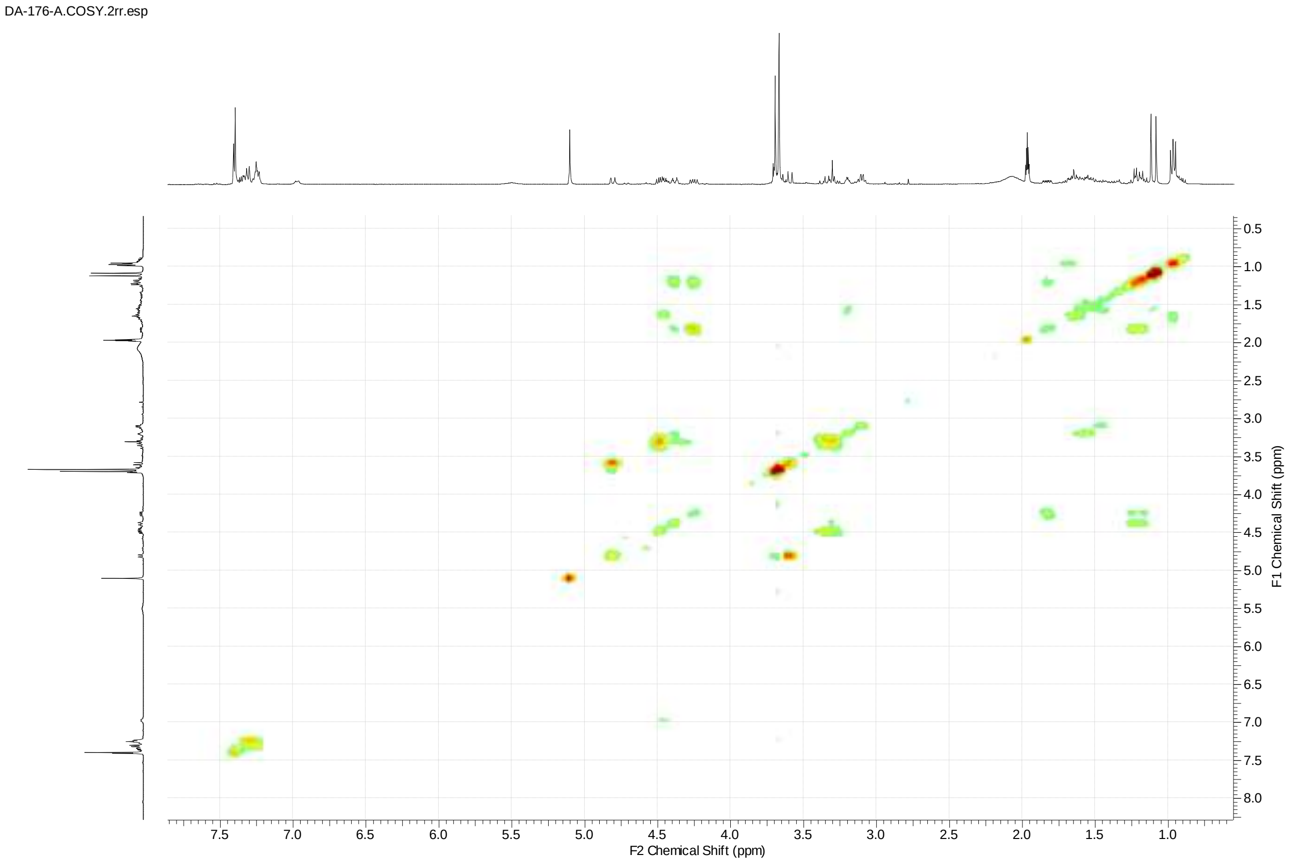

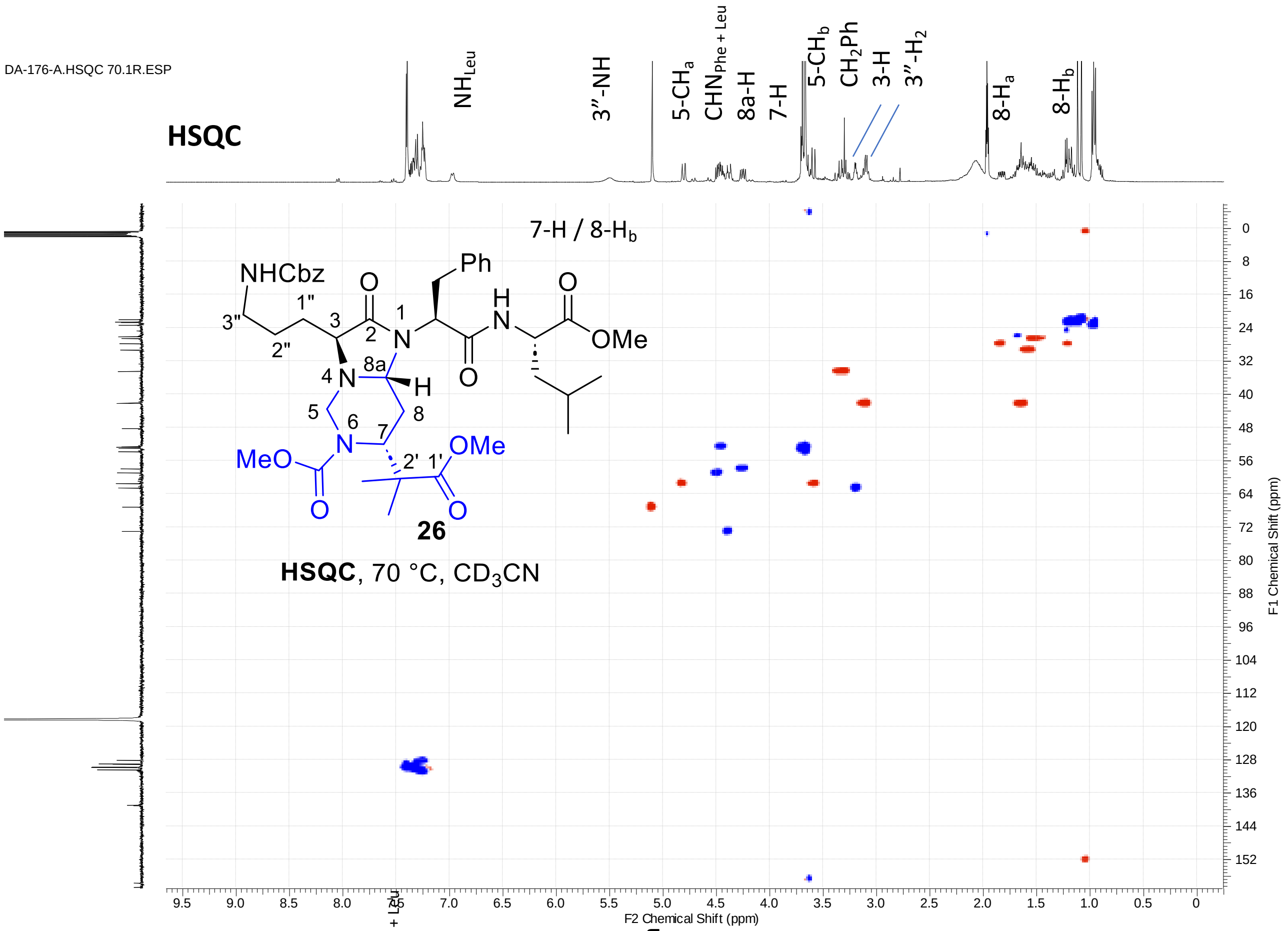
DA-176-A.HSQC 70.1R.ESP
HSQC
7-H / 8-H$_b$
26
HSQC, 70 °C, CD$_3$CN
F2 Chemical Shift (ppm)
F1 Chemical Shift (ppm)

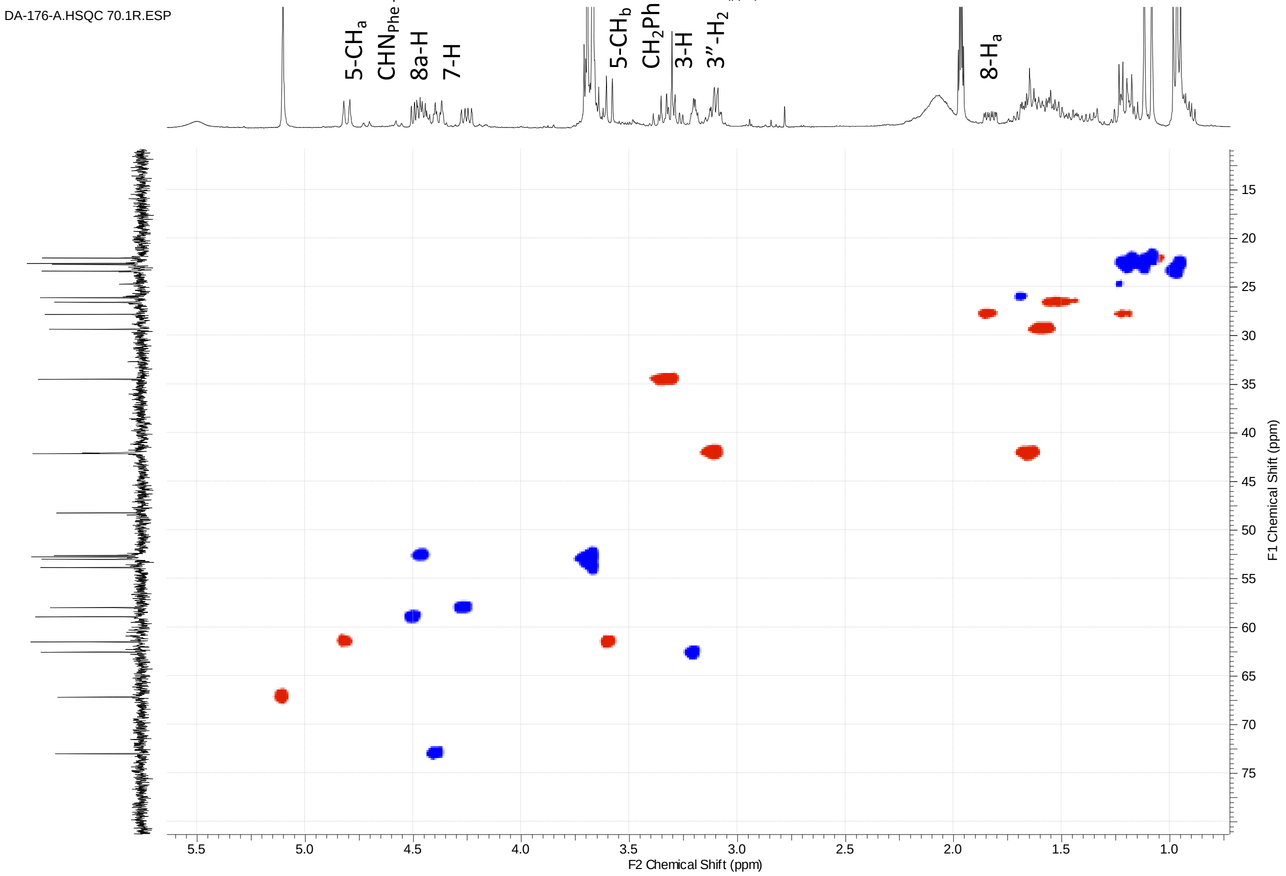
DA-176-A.HSQC 70.1R.ESP
F2 Chemical Shift (ppm)
F1 Chemical Shift (ppm)

DA-176-A.HMBC.2RR.ESP

HMBC

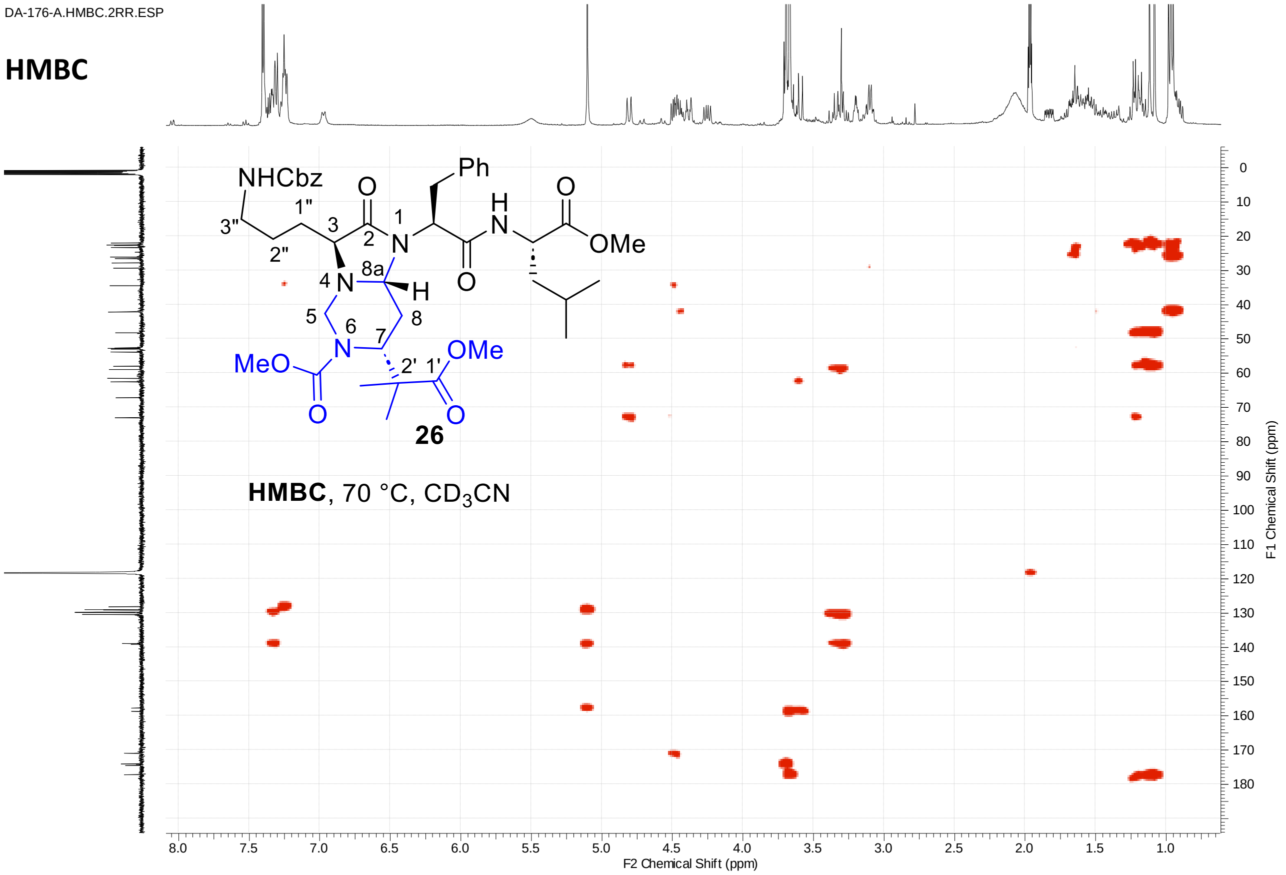

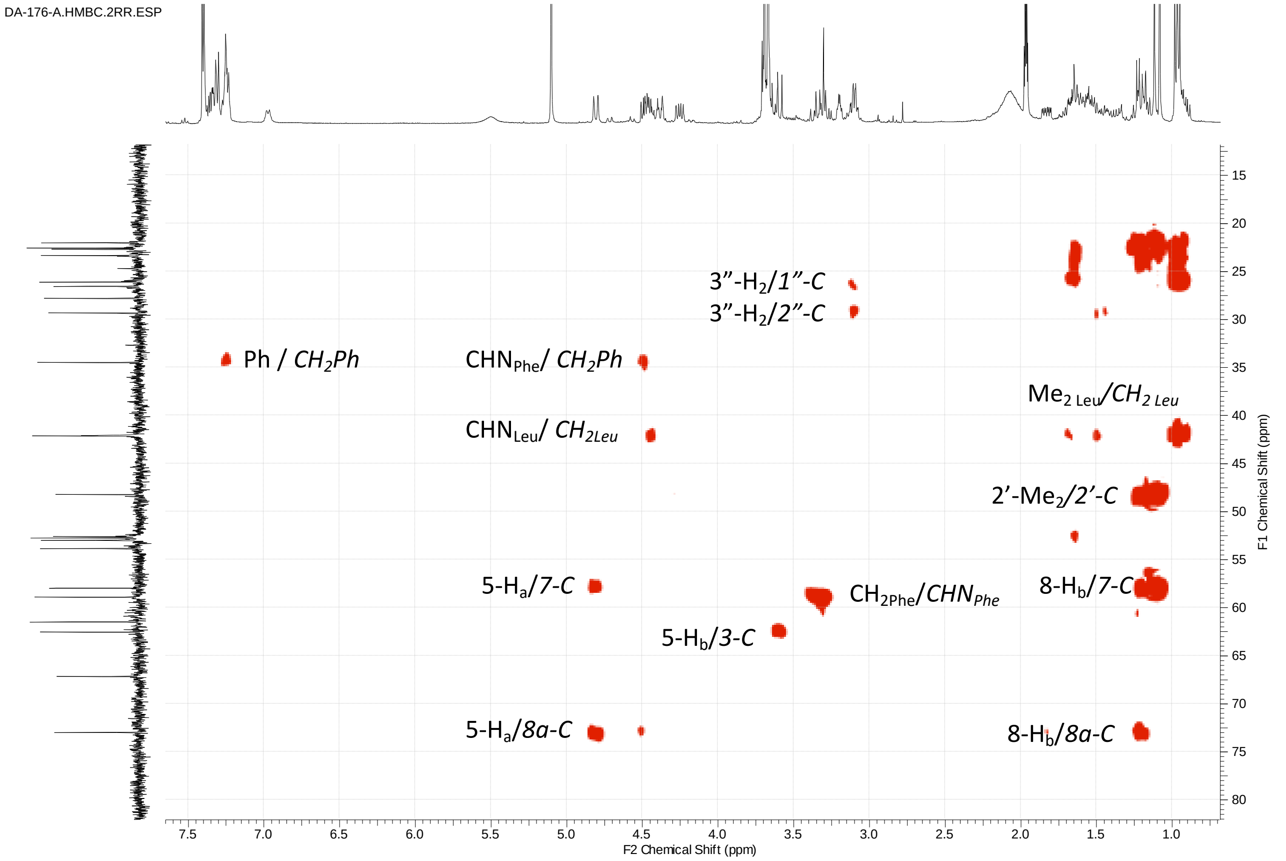

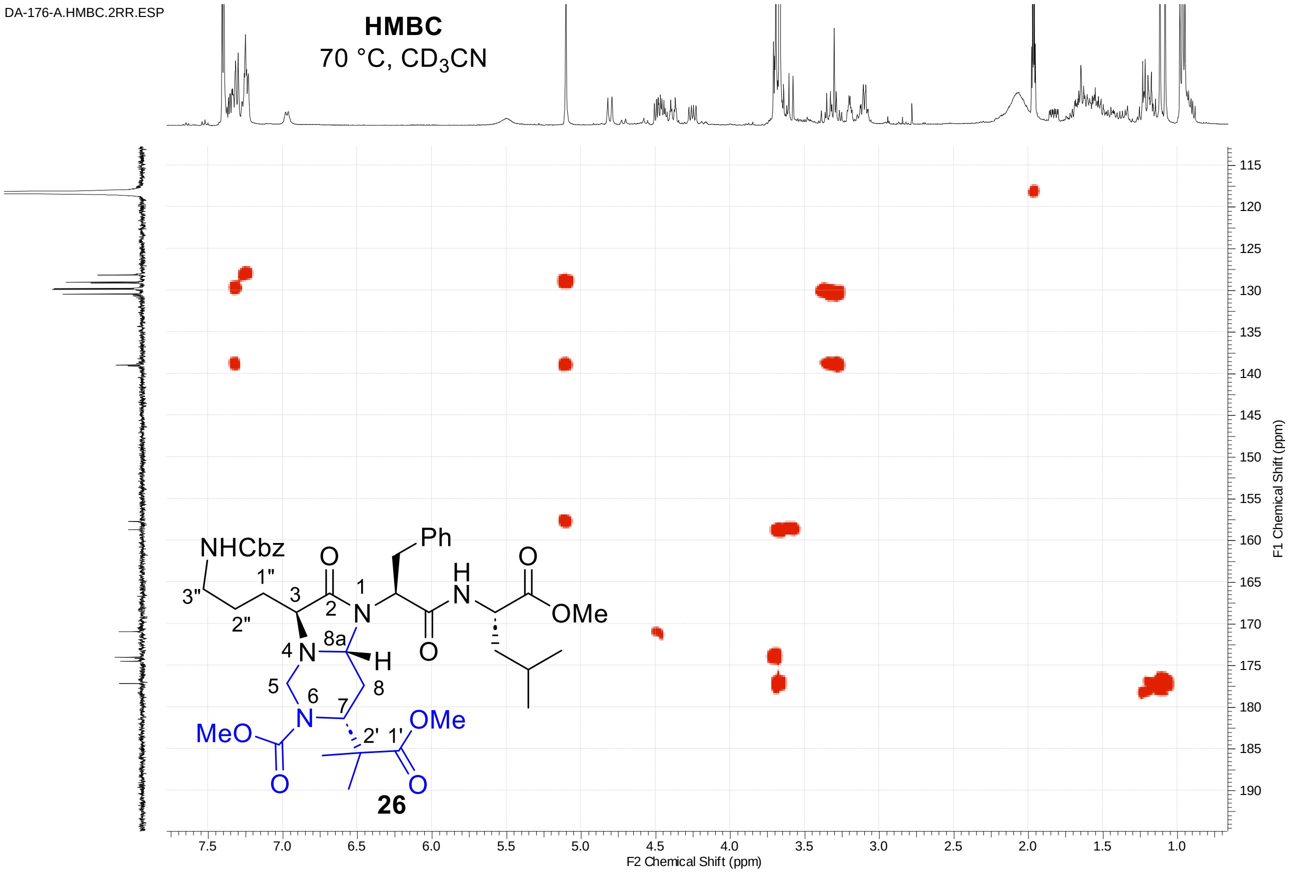
DA-176-A.HMBC.2RR.ESP
HMBC
70 °C, CD_3CN
NHCbz
Ph
OMe
MeO
26
F2 Chemical Shift (ppm)
F1 Chemical Shift (ppm)

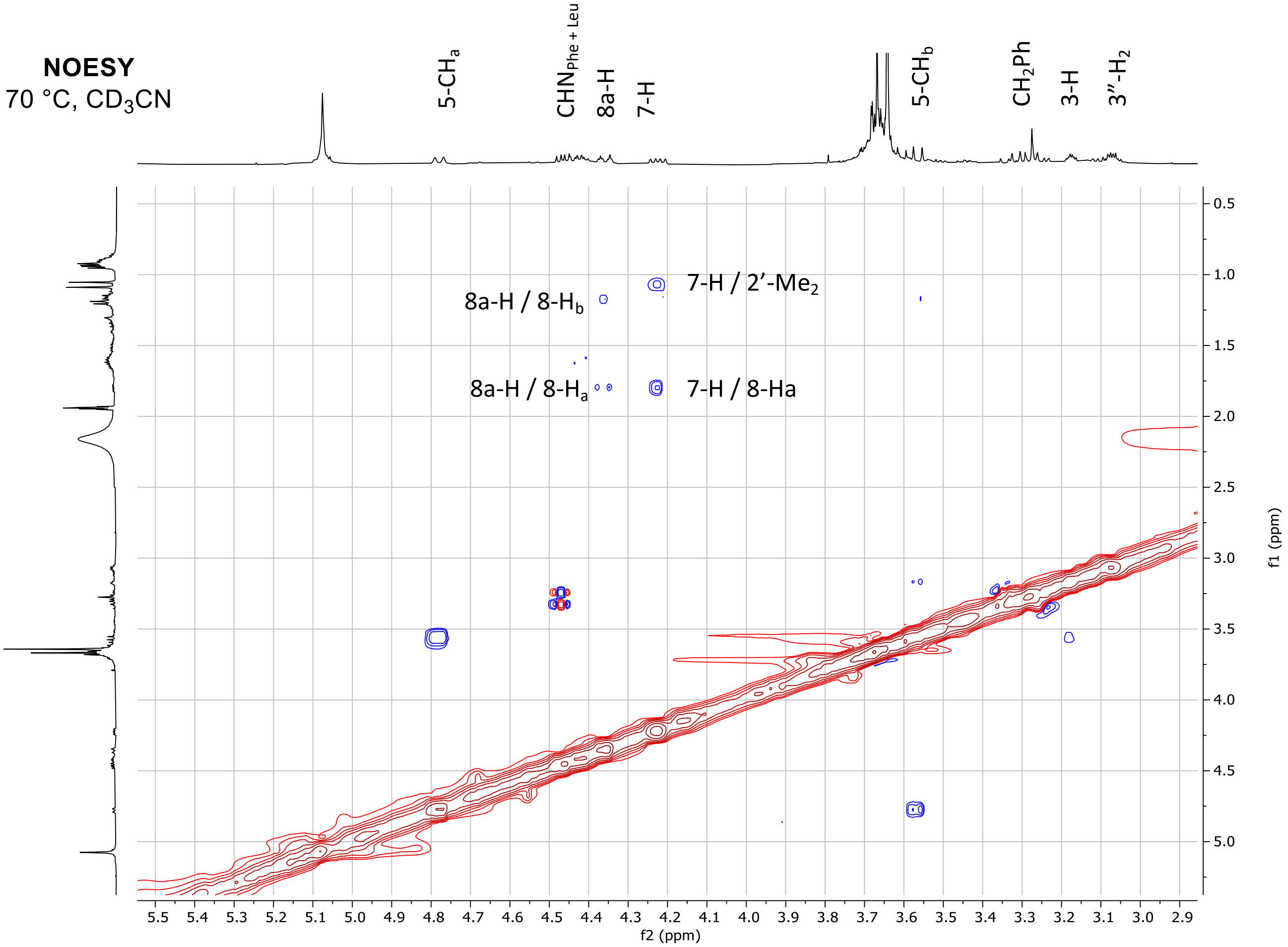
NOESY
70 °C, CD_3CN
5-CH_a
$CHN_{Phe + Leu}$
8a-H
7-H
5-CH_b
CH_2Ph
3-H
3''-H_2
8a-H / 8-H_b
7-H / 2'-Me_2
8a-H / 8-H_a
7-H / 8-Ha
f2 (ppm)
f1 (ppm)

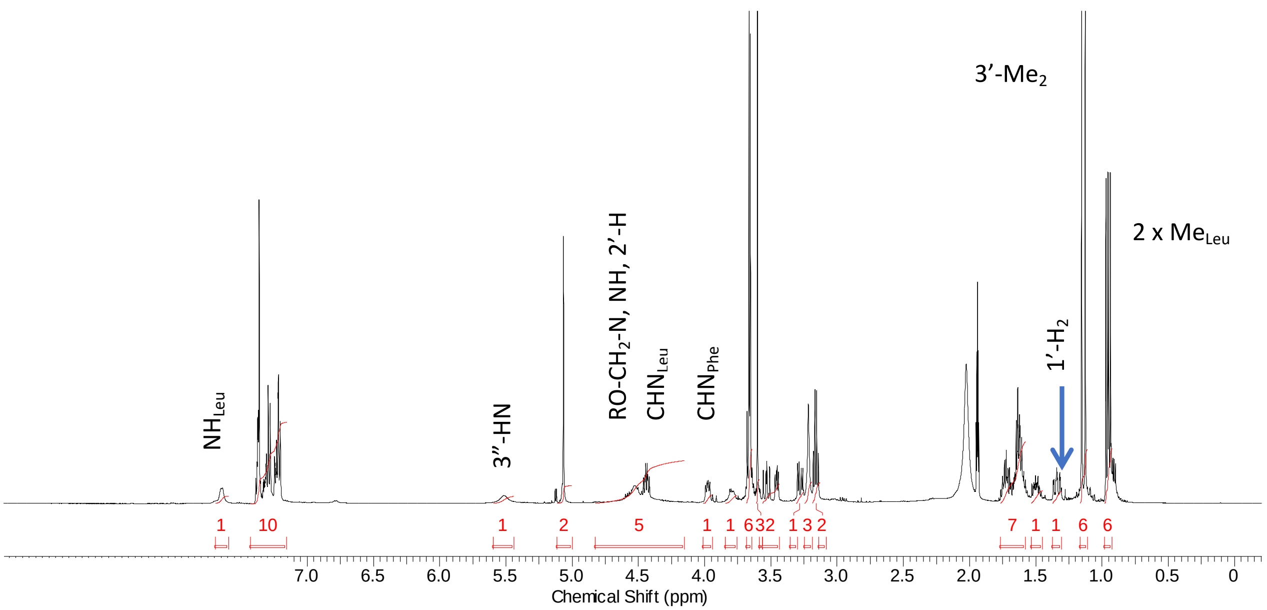

3'-Me2
2 x MeLeu
NHLeu
3''-HN
RO-CH2-N, NH, 2'-H
CHNLeu
CHNPhe
1'-H2
Chemical Shift (ppm)

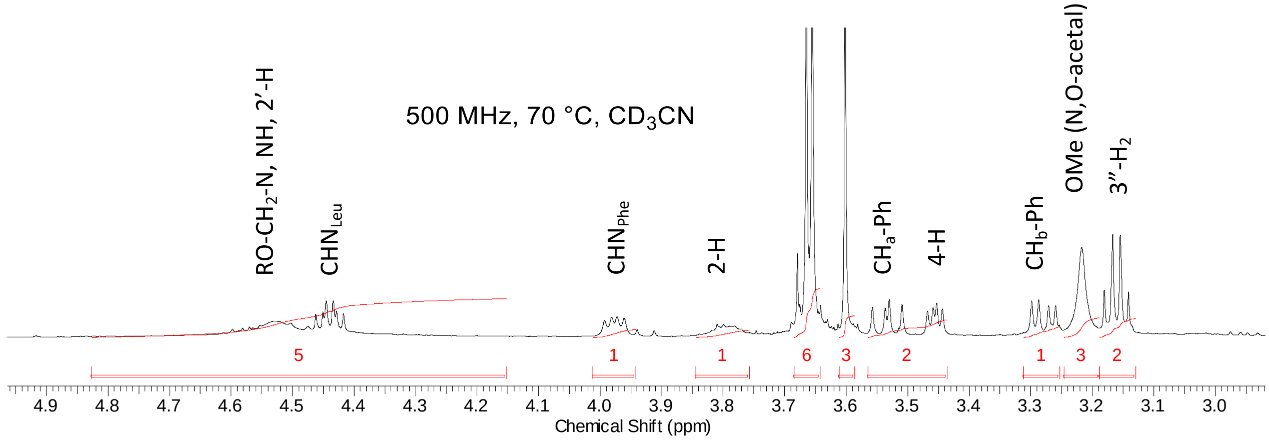

500 MHz, 70 °C, CD3CN
RO-CH2-N, NH, 2'-H
CHNLeu
CHNPhe
2-H
CHa-Ph
4-H
CHb-Ph
OMe (N,O-acetal)
3''-H2
Chemical Shift (ppm)

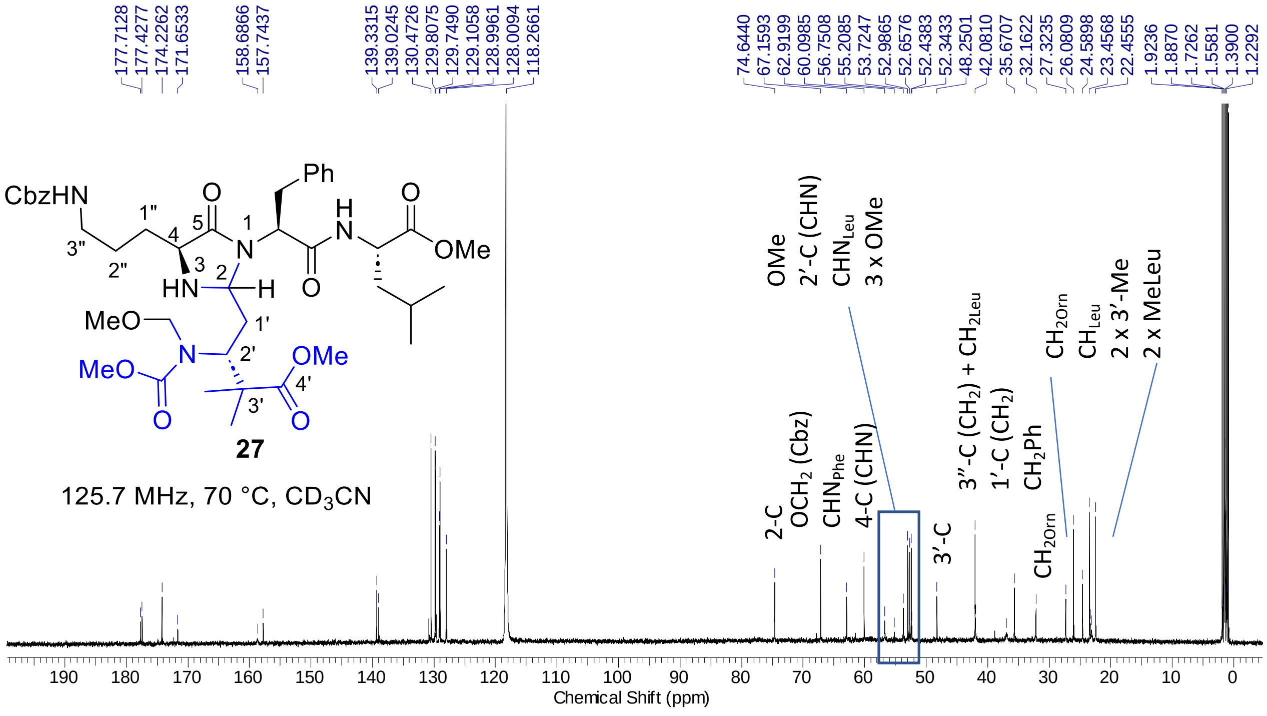

177.7128
177.4277
174.2262
171.6533
158.6866
157.7437
139.3315
139.0245
130.4726
129.8075
129.7490
129.1058
128.9961
128.0094
118.2661
74.6440
67.1593
62.9199
60.0985
56.7508
55.2085
53.7247
52.9865
52.6576
52.4383
52.3433
48.2501
42.0810
35.6707
32.1622
27.3235
26.0809
24.5898
23.4568
22.4555
1.9236
1.8870
1.7262
1.5581
1.3900
1.2292
CbzHN
Ph
OMe
MeO
27
125.7 MHz, 70 °C, CD3CN
OMe
2'-C (CHN)
CHNLeu
3 x OMe
2-C
OCH2 (Cbz)
CHNPhe
4-C (CHN)
3'-C
3''-C (CH2) + CH2Leu
1'-C (CH2)
CH2Ph
CH2Orn
CH2Orn
CHLeu
2 x 3'-Me
2 x MeLeu
Chemical Shift (ppm)

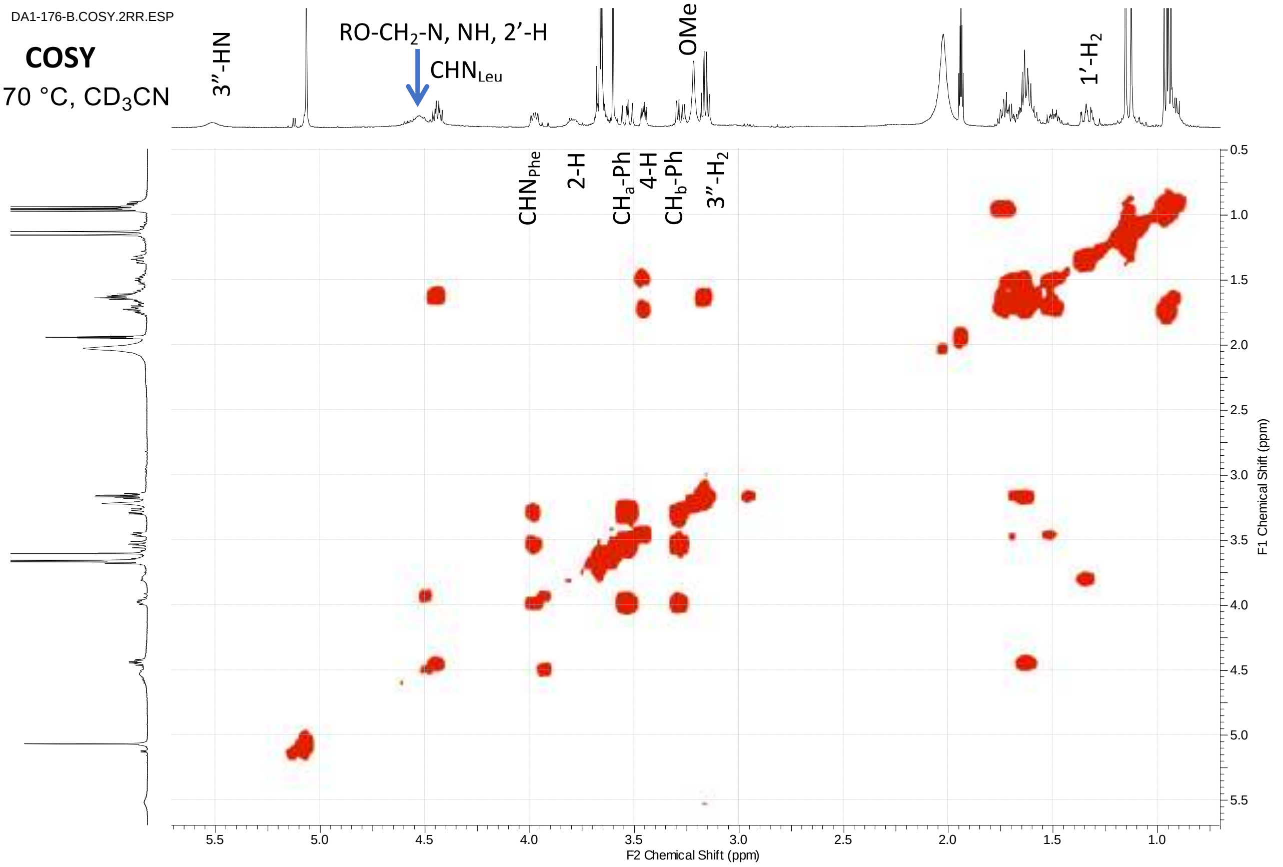
DA1-176-B.COSY.2RR.ESP
COSY
70 °C, CD3CN
3''-HN
RO-CH2-N, NH, 2'-H
CHNLeu
OMe
1'-H2
CHNPhe
2-H
CHa-Ph
4-H
CHb-Ph
3''-H2
F1 Chemical Shift (ppm)
F2 Chemical Shift (ppm)

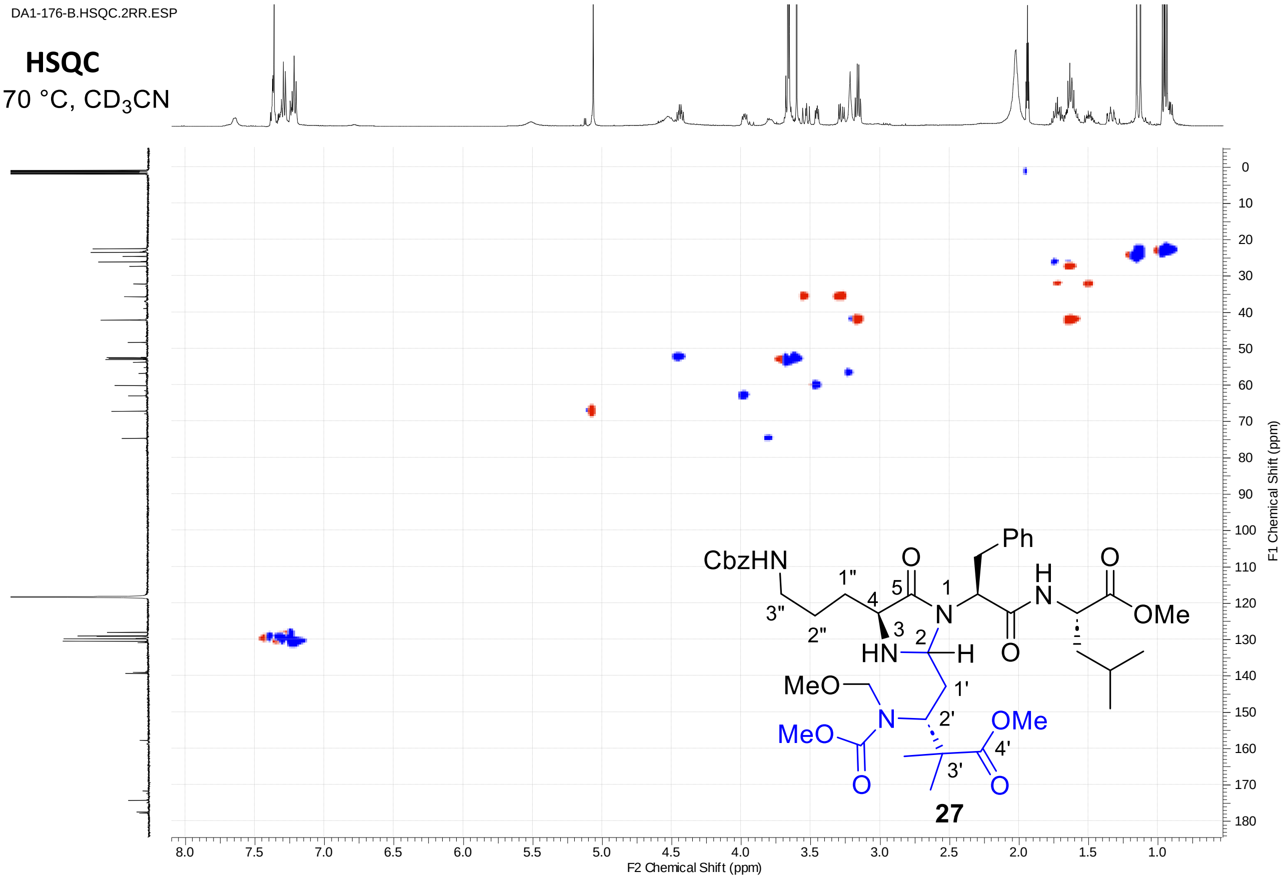
DA1-176-B.HSQC.2RR.ESP
HSQC
70 °C, CD3CN
F1 Chemical Shift (ppm)
F2 Chemical Shift (ppm)
CbzHN
Ph
OMe
MeO
27

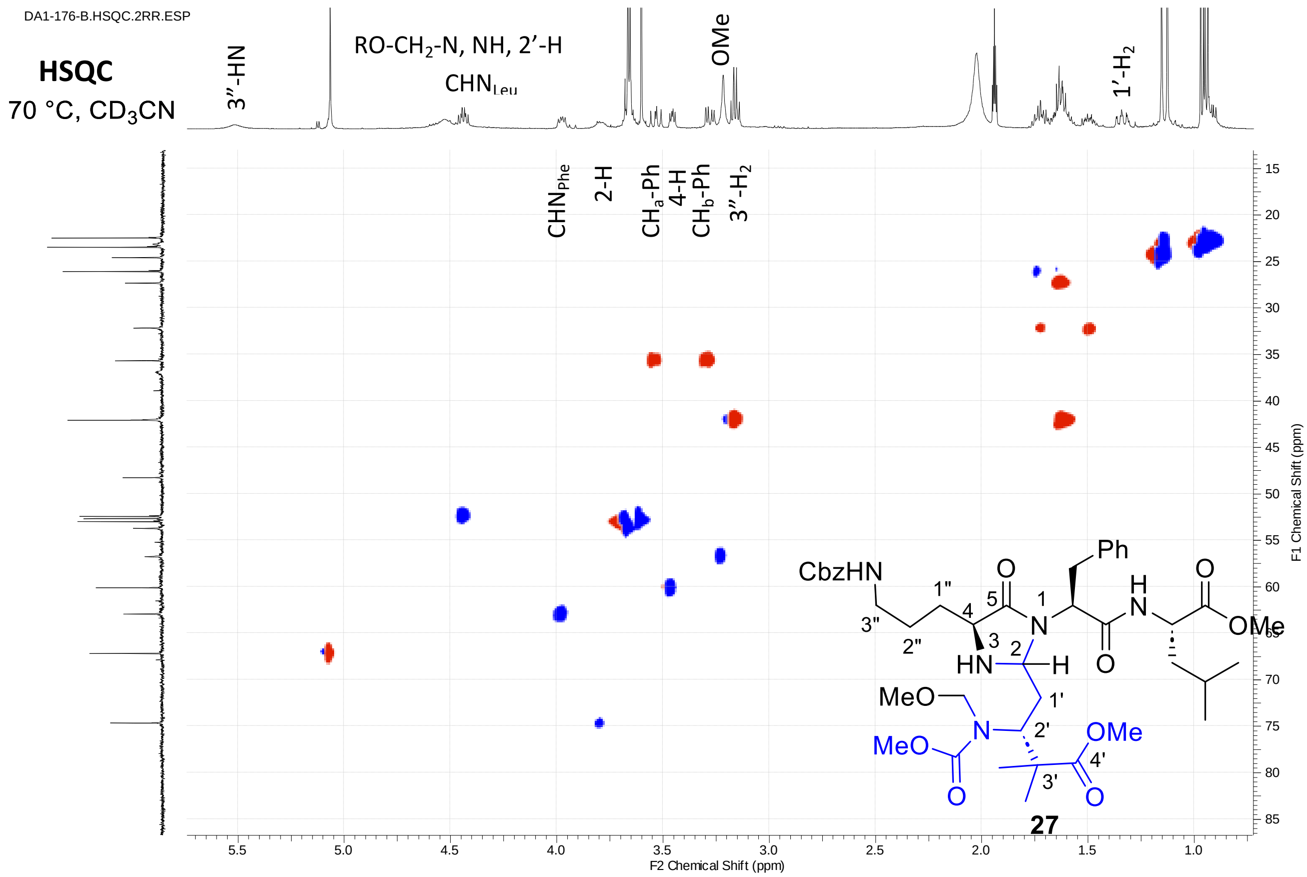
DA1-176-B.HSQC.2RR.ESP
HSQC
70 °C, CD3CN
3″-HN
RO-CH2-N, NH, 2′-H
CHNLeu
OMe
1′-H2
CHNPhe
2-H
CHa-Ph
4-H
CHb-Ph
3″-H2
CbzHN
Ph
27
F2 Chemical Shift (ppm)
F1 Chemical Shift (ppm)

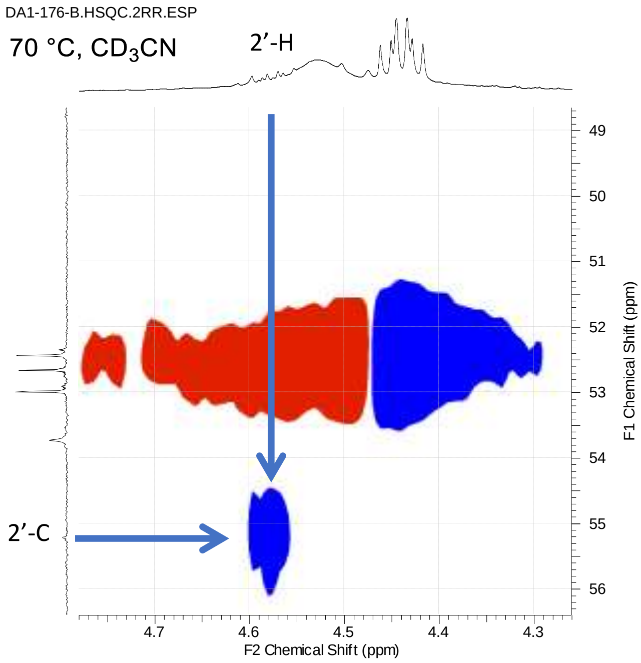
DA1-176-B.HSQC.2RR.ESP
70 °C, CD3CN
2′-H
2′-C
F2 Chemical Shift (ppm)
F1 Chemical Shift (ppm)

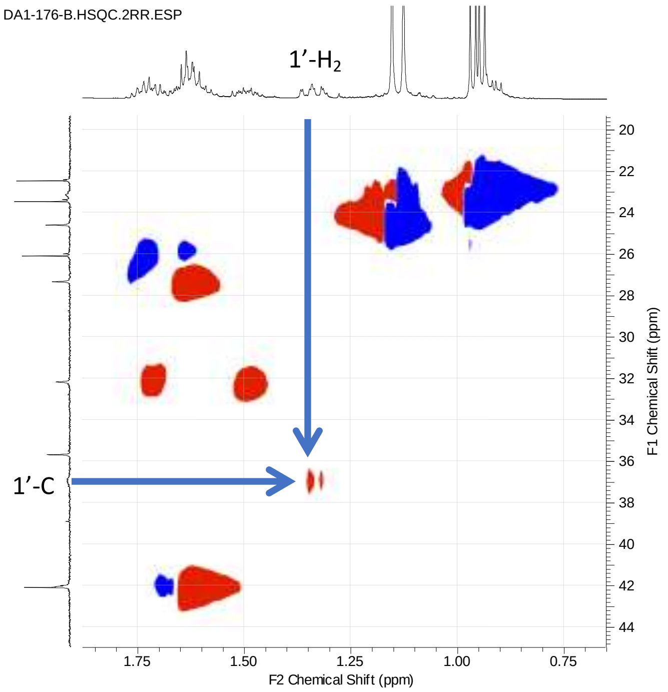
DA1-176-B.HSQC.2RR.ESP
1′-H2
1′-C
F2 Chemical Shift (ppm)
F1 Chemical Shift (ppm)

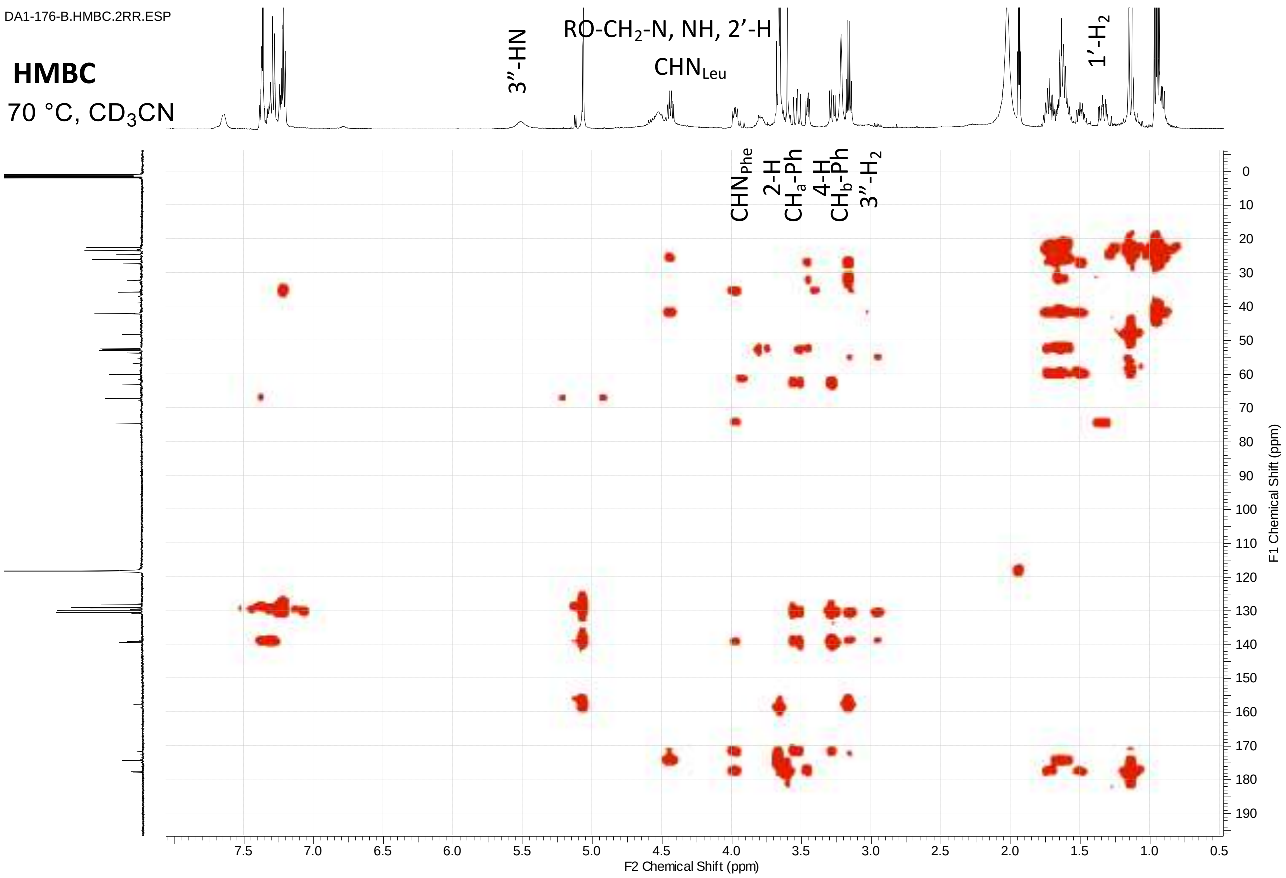
DA1-176-B.HMBC.2RR.ESP
HMBC
70 °C, CD3CN
3''-HN
RO-CH2-N, NH, 2'-H
CHNLeu
1'-H2
CHNPhe
2-H
CHa-Ph
4-H
CHb-Ph
3''-H2
F2 Chemical Shift (ppm)
F1 Chemical Shift (ppm)

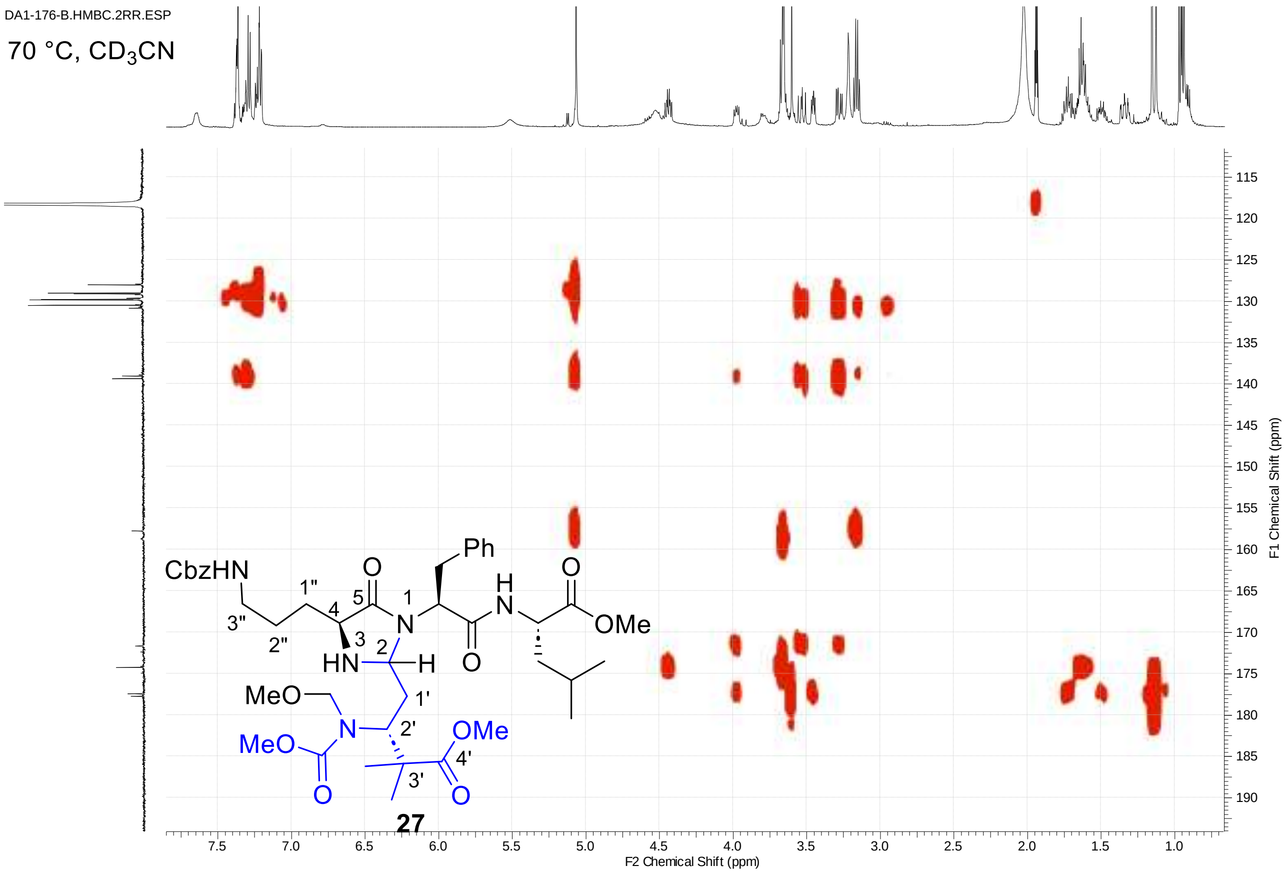
DA1-176-B.HMBC.2RR.ESP
70 °C, CD3CN
CbzHN
Ph
1''
3''
2''
4
5
1
3
2
HN
N
H
O
OMe
1'
2'
3'
4'
MeO
N
MeO
OMe
27
F2 Chemical Shift (ppm)
F1 Chemical Shift (ppm)

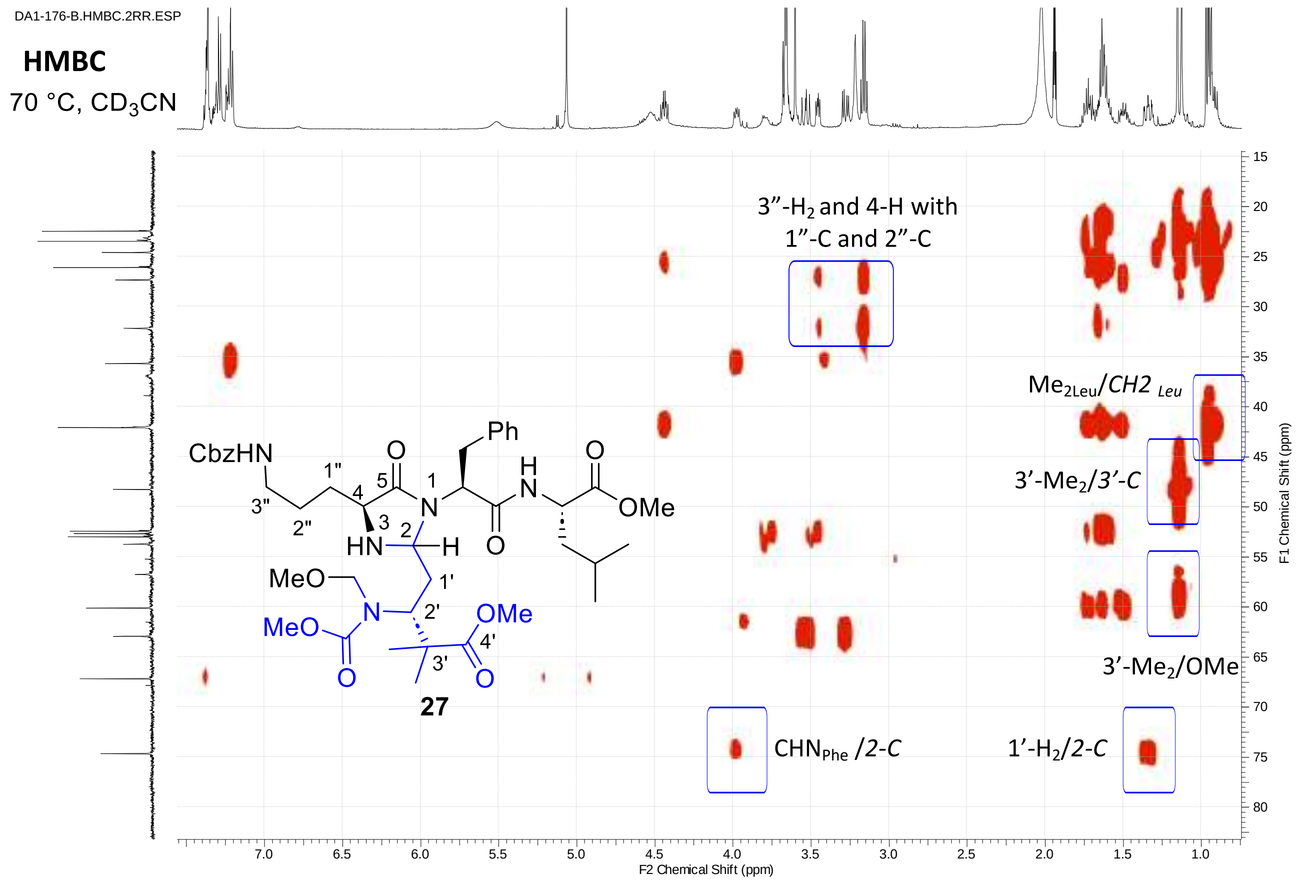

At deeper layers:

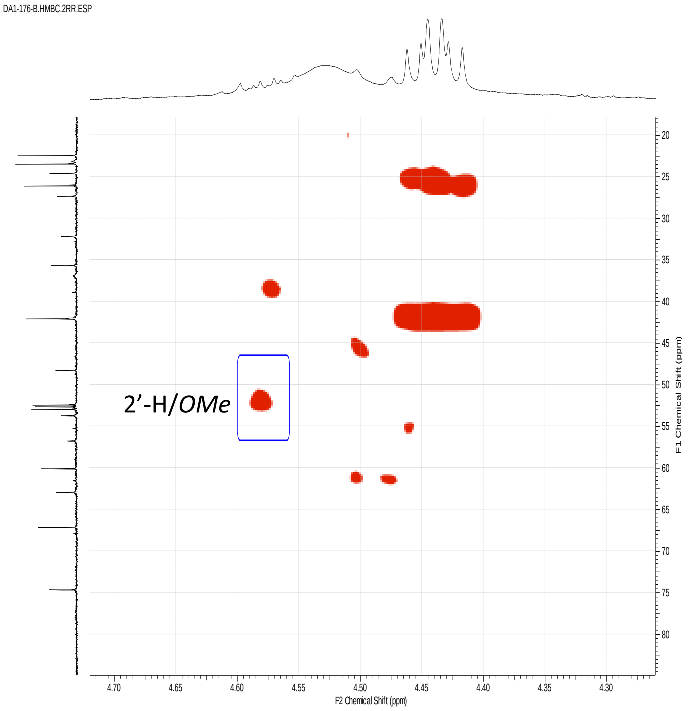

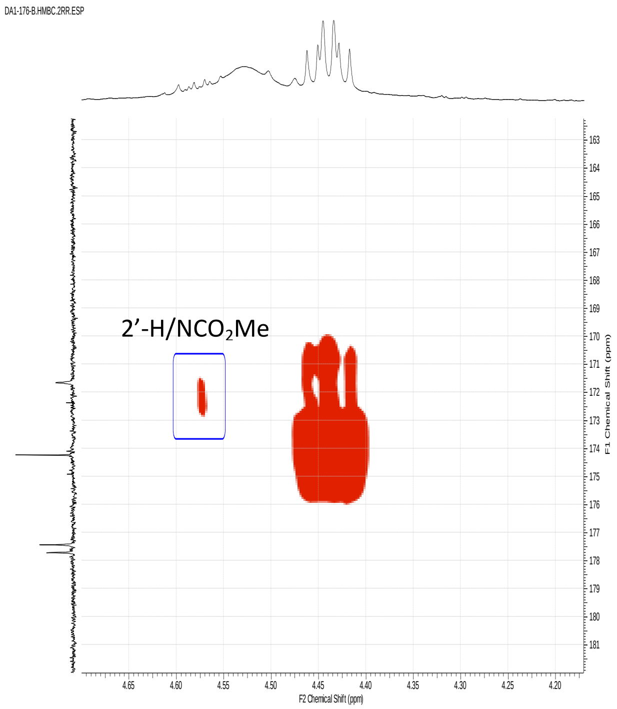

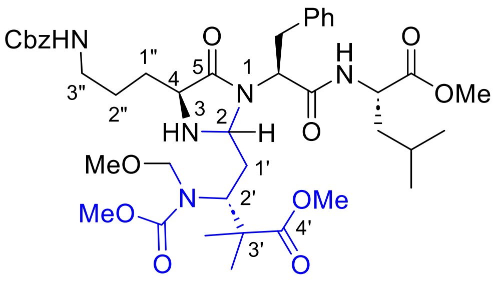

27

NOESY

70 °C, CD_3CN

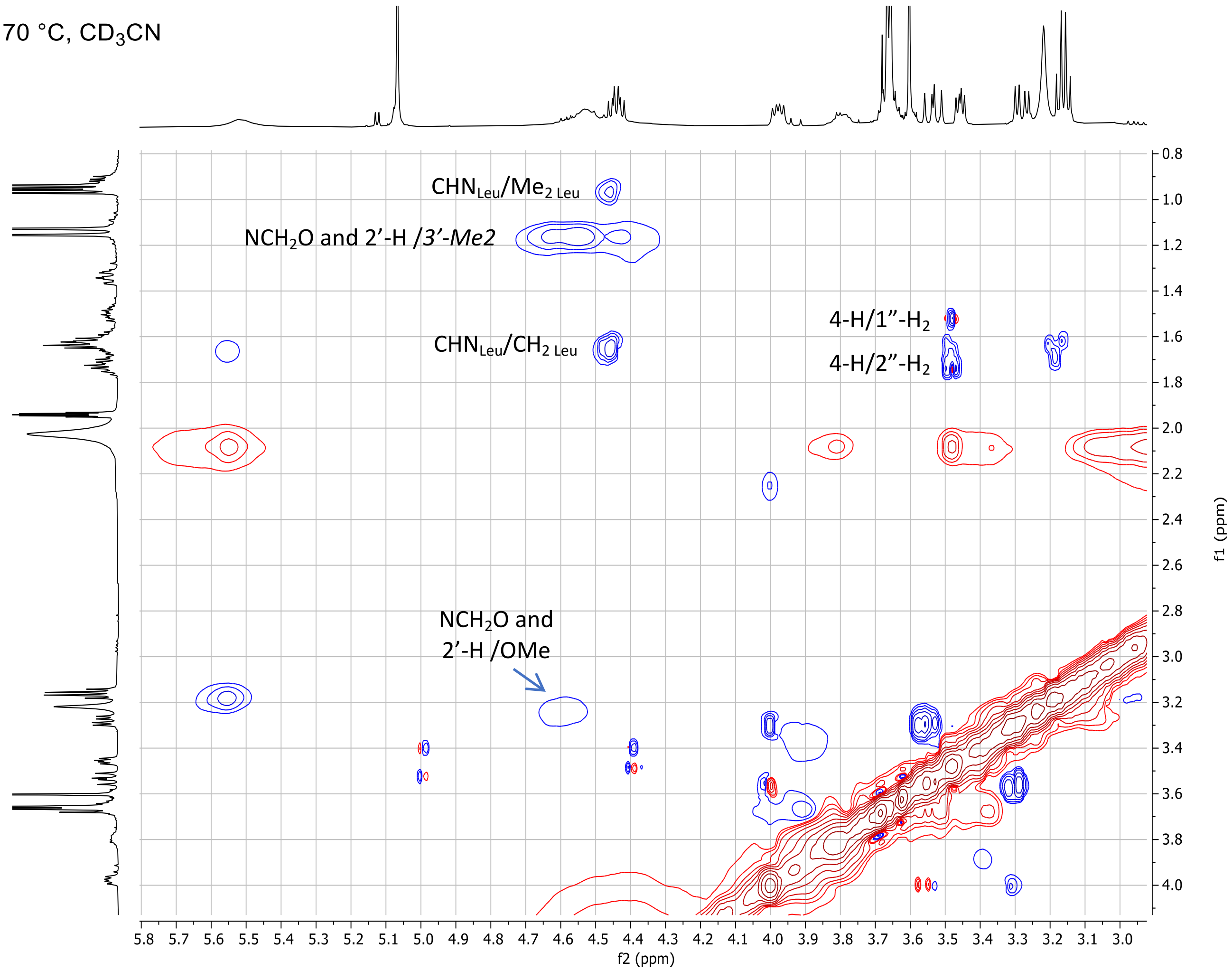

THEORETICAL DIHEDRAL ANGLES OF COMPOUNDS 26 AND epi-26

The theoretical dihedral angles of compounds **26** and **epi-26** for coupling constant calculations (see ref. 19 in Manuscript) are shown below. The angles were calculated with Chem3D Pro (64bit), version 22.0.0.22, from Perkin Elmer, using the MM2 tool for the determination of minimum-energy conformations of compounds **26** and **epi-26**, and their key dihedral angles (default parameters).

Compound 26

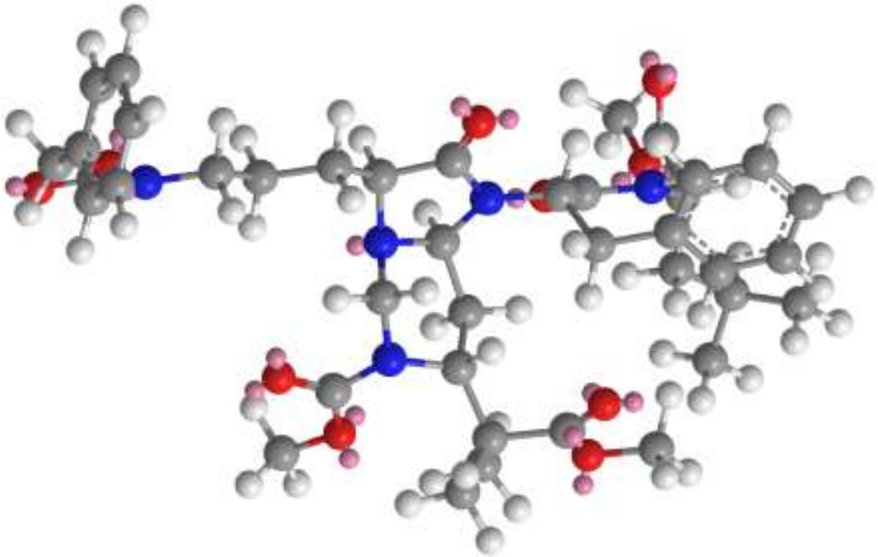

Compound **26**

Total energy= 44,9250 kcal/mol

7-H/8-Hβ…65°

7-H/8-Hα…180°

8a-H/8-Hβ…80°

8a-H/8-Hα…36°

Compound (8a-epi)-26

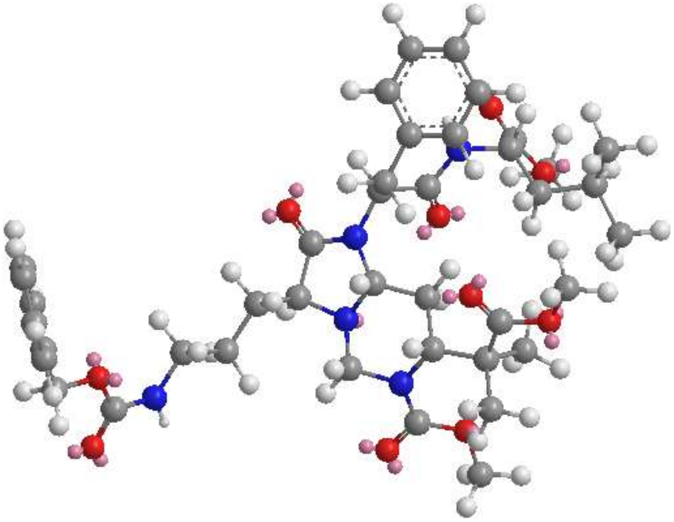

Compound epi-**26**

Total energy = 48,8259 kcal/mol

7-H/8-Hβ…73°

7-H/8-Hα…168°

8a-H/8-Hβ…66,5°

8a-H/8-Hα…174,5°
